# Isolable Diaminophosphide Boranes

**DOI:** 10.1002/chem.202002296

**Published:** 2020-10-19

**Authors:** Markus Blum, Tobias Dunaj, Julius A. Knöller, Christoph M. Feil, Martin Nieger, Dietrich Gudat

**Affiliations:** ^1^ Institute of Inorganic Chemistry University of Stuttgart Pfaffenwaldring 55 70550 Stuttgart Germany; ^2^ Department of Chemistry University of Helsinki P.O Box 55 00014 University of Helsinki Finland

**Keywords:** alkali metals, boranes, nucleophiles, phosphines, phosphorus nitrogen compounds

## Abstract

Metalation of secondary diaminophosphine boranes by alkali metal amides provides a robust and selective access route to a range of metal diaminophosphide boranes M[(R_2_N)_2_P(BH_3_)] (M=Li, Na, K; R=alkyl, aryl) with acyclic or heterocyclic molecular backbones, whereas reduction of a chlorodiaminophosphine borane gave less satisfactory results. The metalated species were characterized in situ by NMR spectroscopy and in two cases isolated as crystalline solids. Single‐crystal XRD studies revealed the presence of salt‐like structures with strongly interacting ions. Synthetic applications of K[(R_2_N)_2_P(BH_3_)] were studied in reactions with a 1,2‐dichlorodisilane and CS_2_, which afforded either mono‐ or difunctional phosphine boranes with a rare combination of electronegative amino and electropositive functional disilanyl groups on phosphorus, or a phosphinodithioformate. Spectroscopic studies gave a first hint that removal of the borane fragment may be feasible.

## Graphical abstract

**It′s just so plain borane**: Alkali metal diaminophosphide boranes, which can be selectively prepared by metalation of PH‐substituted precursors with non‐nucleophilic amides, were for the first time isolated and characterized crystallographically. Their synthetic application as highly functional nucleophilic building blocks is illustrated in metatheses with a dichlorodisilane and the formation of a stable carbon disulfide adduct.

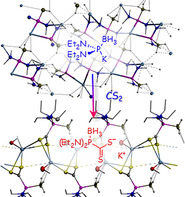

## Introduction

**Scheme 1:**
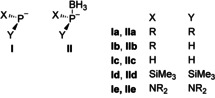
Generic molecular structures of phosphide (**I**) and phosphide borane (**II**) anions (R=alkyl, aryl).

Coupling of nucleophilic metal diorganophosphides **I** or diorganophosphide boranes **II** (Scheme 1) with suitable electrophiles is one of the prime routes for the synthesis of phosphines.^[1]^ The borane unit in **II** serves both as a protecting and activating group that suppresses on one hand unwanted side reactions like the formation of phosphonium ions, and may on the other hand facilitate phosphide formation, for example, by boosting the PH‐acidity of a phosphine precursor.^[2]^

Typical phosphide reagents carry usually chemically inert alkyls or aryls (**Ia**/**IIa**),^[3]^ but the presence of hydrides (**Ib,c**/**IIb,c**)^[4]^ or substituents based on heavier group‐14 elements (often Me_3_Si, **Id** ^[5]^) is also not uncommon. Primary, (**Ib**/**IIb**) parent (**Ic**/**IIc**) and silylated phosphides (**Id**) are special because their ability to undergo electrophilic post‐functionalization of reactive P−H and P−Si bonds after the initial metathesis step makes them essentially polyfunctional building blocks.

In contrast to **Ia‐d**/**IIa‐d**, amino‐substituted phosphide derivatives **Ie**/**IIe** have only received scarce attention. Like their congeners **Ic,d**/**IIc**, these species can serve as nucleophilic reagents for the synthesis of functional phosphines with a possibility for further derivatization. However, whereas the phosphorus atom in primary or silylated phosphines accessible from **Ic,d**/**IIc** retains its nucleophilic character, the electronegative substituents in diaminophosphines generated from **Ie**/**IIe** impose electrophilic character on the phosphorus atom transferred, which allows for post‐functionalization by nucleophiles rather than electrophiles. In this respect, diaminophosphide reagents may be considered as tools that allow coupling an electrophilic R_2_P‐fragment with an electrophilic substrate, which makes them synthetic building blocks whose reactivity complements that of primary or silylated phosphides, respectively.

**Scheme 2:**
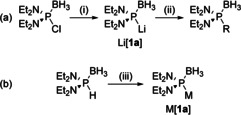
Previously reported preparation of lithium and potassium bis(diethylamino)phosphide boranes M[**1 a**] (M=Li, K).[^9^, ^10^] Reagents and conditions: (i) 2 Li(C_10_H_8_), THF, −78 °C to RT; (ii) RX, −78 °C to RT (X=halide, mesylate); (iii) *n*BuLi or KHMDS, Et_2_O, RT.

Whereas free diaminophosphides **Ie** remain still elusive species,^[6]^ some progress has recently been made in the field of their complexes with borane or transition metals, respectively. Transition metal complexes [(R_2_N)_2_P(Fe(CO)_4_]K^[7]^ (**III**, R=Et, Ph) and [(Me_2_N)(Ph_3_C)P(W(CO)_5_]K^[8]^ (**IV**) containing metal‐stabilized mono‐ or diamino‐phosphido ligands were generated as spectroscopically detectable entities by deprotonation of PH‐substituted precursor complexes and shown to react as P‐centered nucleophiles. The significance of diaminophosphide boranes as synthetic intermediates was first recognized by the group of Knochel,^[9]^ who prepared diaminophosphine boranes by electrophilic alkylation or arylation of an intermediary lithium diaminophosphide borane Li[**1 a**] that had been generated in situ by lithium reduction of a chlorophosphine borane precursor but was neither positively identified nor further characterized (Scheme 2 a). We have recently shown that monomeric potassium (K[**1 a**]) and lithium diethylaminophosphide borane (Li[**1 a**]) can be alternatively accessed through metalation of a secondary diaminophosphine borane precursor (Scheme 2 b), and reported on the first spectroscopic characterization of such species as well as on their transmetalation with zinc and copper halides to afford isolable transition metal complexes.^[10]^

Striving to further establish diaminophosphide boranes as well‐defined synthetic tools, we describe here the preparation of reagents with an extended range of amino substituents, including first heterocyclic derivatives, and alkali metal ions, the isolation and crystallographic characterization of sodium and potassium dimethylaminophosphide boranes, and selected reactions with electrophiles which extend the application of the nucleophilic reagents to the synthesis of highly functionalized diaminosilyl phosphines and the activation of a cumulene.

## Results and Discussion

### Synthesis and spectroscopic characterization of diaminophosphide boranes

**Scheme 3:**
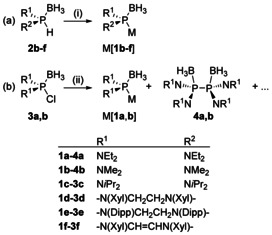
Synthesis of metal diaminophosphide boranes M[**1 b**–**f**] (M=Li, Na, K; Dipp=2,6‐di(isopropyl)phenyl), Xyl=2,6‐Xylyl. Reagents and conditions: (i) 1.2 equiv. MHMDS, LDA, *n*BuLi, or MeMgCl, Et_2_O or THF, RT; (ii) see Table 1.

To deepen our insight into the formation of diaminophosphide boranes, we tested the routes reported for accessing Li[**1 a**], viz. reductive metalation of the diaminochlorophosphine borane^[9]^ and deprotonation of the secondary diaminophosphine borane,^[10]^ with a larger range of structurally diverse substrates. The precursors **2 b**–**f** and **3 b** (Scheme 3) were prepared following known procedures[^9^, ^10^, ^11^] and characterized by spectroscopic and analytical data (see Experimental Section). The synthesis of **2 b** imposed some problems, since the previously reported reaction of (Me_2_N)_2_PCl and LiBH_4_ ^[12]^ gave in our hands unsatisfactory results, and a modified approach involving pre‐complexation of (Me_2_N)_2_PCl with Me_2_S⋅BH_3_ (see Experimental Section) furnished only inseparable mixtures of **2 b** with Me_2_NH⋅BH_3_ (10–20 mol %). As the by‐product did not interfere with the subsequent metalation reactions, the mixtures were used without further purification. Reactions aiming at the generation of phosphides were carried out in THF, diethyl ether, or mixtures thereof, and reaction monitoring and product identification was generally achieved in situ by multinuclear (^1^H, ^31^P, ^11^B) NMR spectroscopy. We found that reliable and highly selective generation (with 90–99 % conversion by integration of suitable NMR signals) of phosphide boranes M[**1 b**–**f**] was feasible by treating the PH‐substituted precursors **2 b**–**e** with a slight excess of a non‐nucleophilic alkali amide such as lithium di(isopropyl)amide (LDA) or a metal hexamethyldisilazide (MHMDS=MN(SiMe_3_)_2_, M=Li, Na, K, Scheme 3 a), respectively. The functioning of the silylamides as universally applicable bases allows selecting the alkali metal in such a way that subsequent metathesis of the phosphide reagent with electrophiles gives rise to an easily separable salt. Under practical aspects, we consider KHMDS a particularly useful reagent because it is commercially available in sufficient quality, and potassium halides, which are formed as by‐products in many follow‐up reactions of the phosphides, are insoluble in common organic solvents. Metalation of **2 b** with NaHDMS in a THF/ether mixture and with KHMDS in the presence of a crown ether permitted also the isolation of first crystalline diaminophosphide boranes of composition Na[**1 b**]⋅THF and K[**1 b**] (the crown ether was in this case not incorporated in the crystal), respectively. Both products were characterized by single‐crystal X‐ray diffraction studies (see further below).

Moreover, using KHMDS for deprotonation enabled both the generation of a sterically congested diaminophosphide reagent like K[**1 c**] and heterocyclic derivatives like K[**1 d**], K[**1 e**], while selective lithiation of heterocyclic secondary diaminophosphine borane **2 e** was best accomplished with LDA. Metalation of the secondary diazaphospholene borane **2 f** turned out a borderline case. Treatment with KHMDS produced detectable amounts of the expected, highly moisture and air sensitive phosphide K[**1 f**], which was identified by spectroscopic data and chemical trapping with a proton source. However, both deprotonation and recovery of the secondary phosphine borane were in this case unselective and produced side‐products that could neither be successfully identified nor separated. We relate the difficulties encountered in the generation of K[**1 f**] to the unusual electronic structure of unprotected secondary diazaphospholenes, which display a hydride reactivity that contrasts the behavior of alkyl and arylphosphines and is associated with an “Umpolung” of the P−H bond.^[13]^ Obviously, this effect is subdued by the borane coordination to make room for a protic reactivity that is unprecedented for diazaphospholenes, but the lability of the resulting anion implies that the P‐H acidity remains low.

Application of other deprotonating reagents than amides gave erratic results. Metalation with *n*‐butyl lithium, which had been employed in the synthesis of Li[**1 a**],^[10]^ enabled also preparing the sterically more congested acyclic phosphide borane Li[**1 c**] but proceeded unselectively and irreproducibly with heterocyclic substrates. The use of Grignard reagents was tested with sterically uncongested diaminophosphine boranes **2 a**,**b**. While MeMgCl converted both substrates cleanly into the corresponding phosphides, EtMgBr was found to be unreactive.

**Scheme 4:**
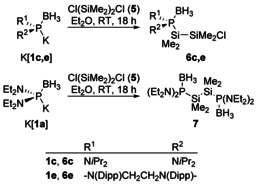
Reactions of potassium diaminophosphide boranes with 1,2‐dichloro‐tetramethyldisilane **5**.

Exploring the reduction of chlorophosphine boranes **3 a**,**b** ^[9]^ as an alternative to the metalation of the secondary phosphine boranes (Scheme 3 b) revealed that pure alkali metals are in most cases unreactive (see Table 1). A single exception was observed for the reaction of potassium with **3 b**, which afforded a low yield of the metalation product K[**1 b**] along with a main product later identified^[14]^ as the diphosphine bis‐borane complex **4 b** (Scheme 4) and further unidentified by‐products. Further transformations became feasible when soluble metal naphthalenides rather than solid metals were used as reductants. However, while reduction of **3 a** with lithium naphthalenide afforded, in accord with earlier reports,^[9]^ a near quantitative yield of Li[**1 a**], reactions of **3 b** with lithium and potassium naphthalenides gave a product mixture which contained the expected metal phosphide boranes along with diphosphine bis(borane) **4 b** and substantial amounts of unidentified by‐products. We have currently no concise explanation for the varying selectivity, even if it is tempting to relate the observed changes to different sterics of the substrates.

**Table 1:** Reduction of diaminochlorophosphine boranes **3 a**,**b**.

Compound	Reductant	Conditions	Conversion [%] to ^[a]^	
			M[**1 a,b**]	**4**
**3 a**	Li	[D_8_]THF, RT	n.r. ^[b]^	
**3 a**	Li(C_10_H_8_)	Et_2_O, −78 °C to RT	>99	
**3 a**	Na	[D_8_]THF, RT	n.r.	
**3 a**	K	[D_8_]THF, RT	n.r.	
**3 b**	Li	[D_8_]THF, RT	n.r.	
**3 b**	Li(C_10_H_8_)	THF, RT	21	25
**3 b**	Na	[D_8_]THF, RT	n.r.	
**3 b**	K	[D_8_]THF, RT	18	63
**3 b**	K(C_10_H_8_)	THF, RT	11	25

[a] As fraction of the total spectral integral of the ^31^P{^1^H} NMR spectra of the reaction mixtures. [b] n.r.=no reaction.

The diagnostically most valuable information for the identification of metalated diaminophosphide boranes M[**1 b**–**f**] comes from ^31^P and ^11^B NMR spectra. The signals display the expected multiplet structures arising from spin‐coupling between ^11^B (I=3/2) and ^31^P (I=1/2) nuclei and lack a characteristic splitting due to ^1^ *J*_PH_ coupling across a P−H bond. As previously noted^[10]^ for Li[**1 a**] and K[**1 a**], the ^31^P NMR chemical shifts of specimens with identical diaminophosphide units show metal‐dependent changes, and the presence of a cation sequestering reagent like dibenzo‐18‐crown‐6 induces increases in *δ* ^31^P of potassium phosphide boranes K[**1 c**,**e**] by 10 to 15 ppm. Both effects suggest the presence of tight contact ion pairs in solution. The non‐uniform trends observed (formal displacement of a light metal ion by a heavier homologue induces an increase in *δ* ^31^P for M[**1 a**] and M[**1 e**] but a decrease for M[**1 b**] and M[**1 c**]; M=Li, Na, K) discredit a simplistic assumption that declining covalency in the phosphorus‐metal bonding with growing size of the metal ion induces a deshielding of the ^31^P nucleus. The ^11^B NMR signals of M[**1 a**–**f**] display positive “metalation shifts” (Δ*δ* ^11^B +5 to +11 ppm) and numerically reduced ^1^ *J*_P11B_ coupling constants relative to the corresponding secondary phosphine borane precursors **2 a**–**f**. The same trends hold for dialkyl and diarylphosphide boranes (Table 2). On the contrary, metalation shifts in ^31^P NMR spectra are positive rather than negative as in alkyl and arylphosphide boranes[^15^, ^16^] and show large structure‐related variations (Table 2). Particularly large displacements prevail for heterocyclic compounds (Δ*δ* ^31^P +95 to +122 ppm for M[**1 d**–**f**]; M=Li, K) and an unusually low shift for sterically congested M[**1 c**] (Δ*δ* ^31^P +7 to +15 ppm; M=Li, K). A concise explanation for these effects is yet lacking.

**Table 2:** ^11^B and ^31^P NMR data of phosphide boranes (at RT in THF or [D_8_]THF).

Compound	*δ* ^31^P^[a]^ [ppm]	*δ* ^11^B^[a]^ [ppm]	^1^ *J*_BP_ [Hz]^[b^
Li[**1 a**]^[c]^	128.0 (47.8)	−34.1 (5.0)	56 (−18)
K[**1 a**]^[c]^	130.7 (50.5)	−32.4 (6.7)	53 (−21)
Li[**1 b**]	145.9 (56.7)	−34.6 (5.6)	32 (−40)
Na[**1 b**]	145.0 (55.8)	−34.3 (5.9)	49 (−23)
K[**1 b**]	142.9 (53.7)	−32.9 (7.3)	49 (−23)
(MgCl)[**1 b**]	142.4 (53.2)	−33.0 (7.2)	49 (−23)
Li[**1 c**]	61.8 (15.5)	−32.9 (2.7)	62 (−10)
K[**1 c**]	53.4 (7.1)	−29.5 (6.1)	64 (−8)
[K(dibenzo‐18‐crown‐6)][**1 c**]	68.5 (22.2)	−30.4 (5.2)	53 (−19)
K[**1 d**] ^[d]^	170.6 (95.3)	−28.6 (11.2)	28 (−39)
K[**1 f**]	201.6 (121.8)	−29.5 (10.7)	24 (−26)
Li[**1 e**]	185.7 (103.9)	−32.2 (8.1)	29 (–)^[e]^
K[**1 e**]	187.7 (105.9)	−29.9 (10.4)	26 (–)^[e]^
[K(dibenzo‐18‐crown‐6)][**1 e**]	197.3 (115.5)	−29.8 (10.5)	23 (–)^[e]^
Li(tmeda)[PMe_2_]^[f]^	−96.1 (−72.1)	−24.2 (13.8)	42 (−16)
K[P(*t*‐Bu)_2_]^[g]^	12.8 (−36.4)	−34.7 (9.0)	34 (−17)
K[PPh_2_]^[g]^	−28.8 (−30.5)	−30.1 (9.9)	32 (−10)

[a] Difference *δ*([M[R_2_P(BH_3_)]−*δ*(R_2_PH(BH_3_) in parentheses. [b] For ^11^B isotopomer; difference |^1^ *J*_PB_(M[R_2_P(BH_3_)])|−|^1^ *J*_PB_(R_2_PH(BH_3_))| in parentheses. [c] Data from Ref. [10]. [d] In C_6_D_6_. [e] Splitting in R_2_PH(BH_3_) unresolved. [f] Data from Ref. [15]. [g] Data from Ref. [16].

### Crystallographic studies

**Figure 1:**
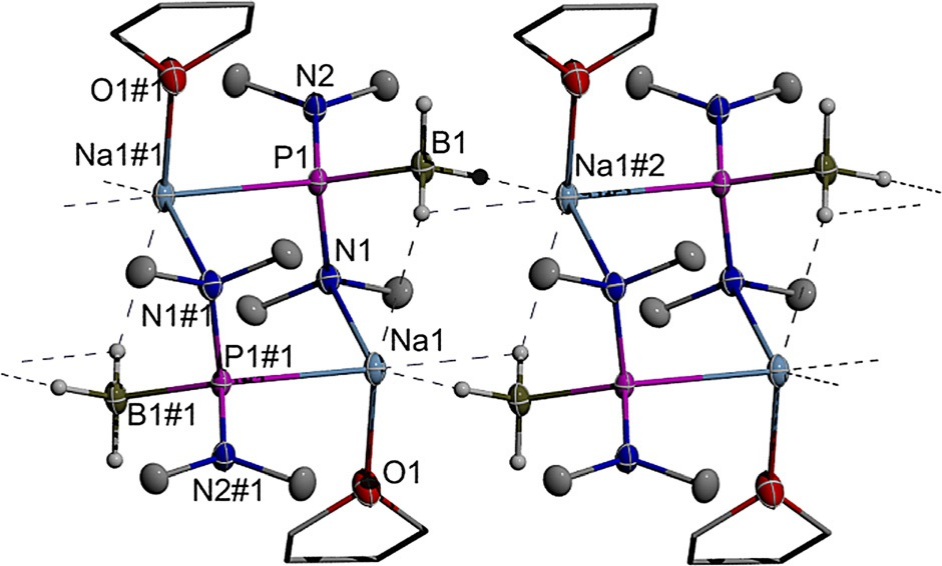
Graphical representation of two dimeric units in crystalline Na(THF)[**1 b**]. For clarity, hydrogen atoms except those of BH_3_ groups were omitted and carbon atoms in THF ligands represented using a wire model. Thermal ellipsoids of heavy atoms were drawn at the 50 % probability level. Selected distances (in Å): P1−N1 1.752(1), P1−N2 1.699(1), P1−B1 1.937(2), P1−Na1#1 2.963(1), Na1−O1 2.311(1), Na1−N1 2.520(1), B1−Na1#2 2.682(2), B1−Na1 3.361(2).

**Figure 2:**
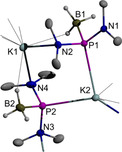
Graphical representation of the crystallographically independent formula units in crystalline K[**1 b**]. For clarity, hydrogen atoms except those of BH_3_ groups were omitted. Thermal ellipsoids of heavy atoms were drawn at the 50 % probability level. Selected distances (in Å): P1−N1 1.697(2), P1−N2 1.741(2), P1−B1 1.947(3), P1−K2 3.301(1), P2−N3 1.736(2), P2−N4 1.725(2), P2−B2 1.935(4), P2−K2 3.352(1), K1−N2 2.849(2), K1−N4 2.932(2), K1−H(B1) 2.86(3), K1−H(B2) 2.72(3), K2−N3#1 2.961(2).

Graphical representations of the results of single‐crystal X‐ray diffraction studies on triclinic Na(THF)[**1 b**] (space group *P* $\bar{1}$ ) and monoclinic K[**1 b**] (space group *P*2_1_/*c*) and important metrical parameters are displayed in Figures 1, 2, and S1 (Supporting Information). Full crystallographic data are included in the Supporting Information.

Crystals of Na(THF)[**1 b**] contain dimeric units assembled from two anionic diaminophosphide borane and two cationic Na(THF) fragments (Figure 1). The metal cations connect to the phosphorus atom of one and a nitrogen atom of the other anion fragment in the same unit, resulting in the formation of a centrosymmetric six‐membered cyclic array with a typical chair conformation. In addition, each metal ion features contact to two hydrides of a η^2^‐bound borane unit in a neighboring dimer (Na−H 2.38(2) to 2.39(2) Å) and a weaker contact to a B−H bond of the adjacent borane in the same unit (Na‐H 2.69(2) Å). Interactions of this type have precedence in the structures of alkyl/arylphosphide boranes[^15^, ^17^] and link in Na(THF)[**1 b**] the dimeric units to form one‐dimensionally infinite strands aligned parallel to the crystallographic *a*‐axis. If we count the η^2^‐bound borane as one ligand, the metal ions exhibit an irregular pseudo‐[4+1] coordination geometry in which the weak “intramolecular” B−H interaction is aligned roughly opposite to the oxygen atom of the THF ligand. The phosphorus atom displays a distorted tetrahedral coordination with bond angles from 103.1(1)° (N1−P1−B1) to 118.8(1)° (Na1−P1−B1). The P−B and P−N distances are in the range of single bonds, with the deviation of 0.053(2) Å between the P−N distances reflecting the different coordination numbers of the nitrogen atoms. While the Na1−O1 distance of 2.311(1) Å is a close match to the sum of covalent radii (2.32(11) Å^[18]^) and may be viewed as regular coordinative bond, the Na1−N1 (2.5204(12) Å) and Na1−P1 distances (2.9625(6) Å) perceptibly exceed this sum (Na−N 2.37(10) Å, Na−P 2.73(12) Å^[18]^), suggesting a description as close inter‐ionic contacts.

Crystalline K[**1 b**] contains two independent anionic units and two cations which aggregate, as in the sodium analogue, via K⋅⋅⋅N and K⋅⋅⋅P interactions to form strongly puckered six‐membered cyclic arrays (Figure 2). However, whereas the inversion symmetry in Na(THF)[**1 b**] imposes an head‐to‐tail alignment of the N,P‐units that renders both sodium ions equivalent, the anion fragments in the potassium salt are arranged in a head‐to‐head fashion, with both phosphorus atoms binding to one and both nitrogen atoms to the other metal ion. Additional interaction of the P_2_‐ligated potassium ion with an exocyclic dimethylamino group of a neighboring unit gives rise to the formation of a coordination polymeric assembly of dimeric units with a zig‐zag chain of K⋅⋅⋅N/K⋅⋅⋅P interactions and covalent P−N bonds as backbone (Figure S1). The coordination sphere of the linking metal ion is completed by additional contacts to a η^3^‐bound borane unit (H⋅⋅⋅K2 2.645(27) to 2.762(31) Å) from the second next dimeric unit in the row, which reinforce the supramolecular assembly and give rise to the formation of a double stranded array of heterocyclic units extending along the crystallographic *b*‐axis. Completion of the coordination sphere of the N_2_‐coordinated potassium ion is accomplished by weaker BH⋅⋅⋅K interactions (H⋅⋅⋅K1 2.69(3) to 3.04(3) Å) with borane units from adjacent, parallel strands. These secondary interactions result ultimately in the formation of two‐dimensional layers, which extend in the crystallographic *a,b*‐plane and are held together by van‐der‐Waals interactions. The P−N (1.697(2) to 1.741(2) Å) and P−B distances (1.935(4), 1.947(3) Å are similar as in Na(THF)[**1 b**] and correspond to single bonds. The P⋅⋅⋅K (3.301(1), 3.352(1) Å) and N⋅⋅⋅K distances (2.849(2) to 2.961(2) Å) exceed the reported sum of covalent radii (P−K 3.10(15), N−K 2.74(13) Å^[18]^), but the former match the distances in known potassium dialkyl and diarylphosphides (P−K 3.309±0.071 Å^[19]^).

### Reactions with electrophiles

The synthetic potential of diaminophosphide boranes has so far been demonstrated in reactions under alkylation or arylation with organic electrophiles,^[9]^ and in metatheses with transition metal salts leading to the formation of borane‐supported terminal transition metal phosphides.^[10]^ Seeking to further widen the synthetic bandwidth, we considered silyl‐substituted diaminophosphines another rewarding class of target molecules. While the presence of two types of functional substituents with opposite polarity makes these compounds, like the phosphides, potentially highly valuable building blocks, they are only rarely reported in the literature.^[20]^ Known syntheses involve mostly reactions of diaminochlorophosphines with silanides,[^20a^, ^20b^, ^20e^] or reductive coupling of diaminochlorophosphines with chlorotrimethylsilane,^[20c]^ respectively. Cross coupling of P‐based nucleophiles with electrophilic organosilicon compounds can thus provide a new, chemically complementary access route.

To explore the feasibility of this approach, we studied reactions of diaminophosphide boranes with commercially available 1,2‐dichloro‐1,1,2,2‐tetramethyldisilane **5** as prototype of a potentially multifunctional silicon‐based electrophile. Potassium phosphide boranes emerged as the best suited reagents under practical aspects, as the nucleophiles can easily be generated in situ by treating the secondary phosphine borane precursors with a slight excess of KHMDS, and potassium chloride formed as by‐product is easily and quantitatively removed by filtration. The reactions of **5** with K[**1 c**] and K[**1 e**] afforded exclusively monosubstitution products (**6 c**,**e**), even when the nucleophile was employed in excess (Scheme 4). In contrast, a clean reaction with K[**1 a**] required employing two equivalents of the nucleophile and afforded the expected 1,2‐bisphosphino‐disilane **7**. The failure to obtain analogous disubstitution products from K[**1 c,e**] is presumably due to the increased steric demand of these reagents. All three products were isolated as thermally stable, moisture sensitive oils (**6 c**) or crystalline solids (**6 e**, **7**) and characterized by analytical and spectroscopic data. The ^1^H, ^11^B, ^13^C and ^31^P NMR spectra are unremarkable and confirm the proposed constitution. The ^29^Si NMR spectrum of **7** is peculiar as the expected multiplet (representing the X‐part of an ABX spin system) degenerates accidentally to a simple doublet. One of the two expected ^29^Si NMR signals of **6 e** stayed undetected for unknown reasons.

**Figure 3:**
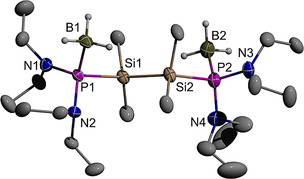
Graphical representation of one of two crystallographically independent molecules in crystalline **7**. For clarity, hydrogen atoms except those of BH_3_ groups were omitted, and only one of two disordered orientations of three disordered Et‐groups drawn. Thermal ellipsoids of heavy atoms were drawn at the 50 % probability level. Selected distances (in Å) and torsional angles (in °): P1−N1 1.676(2), P1−N2 1.678(2), P1−B1 1.927(3), P1−Si1 2.289(1), Si1−Si2 2.363(1), Si2−P2 2.299(1), P2−N3 1.677(2), P2−N4 1.660(2), P2−B2 1.934(3), P1−Si1−Si2−P2 176.28(4).

**Figure 4:**
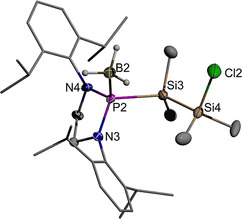
Graphical representation of one of two crystallographically independent molecules in crystalline **6 e**. For clarity, hydrogen atoms except those of BH_3_ groups were omitted, carbon atoms in the *N*‐Dipp substituents represented using a wire model, and only one of two disordered orientations of a disordered *i*Pr‐group drawn. Thermal ellipsoids of heavy atoms were drawn at the 50 % probability level. Selected distances (in Å) and torsional angles (in °): P2−N3 1.699(3), P2−N4 1.695(3), P2−B2 1.919(4), P2−Si3 2.307(1), Si3−Si4 2.378(2), Si4−Cl2 2.089(2), P2−Si3−Si4−Cl2 −98.1(1).

The molecular structures of **6 e** and **7** were further confirmed by single‐crystal X‐ray diffraction studies. Crystals of **7** (Figure 3) contain two independent molecules which both display positional disorder in the peripheral ethyl groups (see Experimental Section). Both specimens adopt a staggered conformation of the central disilane unit with a *transoid* arrangement of the two phosphine borane units (P−Si−Si−P 176.3(1)° and 180°, Figures 3, S2). The unit cell of **6 e** holds likewise two independent molecules, both of which exhibit, however, a nearly eclipsed conformation around the Si−Si bond. The chlorine and phosphorus atoms in one molecule adopt a *gauche*‐orientation (Figure 4), while the peripheral atoms of the terminal SiMe_2_Cl unit in the second molecule display a disorder indicating the presence of a 35:65 mixture of two rotamers with *gauche*‐ and *trans*‐aligned Cl and P atoms (Figure S3). The N‐heterocyclic rings exhibit similar twist conformations as in borane‐free diazaphospholidines^[21]^ and diazaphospholidine oxides.^[22]^ The P−Si and Si−Si distances in **7** are unremarkable and suggest the presence of normal single bonds. The P−N distances in both complexes are shorter than in free acyclic or N‐heterocyclic phosphines,[^11^, ^13^, ^21^] indicating a similar bond strengthening upon borane coordination as had previously been observed for secondary diazaphospholidines.[^11^, ^21^]

The 1,2‐diphosphino‐disilane representing the backbone of **7** can be considered a heavier diphosphinoethane homologue. Scattered reports on template syntheses of diphosphino–disilane transition metal complexes^[23]^ imply that such species can in principle, like their lighter congeners, act as chelating ligands. Aiming at making an aminofunctionalized 1,2‐diphosphino‐disilane available for complexation studies, we set out to study de‐protecting **7** by sequestering the borane units with 1,4‐diazabicyclo[2.2.2]octane (DABCO).^[24]^ Heating a hexane solution of both reactants to 60 °C produced a colorless precipitate that was identified by ^11^B NMR spectroscopy as the expected bis‐borane adduct of DABCO (*δ* ^11^B −10.9 ppm, ^1^ *J*_BH_=99 Hz^[25]^). The ^31^P NMR spectrum displayed a main signal (*δ* ^31^P 96.4 ppm with 56 % of the total integrated intensity) which lacks the characteristic broadening arising from spin‐coupling to a quadrupolar ^11^B nucleus and is tentatively assigned to the targeted borane‐free diphosphino‐disilane. In addition, signals attributable to unknown borane‐free phosphorus‐containing species (*δ* ^31^P 94.2 and 94.5 ppm with integrated intensities of 9 and 16 %, respectively) and bis(diethylamino)phosphine (*δ* ^31^P 78.6 ppm, ^1^ *J*_PH_=250 Hz, 19 %) were detected, and the ^11^B NMR spectrum revealed further the presence of residual **7** (the ^31^P NMR signal of which was presumably buried in spectral noise). The product distribution observed can be explained by assuming that de‐protection is in principle feasible but remains incomplete and is accompanied by P−Si bond cleavage arising from adventitious hydrolysis. Attempts to isolate any of the de‐protected phosphorus‐containing products remained unsuccessful.

**Scheme 5:**
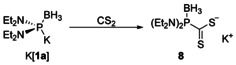
Reaction of K[**1 a**] with CS_2_.

Expecting that diaminophosphide boranes should like unprotected secondary diorganophosphines or ‐phosphides be capable of undergoing nucleophilic attack on the electrophilic carbon center of cumulenes,^[26]^ we studied further the reactions of K[**1 a**] with carbon dioxide and carbon disulfide. While the former gave rise to an intractable colorless solid that could as yet not be unambiguously characterized, the reaction with CS_2_ proceeded cleanly to afford a soluble species which was isolated as a red, air and moisture stable crystalline solid after work‐up and recrystallization from ether/pentane. Analytical and MS data are in accordance with the formation of phosphinodithioformate borane **8** (Scheme 5), the constitution of which was straightforwardly confirmed by a single‐crystal X‐ray diffraction study on a hemi‐hydrate **8**⋅0.5 H_2_O.

**Figure 5:**
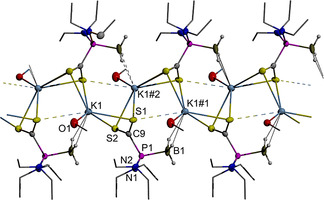
Graphical representation of a section of the crystal structure of **8** showing the formation of a coordination polymer along the crystallographic *b*‐axis. Hydrogen atoms except those of BH_3_ groups were omitted, carbon atoms in the NEt substituents represented using a wire model, and only one of two disordered orientations of the Et‐groups on N2 was drawn. Thermal ellipsoids of heavy atoms were drawn at the 50 % probability level. Selected distances (in Å): P1−N1 1.653(4), P1−N2 1.663(3), P1−C9 1.848(4), P1−B1 1.915(6), C9−S2 1.680(4), C9−S1 1.687(4), S1−K1 3.2309(15) S1−K1#1 3.2782(16), S2−K1 3.2724(15), S2−K1#1 3.4346(15), K1−O1 2.820(2).

The crystal is composed of an array of potassium cations, complex anions and water molecules which is structured by a network of mutual non‐covalent interactions with distances that are intermediate between sums of covalent and van‐der‐Waals radii (Figure 5). The basic motif in this layout consists of one‐dimensional stacks of alternating cations and anions stretching along the crystallographic *b*‐axis. The connection of adjacent cations in each stack by μ_2_‐bridging, 1:2κ^2^ *S*;1:2κ^2^ *S′*‐coordinated anions gives rise to a zig–zag chain‐like arrangement that is reinforced by weaker S⋅⋅⋅K and BH⋅⋅⋅K contacts linking one of the sulfur atoms and the borane unit of an specific μ_2_‐bridging anion with a third metal cation. The individual strands align parallel to each other and are mutually connected by water molecules that bind in a μ_2_‐bridging fashion to two metal cations in adjacent strands. The grid of K⋅⋅⋅O⋅⋅⋅K linkages thus formed connects the one‐dimensional ion stacks to two‐dimensional layers that extend in the crystallographic *b,c*‐plane and are held together by van‐der‐Waals interactions between the diethylamino groups.

Considering all interactions together, the potassium ions can be assigned a [5+3] coordination sphere arising from close contacts to the sulfur atoms of two μ_2_‐bridging phosphinodithioformate anions and a water molecule (K⋅⋅⋅S (3.231(2) to 3.435(2) Å, K⋅⋅⋅O (2.820(2) Å), and weaker contacts to a remote sulfur and two hydrogen atoms of a η^2^‐coordinated borane unit (K⋅⋅⋅S 3.806(2) Å, K⋅⋅⋅H 2.62(6), 2.85(6) Å). The anion is built around a phosphorus atom with distorted tetrahedral and a carbon atom with planar coordination. The P−N (1.653(4), 1.663(3) Å) and P−B (1.915(6) Å) distances are still shorter than in **6 e**, **7** but remain in the range of single bonds. The P−C (1.849(4) Å) and C−S (1.680(54), 1.687(4) Å) distances match those reported for zwitterionic triaminophosphonio dithioformates (R_2_N)_3_P^+^–CS_2_^−^ (P−C 1.848(4), 1.849(2) Å; C−S 1.651(4) to 1.675(3) Å^[27]^) and a cationic ester (R_2_N)_3_P^+^–CS_2_Me (P−C 1.852(5) Å^[28]^). Even if a slightly longer distance has been observed for a neutral species (R_2_N)_2_P–CS_2_–P(NR_2_)_2_ containing a tri‐coordinate P atom (P−C 1.895(4) Å^[29]^), our findings are in accord with the presumption that the P−C and C−S distances in the PCS_2_ fragment are rather insensitive to the nature of the P substituents.^[30]^

## Conclusions

In this work, we established the metalation of secondary diaminophosphine boranes with alkali metal amides as broadly applicable and robust access route to nucleophilic diaminophosphide building blocks based on both acyclic and N‐heterocyclic molecular frameworks. Hexamethyldisilazides emerged as reagents of choice because of their ability to react as universally applicable bases which allow free selection of alkali metals. Alternative approaches to the target molecules, such as metalation of the diaminophosphine boranes with organometallic reagents or reductive metalation of P‐chloro‐substituted precursors, worked well in selected cases but gave generally erratic results. Moreover, borane complexes of diazaphospholenes materialized as critical substrates, which reacted unselectively to afford low yields of spectroscopically detectable metalation products beside unidentified by‐products. We presume that this behavior reflects the pertinent hydride character of the P−H bonds in the free phosphines.

Beyond previous findings, sodium and potassium dimethylaminosphosphide boranes were for the first time isolated as room‐temperature‐stable solids. Their crystallographic characterization reveals the presence of salt‐like structures that are distinguished by intimate interactions between ions of opposite charge via a combination of dative M⋅⋅⋅N and M⋅⋅⋅P and agostic M⋅⋅⋅H−B contacts. While both types of interactions are not unprecedented for aryl and alkyl phosphide boranes, the availability of additional nitrogen donor centers increases the density of the interaction network.

Last, but not least, the potential of diaminophosphide boranes to act as synthetic building blocks is illustrated by their conversion into phosphine boranes that are distinguished by a combination of electronegative amino and electropositive functional silyl groups. Initial studies indicate that removal of the borane units is in principle feasible, and we are currently striving to develop reliable synthetic protocols to accomplish this task.

## Experimental Section

All reactions were carried out under an atmosphere of inert Argon and in flame‐dried glassware if not mentioned otherwise. Solvents were dried by distillation from alkali metals (THF, Et_2_O, toluene) or by using a solvent purification system (pentane, hexane, MeCN). NMR spectra were recorded on Bruker Avance 250 or Avance 400 instruments. Chemical shifts in ^1^H NMR spectra were referenced to TMS using the residual signals of the deuterated solvent (^1^H, *δ*([D_8_]THF)=1.73 ppm; *δ*(C_6_D_6_)=7.15 ppm) as secondary reference. NMR Spectra of heteronuclei were referenced using the *Ξ*‐scale^[31]^ with TMS (*Ξ*=25.145020 MHz, ^13^C; *Ξ*=19.867187 MHz, ^29^Si), BF_3_Et_2_O (*Ξ*=32.083974 MHz, ^11^B) and 85 % H_3_PO_4_ (*Ξ*=40.480747 MHz, ^31^P) as secondary references. Coupling constants involving boron refer to ^11^B isotopomers, with 1:1:1:1 multiplets being denoted as q. The product composition in metalation experiments was derived from analysis of the ^1^H NMR signals of the *N*‐substituents of **1 b**–**f**. Elemental analyses were carried out with an Elementar Micro Cube elemental analyzer. Mass spectra were obtained with a Bruker Daltonics Mikrotof‐Q‐Mass spectrometer. Phosphine boranes **2 a**,^[10]^ **2 e** ^[11]^ and **3 a** ^[9]^ were prepared as reported.

X‐ray diffraction studies were carried out using a Bruker Kappa Apex II diffractometer equipped with a Duo CCD‐detector and a KRYO‐FLEX cooling device with Mo‐$K_{\alpha }$ radiation (*λ*=0.71073 Å) at *T=*130(2) K. The structures were solved by direct methods (SHELXS‐97^[32]^) and refined with a full‐matrix least‐squares Scheme on *F* ^2^ (SHELXL‐2014 and SHELXL‐97^[33]^). Semi‐empirical absorption corrections were applied. Non‐hydrogen atoms except disordered carbon atoms were refined anisotropically, hydrogen atoms at boron in Na(THF)[**1 b**], K[**1 b**], isotropically, and all other hydrogen atoms with a riding model. The peripheral atoms in the SiMe_2_Cl group of **6 e** and five of 16 Et‐groups in **7** and two of four Et‐groups in **8** are each disordered over two positions. The carbon atoms in the disordered sections were refined isotropically and with restraints.

Deposition numbers 2002662, 2002663, 2002666, 2002665, and 2002664 (Na(THF)[**1 b**], K[**1 b**], **6 e**, **7**, **8**) contain the supplementary crystallographic data for this paper. These data are provided free of charge by the joint Cambridge Crystallographic Data Centre and Fachinformationszentrum Karlsruhe Access Structures service.

**Bis(dimethylamino)phosphine borane (2 b)**: Method A: PCl_3_ (0.1 mL 1.14 mmol) was added under stirring to a cooled (−78 °C) solution of P(NMe_2_)_3_ (0.42 mL 2.29 mmol) in Et_2_O (50 mL). Stirring was continued while the solution was allowed to warm to ambient temperature. The solution was cooled again to −78 °C and BH_3_⋅SMe_2_ (0.33 mL, 3.43 mmol) was added. The mixture was stirred for 1 h at −78 °C and allowed to warm to RT. After cooling once more to −78 °C, LiAlH_4_ (3.43 mL 1 m solution in THF, 3.43 mmol) was added. The mixture was stirred for 1 h at −78 °C, allowed to warm to RT, and stirred for further 24 h. Water (0.5 mL) was added and volatiles were evaporated under reduced pressure. The solid residue was extracted with hexane (20 mL). The extract was dried (Na_2_SO_4_) and solids were removed by filtration. Evaporation of the solvent under reduced pressure afforded **2 b** as colorless solid which melted around RT (yield 266 mg, 58 %). Method B: PCl_3_ (0.49 mL 5.6 mmol), P(NMe_2_)_3_ (2.00 mL 11.2 mmol) and BH_3_⋅SMe_2_ (1.59 mL, 16.8 mmol) were reacted in Et_2_O (30 mL) as described under A. Li[BEt_3_H] (16.8 mL 1 m solution in THF, 16.8 mmol) was added. The mixture was allowed to warm to RT and stirred for further 18 h. Volatiles were evaporated under reduced pressure and the residue was taken up in pentane (150 mL). The resulting mixture was filtered over Celite. Evaporation of the solvent under reduced pressure gave **2 b** as colorless oil (yield 1.137 g, 51 %), which contained according to NMR measurements 10 to 20 % dimethylamine borane. ^31^P{^1^H} NMR (C_6_D_6_): *δ*=89.2 ppm (q, ^1^ *J*_PB_=72 Hz); ^11^B{^1^H} NMR (C_6_D_6_): *δ*=−40.2 ppm (d, ^1^ *J*_PB_=73 Hz); ^1^H NMR (C_6_D_6_): *δ*=5.96 (d, ^1^ *J*_PH_=433 Hz, 1 H, PH), 2.27 (d, ^3^ *J*_PH_=9.7 Hz, 12 H, CH_3_N), 1.42 ppm (dm, ^1^ *J*_BH_=97 Hz, ^2^ *J*_PH_=16 Hz, 3 H, BH); ^13^C{^1^H} NMR (C_6_D_6_): *δ*=38.5 ppm (d, ^2^ *J*_PC_=2 Hz, CH_3_N); MS(ESI): m/z=119.08 [M^+^‐H, ‐BH_3_].

**Bis(diisopropylamino)phosphine borane (2 c)**: LiBH_4_ (0.53 mL 1 m solution in THF, 0.53 mmol) was added to a stirred solution of (*i*Pr_2_N)_2_PCl (0.50 g, 1.8 mmol) in Et_2_O (25 mL). The mixture was stirred for 1 h. Volatiles were evaporated under reduced pressure and the residue extracted with hexane (4×20 mL). The combined extracts were filtered, the filtrate concentrated under reduced pressure, and the product isolated by crystallization at −24 °C (yield 0.37 g, 77 %). ^31^P{^1^H} NMR (C_6_D_6_): *δ*=46.3 ppm (q, ^1^ *J*_PB_=72 Hz); ^11^B{^1^H} NMR (C_6_D_6_): *δ*=−35.6 ppm (d, ^1^ *J*_PB_=73 Hz); ^1^H NMR (C_6_D_6_): *δ*=6.70 (dq, ^1^ *J*_PH_=407 Hz, ^3^ *J*_HH_=5 Hz, 1 H, PH), 3.43 (dsept, ^3^ *J*_PH_=14 Hz, ^3^ *J*_HH_=7 Hz, 4 H, CHN), 1.61 (q, ^1^ *J*_BH_=107 Hz, 3 H, BH), 1.10 (d, ^3^ *J*_HH_=7 Hz, 12 H, CH_3_), 0.98 ppm (d, ^3^ *J*_HH_=7 Hz, 12 H, CH_3_); ^13^C{^1^H} NMR (C_6_D_6_): *δ*=46.4 ppm (d, ^2^ *J*_PC_=6 Hz, CHN), 22.6 (m, CH_3_); MS(ESI): m/z=246.2 [M^+^]; C_12_H_32_BN_2_P (246.19 g mol^−1^): calcd C 58.55 H 13.10 N 11.38, found C 58.28 H 12.84 N 11.13.

**1,3‐Bis(2,6‐xylyl)‐1,3,2‐diazaphospholidine borane (2 d)**: NaBH_4_ (84 mg, 1.9 mmol) was added under stirring to a cooled (0 °C) solution of 2‐chloro‐1,3‐bis(2,6‐xylyl)1,3,2‐diazaphospholidine (546 mg, 1.60 mmol) in MeCN (10 mL). The mixture was stirred for 24 h and during this time allowed to warm slowly to RT. Water (0.5 mL) was added, volatiles evaporated under reduced pressure and the residue extracted with toluene (75 mL). The extract was dried (Na_2_SO_4_) and filtered. Evaporation of the filtrated gave **2 d** as yellow solid (yield 512 mg, >99 %). ^31^P{^1^H} NMR (C_6_D_6_): *δ*=75.3 ppm (q, ^1^ *J*_PB_=67 Hz); ^11^B{^1^H} NMR (C_6_D_6_): *δ*=−39.8 ppm (d, ^1^ *J*_PB_=67 Hz); ^1^H NMR (C_6_D_6_): *δ*=7.35 (d, ^1^ *J*_PH_=347 Hz, 1 H, PH), 6.98 (d, ^3^ *J*_HH_=5 Hz, 4 H, *m*‐C_6_H_3_), 6.91 (m, 2 H, *p*‐C_6_H_3_), 3.21–2.88 (m, 4 H, CH_2_), 2.48 (s, 6 H, CH_3_), 2.10 (s, 6 H, CH_3_), 1.2 ppm (br q, ^1^ *J*_BH_=100 Hz, BH_3_); ^13^C{^1^H} NMR (C_6_D_6_): *δ*=139.3 (d, ^2^ *J*_PC_=3 Hz, CN), 138.0 (d, ^3^ *J*_PC_=2 Hz, *o*‐C), 129.1 (d, ^4^ *J*_PC_=1 Hz, *m*‐C), 128.6 (d, ^5^ *J*_PC_=1 Hz, *p*‐C), 49.1 (s, CH_2_), 18.7 (d, ^4^ *J*_PC_=1 Hz, CH_3_), 18.5 ppm (s, CH_3_); MS ((+)‐ESI): m/z=337.14 [MK^+^–BH_3_]; C_18_H_26_BN_2_P (312.19 g mol^−1^): calcd C 69.25 H 8.39 N 8.97, found C 69.00 H 8.51 N 8.68.

**1,3‐Bis(2,6‐xylyl)‐1,3,2‐diazaphospholene borane (2 f)**: NaBH_4_ (113 mg, 2.98 mmol) was added to a suspension of 2‐bromo‐1,3‐bis(2,6‐xylyl)‐1,3,2‐diazaphospholene (746 mg, 1.98 mmol) in MeCN (50 mL). The mixture was stirred for 18 h, filtered, and evaporated to dryness. The residue was washed with pentane and dried in vacuum to afford 477 mg (77 %) of crude **2 f**. Further purification was feasible by extracting the crude product with benzene, filtration, and evaporation of the filtrate to dryness. ^31^P{^1^H} NMR (C_6_D_6_): *δ*=79.8 ppm (q, ^1^ *J*_PB_=50 Hz); ^11^B{^1^H} NMR (C_6_D_6_): *δ*=−40.2 ppm (d, ^1^ *J*_PB_=54 Hz); ^1^H NMR (C_6_D_6_): *δ*=8.10 (d, ^1^ *J*_PH_=344 Hz, 1 H, PH), 7.01–6.82 (m, 6 H, m/*p*‐C_6_H_3_), 5.37 (d, ^3^ *J*_PH_=10 Hz, 2 H, CH), 2.43 (s, 6 H, CH_3_), 2.10 (s, 6 H, CH_3_), 1.6 ppm (br, BH_3_); ^13^C{^1^H} (C_6_D_6_): *δ*=138.7 (d, ^3^ *J*_PC_=3 Hz, *o*‐C), 137.4 (d, ^3^ *J*_PC_=2 Hz, 2 *o*‐C), 136.4 (d, ^2^ *J*_PC_=6 Hz, *i*‐C), 129.0 (s, *p*‐C), 128.4 (d, ^4^ *J*_PC_=1 Hz, *m*‐C), 127.8 (d, ^4^ *J*_PC_=1 Hz, *m*‐C), 118.8 (d, ^3^ *J*_PC_=1 Hz, NCH), 19.3 (s, CH_3_), 18.5 ppm (d, ^4^ *J*_PC_=1 Hz, CH_3_); MS ((+)‐ESI): m/z=310.19 [M^+^], 311.19 [M+H^+^]; C_18_H_24_BN_2_P (310.19 g mol^−1^): calcd C 69.70 H 7.80 N 9.03, found C 68.45 H 7.88 N 9.06. The reason for the low carbon content is not known.

**Chloro‐bis(dimethylamino)phosphine borane (3 b)**: BH_3_⋅SMe_2_ (1.3 mL, 13.6 mmol) was added under stirring to a cooled (0 °C) solution of (Et_2_N)_2_PCl (2 mL, 13.6 mmol) in Et_2_O (10 mL). After 30 min, the solution was allowed to warm to RT and stirred for further 18 h. The solvent was then removed under reduced pressure and the residue dissolved in hexane (10 mL). After reaction control by ^31^P NMR, enough BH_3_⋅SMe_2_ (1.3 mL, 13.6 mmol) to convert unreacted starting material and more hexane (10 mL) were added. Filtering the mixture over Celite and removal of the solvent under reduced pressure gave **3 b** as yellow oil (1.52 g, 66 %). The product is thermally unstable and decomposes even below room temperature; it must be used immediately and cannot be stored.^31^P{^1^H} NMR (C_6_D_6_): *δ*=139.7 ppm (q, ^1^ *J*_PB_=70 Hz); ^11^B{^1^H} NMR (C_6_D_6_): *δ*=−36.0 ppm (d, ^1^ *J*_PB_=67 Hz); ^1^H NMR (C_6_D_6_): *δ*=2.27 (d, ^*3*^ *J*_PH_=12 Hz, 12 H, CH_3_N), 1.59 ppm (dq, ^1^ *J*_BH_=99 Hz, ^2^ *J*_PH_=14 Hz, 3 H, BH); ^13^C{^1^H} (C_6_D_6_): *δ*=36.7 ppm (d, ^2^ *J*_PC_=4 Hz, CH_3_N). The thermal instability precluded obtaining a meaningful elemental analysis.

**General procedure for the metalation of secondary diaminophosphine boranes 2 b,d,f with alkali metal bis(trimethylsilyl)amides**: MHMDS (1.1 to 1.2 equiv., M=Li, Na, K) was added to a 0.06–0.2 m solution of the secondary diaminophosphine borane in THF or [D_8_]THF (1 to 2 mL). The solution was stirred for 10 min at RT. An aliquot of the reaction mixture was then transferred to an NMR tube and characterized by multinuclear NMR spectroscopy. Conversion to the metalation product was derived from evaluation of the integrals in ^1^H NMR spectra. Deprotonation at phosphorus was in all cases confirmed by the disappearance of the characteristic signal splitting due to ^1^ *J*_PH_.

Li[**1 b**]: Conversion 86 %. ^31^P{^1^H} NMR (C_6_D_6_): *δ*=145.9 ppm (q, ^1^ *J*_PB_=32 Hz); ^11^B{^1^H} NMR (C_6_D_6_): *δ*=−34.6 ppm (d, ^1^ *J*_PB_=32 Hz); ^1^H NMR ([D_8_]THF): *δ*=2.73 (d, ^3^ *J*_PH_=8 Hz, 12 H, CH_3_N), 0.64 ppm (broad q, ^1^ *J*_BH_=87 Hz, 3 H, BH); ^13^C{^1^H} NMR ([D_8_]THF): *δ*=43.8 ppm (broad s, CH_3_N).

Na[**1 b**]: Conversion 90 %. ^31^P{^1^H} NMR (C_6_D_6_): *δ*=145.0 ppm (q, ^1^ *J*_PB_=49 Hz); ^11^B{^1^H} NMR (C_6_D_6_): *δ*=−34.3 ppm (d, ^1^ *J*_PB_=49 Hz); ^1^H NMR ([D_8_]THF): *δ*=2.76 (d, ^3^ *J*_PH_=8 Hz, 12 H, CH_3_N), 0.66 ppm (dq, ^1^ *J*_BH_=87 Hz, ^2^ *J*_PH_=8.7 Hz, 3 H, BH); ^13^C{^1^H} NMR ([D_8_]THF): *δ*=44.9 ppm (broad s, CH_3_N).

K[**1 b**]: Conversion 84 %. ^31^P{^1^H} NMR (C_6_D_6_): *δ*=142.9 ppm (q, ^1^ *J*_PB_=49 Hz); ^11^B{^1^H} NMR (C_6_D_6_): *δ*=−32.9 ppm (d, ^1^ *J*_PB_=49 Hz); ^1^H NMR ([D_8_]THF): *δ*=2.7 (d, ^3^ *J*_PH_=6 Hz, 12 H, CH_3_N), 0.70 ppm (broad q, ^1^ *J*_BH_=86 Hz, 3 H, BH); ^13^C{^1^H} NMR ([D_8_]THF): *δ*=44.3 ppm (broad s, CH_3_N).

K[**1 f**]: conversion 70 %. ^31^P{^1^H} NMR (C_6_D_6_): *δ*=201.6 ppm (q, ^1^ *J*_PB_=24 Hz); ^11^B{^1^H} NMR (C_6_D_6_): *δ*=−29.5 ppm (d, ^1^ *J*_PB_=24 Hz); ^1^H NMR ([D_8_]THF): *δ*=7.00–6.63 (m, 6 H, *m*,*p*‐C_6_H_3_), 5.54 (s, 2 H, NCH), 2.46 ppm (s, 12 H, CH_3_); ^13^C{^1^H} NMR ([D_8_]THF): *δ*=127.6 (s, *o*‐C_6_H_3_), 121.9 (s, *p*‐C_6_H_3_), 127.8 (d, ^4^ *J*_PC_=1 Hz, *m*‐C_6_H_3_), 117.6 (d, ^3^ *J*_PC_=6 Hz, NCH), 19.5 ppm (s, CH_3_).

**Isolation of sodium bis(dimethylamino)phosphide borane Na[1 b]**: A solution of Na[**1 b**] was prepared as described above from NaHMDS (80 mg, 0.43 mmol) and **2 b** (49 mg, 0.36 mmol) in THF (2 mL). Storage at −24 °C produced colorless, highly air and moisture sensitive crystals, which were separated by decantation (no yield determined) and characterized by NMR spectroscopy (see Table 2 and above) and a single‐crystal X‐ray diffraction study. The high chemical sensitivity precluded obtaining a satisfactory elemental analysis.

**Isolation of potassium bis(dimethylamino)phosphine borane K[1 b]**: KHMDS (133 mg, 0.66 mmol) and dibenzo‐18‐crown‐6 (216 mg, 0.60 mmol) were added to a solution of **2 b** (81 mg, 0.60 mmol) in a mixture of Et_2_O (3 mL) and THF (7 mL) was carried out as described above. The mixture was stirred for 5 min. Storage at −24 °C produced colorless, highly air and moisture sensitive crystals, which were separated by decantation (no yield determined) and characterized by NMR spectroscopy (see Table 2 and above) and a single‐crystal X‐ray diffraction study. The high chemical sensitivity precluded obtaining a satisfactory elemental analysis.

**Metalation of 2 b with MeMgCl**: A 3 m solution of MeMgCl in THF (60 μL, 0.18 mmol) was evaporated to dryness. A solution of **2 b** (20 mg, 0.15 mmol) in [D_8_]THF (1 mL) was added. The solution was stirred for 5 min and then analyzed by multinuclear NMR spectroscopy (see also Table 2). ^31^P{^1^H} NMR (C_6_D_6_): *δ*=142.4 ppm (q, ^1^ *J*_PB_=49 Hz); ^11^B{^1^H} NMR (C_6_D_6_): *δ*=−33.0 ppm (d, ^1^ *J*_PB_=49 Hz); ^1^H NMR ([D_8_]THF): *δ*=2.58 (d, ^3^ *J*_PH_=8 Hz, 12 H, NCH_3_), 0.45 ppm (broad q, ^1^ *J*_BH_=89 Hz, 3 H, BH); ^13^C{^1^H} NMR ([D_8_]THF): *δ*=43.4 ppm (d, ^2^ *J*_PC_=5 Hz, NCH_3_).

**General procedure for the metalation of secondary diaminophosphine boranes 2 c**,**e**: The metalation agent (for Li[**1 c**]: 1.6 equiv. 2.5 m *n*BuLi in hexane; for K[**1 c**] and K[**1 e**]: 1.2 equiv. KHMDS; for [K(dibenzo‐18‐ crown‐6)][**1 c**]: 1.2 equiv. KHMDS and 1.0 equiv. dibenzo‐18‐crown‐6; for Li[**1 e**]: 1.33 equiv. LDA; for [K(dibenzo‐18‐crown‐6)][**1 e**]: 2.0 equiv. KHMDS and 1.0 equiv. dibenzo‐18‐crown‐6) was added to a 3–6 mm solution of the secondary diaminophosphine borane in Et_2_O (20 to 25 mL). After stirring for 1 h, volatiles were removed under reduced pressure. The residue was dissolved in [D_8_]THF or C_6_D_6_ and characterized by multinuclear NMR spectroscopy. ^31^P and ^11^B NMR data are included in Table 2; additional data are given below. Conversion to the metalation product was derived from evaluation of the integrals in ^31^P{^1^H} NMR spectra. Deprotonation at phosphorus was in all cases confirmed by the disappearance of the characteristic signal splitting due to ^1^ *J*_PH_.

Li[**1 c**]: Conversion >98 %. ^31^P{^1^H} NMR (C_6_D_6_): *δ*=61.8 ppm (q, ^1^ *J*_PB_=62 Hz); ^11^B{^1^H} NMR (C_6_D_6_): *δ*=−32.9 ppm (d, ^1^ *J*_PB_=62 Hz); ^1^H NMR ([D_8_]THF): *δ*=3.56 (sept, ^3^ *J*_HH_=7 Hz, 4 H, NCH), 1.12 (d, ^3^ *J*_HH_=7 Hz, 12 H, CH_3_), 1.02 (d, ^3^ *J*_HH_=7 Hz, 12 H, CH_3_), 0.33 ppm (q, ^1^ *J*_BH_=83 Hz, 3 H, BH); ^13^C{^1^H} NMR ([D_8_]THF): *δ*=47.3 (broad s, NCH), 23.9 ppm (broad s, CH_3_; ^7^Li NMR ([D_8_]THF): *δ*=−0.39 ppm (s).

K[**1 c**]: ^31^P{^1^H} NMR (C_6_D_6_): *δ*=53.4 ppm (q, ^1^ *J*_PB_=64 Hz); ^11^B{^1^H} NMR (C_6_D_6_): *δ*=−29.5 ppm (d, ^1^ *J*_PB_=64 Hz); ^1^H NMR (C_6_D_6_): *δ*=3.75 (sept, ^3^ *J*_HH_=7 Hz, 4 H, NCH), 1.43 (d, ^3^ *J*_HH_=7 Hz, 12 H, CH_3_), 1.23 (d, ^3^ *J*_HH_=7 Hz, 12 H, CH_3_), 0.47 ppm (q, ^1^ *J*_BH_=84 Hz, 3 H, BH); ^13^C{^1^H} NMR (C_6_D_6_): *δ*=47.9 (broad s, NCH), 24.7 (d, ^3^ *J*_PC_=7 Hz, CH_3_), 24.2 ppm (d, ^3^ *J*_PC_=5 Hz, CH_3_).

[K(dibenzo‐18‐crown‐6)][**1 c**]: Conversion 94 %. ^31^P{^1^H} NMR (C_6_D_6_): *δ*=68.5 ppm (q, ^1^ *J*_PB_=53 Hz); ^11^B{^1^H} NMR (C_6_D_6_): *δ*=−30.4 ppm (d, ^1^ *J*_PB_=53 Hz); ^1^H NMR ([D_8_]THF): *δ*=6.97–6.84 (m, 8 H, C_6_H_4_), 4.22–4.14 (m, 16 H, OCH_2_), 3.61 (sept, ^3^ *J*_HH_=7 Hz, 4 H, CH), 1.13 (d, ^3^ *J*_HH_=7 Hz, 12 H, CH_3_), 1.04 (d, ^3^ *J*_HH_=7 Hz, 12 H, CH_3_), 0.76 ppm (dq, ^1^ *J*_BH_=85 Hz, ^2^ *J*_PH_=9 Hz, 3 H, BH_3_); ^13^C{^1^H} NMR ([D_8_]THF): *δ*=120.7 (s, C_6_H_4_), 110.7 (s, C_6_H_4_), 68.6 (s, OCH_2_), 67.3 (s, OCH_2_), 47.3 (d, ^2^ *J*_PC_=5 Hz, NCH), 24.1 ppm (d, ^3^ *J*_PC_=1 Hz, CH_3_).

Li[**1 e**]: ^31^P{^1^H} NMR (C_6_D_6_): *δ*=185.7 ppm (q, ^1^ *J*_PB_=29 Hz); ^11^B{^1^H} NMR (C_6_D_6_): *δ*=−32.2 ppm (d, ^1^ *J*_PB_=29 Hz); ^1^H NMR ([D_8_]THF): *δ*=6.97 (broad, 6 H, C_6_H_3_), 4.1 (broad, 4 H, CH), 3.99 (broad, 2 H, NCH_2_), 3.26 (broad, 2 H, NCH_2_), 1.22 (br d, ^3^ *J*_HH_=7 Hz, 12 H, CH_3_), 1.18 ppm (br d, ^3^ *J*_HH_=7 Hz, 12 H, CH_3_); ^13^C{^1^H} NMR ([D_8_]THF): *δ*=150.6 (broad s, *o*‐C), 149.1 (s, *o*‐C), 143.1 (d, ^2^ *J*_PC_=11 Hz, *i*‐C), 124.1 (d, ^3^ *J*_PC_=2 Hz, *p*‐C), 122.9 (br, *m*‐C), 54.2 (d, ^2^ *J*_PC_=7 Hz, NCH_2_), 27.4 (broad s, CH), 25.0–24.5 ppm (broad s, CH_3_).

K[**1 e**]: ^31^P{^1^H} NMR (C_6_D_6_): *δ*=187.7 ppm (q, ^1^ *J*_PB_=26 Hz); ^11^B{^1^H} NMR (C_6_D_6_): *δ*=−29.9 ppm (d, ^1^ *J*_PB_=26 Hz); ^1^H NMR ([D_8_]THF): *δ*=6.94 (broad, 6 H, C_6_H_3_), 4.22–3.80 (m, 6 H, CHMe_2_ and NCH_2_), 3.16 (m, 2 H, NCH_2_), 1.25–1.12 ppm (m, 24 H, CH_3_); ^13^C{^1^H} NMR ([D_8_]THF): *δ*=150.5 (s, *o*‐C_6_H_3_), 149.4 (s, *o*‐C_6_H_3_), 143.3 (d, ^2^ *J*_PC_=10 Hz, *i*‐C_6_H_3_), 124.3 (s, *p*‐C_6_H_3_), 123.1 (broad, *m*‐C_6_H_3_), 122.7 (broad, *m*‐C_6_H_3_), 54.4 (d, ^2^ *J*_PC_=7 Hz, NCH_2_), 27.3 (broad, CH), 25.5 (s, CH_3_), 25.2 (s, CH_3_), 24.1 (s, CH_3_), 23.1 ppm (s, CH_3_).

[K(dibenzo‐18‐crown‐6)][**1 e**]: ^31^P{^1^H} NMR (C_6_D_6_): *δ*=197.3 ppm (q, ^1^ *J*_PB_=23 Hz); ^11^B{^1^H} NMR (C_6_D_6_): *δ*=−29.8 ppm (d, ^1^ *J*_PB_=23 Hz); ^1^H NMR ([D_8_]THF): *δ*=6.95 (m, 6 H, C_6_H_3_), 6.82 (broad, 8 H, C_6_H_4_), 4.27–4.04 (m, 6 H, CHMe_2_ and NCH_2_),3.98 (m, 8 H, OCH_2_), 3.84 (m, 8 H, OCH_2_), 3.22 (m, 2 H, NCH_2_), 1.20 (d, ^3^ *J*_HH_=7 Hz, 12 H, CH_3_), 1.15 (d, ^3^ *J*_HH_=7 Hz, 12 H, CH_3_), 0.67 ppm (broad q, ^1^ *J*_BH_=83 Hz, 3 H, BH_3_); ^13^C{^1^H} NMR ([D_8_]THF): *δ*=142.2 (d, ^2^ *J*_PC_=10 Hz, *i*‐C_6_H_3_), 122.1 (s, *m*‐C_6_H_3_), 118.8 (s, C_6_H_4_), 108.8 (s, C_6_H_4_), 66.6 (s, OCH_2_), 65.3 (s, OCH_2_), 52.6 (d, ^2^ *J*_PC_=7 Hz, NCH_2_), 25.6 (broad, CH, 23.1 (broad, CH_3_), 22.9 (broad, CH_3_), 22.7 ppm (broad, CH_3_).

**General procedure for the metalation of diaminochlorophosphine boranes 3 a,b**: The reductant (3 to 5 equiv. of alkali metal or 2.2 equiv. of alkali metal naphthalenides) was added to a solution of diaminochlorophosphine borane (0.1–0.2 m) in THF. The solution was agitated for 1–18 h at RT and then subjected to NMR spectroscopic analysis (see Table 2 for results).

**Reactions of K[1 a,c,e] with 1,2‐dichlorodisilane 5**: Solutions of potassium diaminophosphide boranes K[**1 a,c,e**] were prepared as described above from the phosphine boranes (**2 a**: 82 mg, 0.43 mmol; **2 c**: (90 mg, 0.36 mmol; **2 e**: 101 mg, 0.23 mmol) and KHMDS (1.2 equiv.) in Et_2_O (10–25 mL). Disilane **5** (for K[**1 a**]: 40 μL, 0.21 mmol, 0.5 equiv.; for K[**1 c**]: 70 μL, 0.36 mmol, 1 equiv.; for K[**1 e**]: 22 μL, 0.23 mmol, 1 equiv.) was added by means of a microliter syringe. The mixture was stirred for 12–18 h. The solvent was evaporated under reduced pressure and the residue extracted with hexane (10–20 mL). The extracts were filtered through Celite. Disilanes **6 e**, **7** separated as colorless, crystalline products upon concentration of the extracts to a volume of 10 mL and storage at −24 °C (**6 e**: yield 62 mg, 44 %; **7**: yield 40 mg, 38 %). Crude disilane **6 c** was obtained as colorless oil after evaporation of volatiles (yield 112 mg, 71 %).

**6 c**: ^31^P{^1^H} NMR (C_6_D_6_): *δ*=94.3 ppm (q, ^1^ *J*_PB_=70 Hz); ^11^B{^1^H} NMR (C_6_D_6_): *δ*=−32.2 ppm (d, ^1^ *J*_PB_=70 Hz); ^1^H NMR (C_6_D_6_): *δ*=3.70 (dsept, ^3^ *J*_PH_=12 Hz, ^3^ *J*_HH_=7 Hz, 4 H, NCH), 1.23 (d, ^3^ *J*_HH_=7 Hz, 12 H, CH_3_), 1.18 (d, ^3^ *J*_HH_=7 Hz, 12 H, CH_3_), 0.66 (s, 6 H, SiCH_3_), 0.56 ppm (d, ^3^ *J*_PH_=7 Hz, 6 H, SiCH_3_); ^13^C{^1^H} NMR (C_6_D_6_): *δ*=51.4 (d, ^2^ *J*_PC_=3 Hz, NCH), 24.8 (d, ^3^ *J*_PC_=3 Hz, CH_3_), 24.7 (d, ^3^ *J*_PC_=3 Hz, CH_3_), 3.9 (d, ^2^ *J*_PC_=2 Hz, PSiCH_3_), −2.5 ppm (d, ^3^ *J*_PC_=7 Hz, ClSiCH_3_); ^29^Si‐DEPT (C_6_D_6_): *δ*=25.2 (d, ^2^ *J*_PSi_=22 Hz, SiCl), −20.4 ppm (d, ^1^ *J*_PSi_=77 Hz, SiP).

**6 e**: ^31^P{^1^H} NMR (C_6_D_6_): *δ*=119.6 ppm (broad); ^11^B{^1^H} NMR (C_6_D_6_): *δ*=−33.5 ppm (broad); ^1^H NMR (C_6_D_6_): *δ*=7.18–7.16 (m, 4 H, *m*‐C_6_H_3_), 7.05 (m, 2 H, *p*‐C_6_H_3_), 4.03 (sept, ^3^ *J*_HH_=7 Hz, 2 H, CH), 3.46 (sept, ^3^ *J*_HH_=7 Hz, 2 H, CH), 3.44 (m, 2 H, NCH_2_), 3.27 44 (m, 2 H, NCH_2_), 1.58 (d, ^3^ *J*_HH_=7 Hz, 6 H, CH_3_), 1.37 (d, ^3^ *J*_HH_=7 Hz, 6 H, CH_3_), 1.16 (d, ^3^ *J*_HH_=7 Hz, 6 H, CH_3_), 1.13 (d, ^3^ *J*_HH_=7 Hz, 6 H, CH_3_), 0.51 (d, ^3^ *J*_PH_=6 Hz, 6 H, SiCH_3_), 0.30 ppm (s, 6 H, SiCH_3_); ^13^C{^1^H} NMR (C_6_D_6_): *δ*=150.4 (d, ^3^ *J*_PC_=0.5 Hz, *o*‐C), 148.7 (d, 3JPC=3 Hz, *o*‐C), 137.1 (d, 2JPC=6 Hz, *i*‐C), 128.3 (d, ^4^ *J*_PC_=1 Hz, *m*‐C), 124.7 (d, ^4^ *J*_PC_=1 Hz, *m*‐C), 123.8 (s, *p*‐C), 53.6 (d, *J*_PC_=2 Hz, NCH_2_), 28.9 (s, CH), 28.6 (s, CH), 27.2 (s, CH_3_), 27.0 (s, CH_3_), 23.3 (s, CH_3_), 23.2 (s, CH_3_), 3.2 (d, ^3^ *J*_PC_=2 Hz, SiCH_3_), −2.72 ppm (d, ^2^ *J*_PC_=4 Hz, SiCH_3_); ^29^Si‐DEPT (C_6_D_6_): *δ*=24.2 ppm (d, ^2^ *J*_PSi_=23 Hz, SiCl); C_30_H_50_BClN_2_PSi_2_ (572.14 g mol^−1^): calcd C 62.98 H 8.81 N 4.90, found C 62.62 H 9.39 N 4.81.

**7**: ^31^P{^1^H} NMR (C_6_D_6_): *δ*=89.9 ppm (broad d, 77 Hz); ^11^B{^1^H} NMR (C_6_D_6_): *δ*=−36.4 ppm (d, ^1^ *J*_PB_=56 Hz); ^1^H NMR (C_6_D_6_): *δ*=3.14–3.02 (m, 16 H, CH_2_), 0.96 (t, ^3^ *J*_HH_=7 Hz, 24 H, CH_3_), 0.80 ppm (m, 12 H, SiCH_3_); ^13^C{^1^H} NMR (C_6_D_6_): *δ*=42.3 (broad, NCH_2_), 14.0 (t, 2 Hz, CH_3_), −1.1 ppm (d, 5 Hz, SiCH_3_); ^29^Si‐DEPT NMR (C_6_D_6_): *δ*=−19.0 ppm (d, 82 Hz); C_20_H_58_B_2_N_4_P_2_Si_2_ (494.45 g mol^−1^): calcd C 48.58 H 11.82 N 11.33, found C 48.33 H 11.78 N 10.96.

**Potassium bis(diethylamino)phosphino‐dithioformate borane semi‐hydrate 8**: A solution of K[**1 a**] was prepared as described above from **2 a** (203 mg, 1.06 mmol), KHMDS (256 mg, 1.28 mmol) and Et_2_O (15 mL). CS_2_ (60 μL, 1.06 mmol) was added through a microliter syringe. The solution was stirred for 18 h. The solvent was evaporated under reduced pressure. The remaining work‐up was carried out in air. The residue extracted with Et_2_O (10 mL). The extract was filtered in air over Celite, and pentane (10 mL) was added. Storing the solution at −24 °C afforded **8** as red crystalline solid (yield 211 mg, 60 %). ^31^P{^1^H} NMR (C_6_D_6_): *δ*=93.4 ppm (broad); ^11^B{^1^H} NMR *δ*=−34.0 ppm (broad); ^1^H NMR (C_6_D_6_): *δ*=3.35 (m, ^2^ *J*_HH_=−14.4 Hz, ^3^ *J*_HH_=7.0 Hz, ^3^ *J*_PH_=10.1 Hz, 4 H, NCH_2_), 3.20 (m, ^2^ *J*_HH_=−14.4 Hz, ^3^ *J*_HH_=7.0 Hz, ^3^ *J*_PH_=9.6 Hz, 4 H, NCH_2_), 1.19 (t, ^3^ *J*_HH_=7.0 Hz, 12 H, CH_3_), 1.07 ppm (broad, 3 H, BH_3_); ^13^C{^1^H} NMR (C_6_D_6_): *δ*=42.0 (broad), 14.4 (d, ^3^ *J*_PC_=3 Hz, CH_3_), detection of the CS_2_‐carbon atom was precluded by low S/N; C_9_H_22_BKN_2_PS_2_⋅0.5 H_2_O (313.3 g mol^−1^): calcd C 34.50 H 7.72 N 8.94 S 20.47; found C 33.85 H 7.49 N 8.67 S 19.93. The deviation between calculated and found analytical data is presumably due to a higher water content of the hygroscopic sample (calculated for C_9_H_22_BKN_2_PS_2_⋅H_2_O: C 33.64 H 7.53 N 8.72 S 19.96); MS ((−)‐ESI): m/z=264.11 [(*M*−K)^−^].

## Conflict of interest

The authors declare no conflict of interest.

## Supporting information

As a service to our authors and readers, this journal provides supporting information supplied by the authors. Such materials are peer reviewed and may be re‐organized for online delivery, but are not copy‐edited or typeset. Technical support issues arising from supporting information (other than missing files) should be addressed to the authors.

Supplementary
