# Supplementary material for: Isolable Diaminophosphide Boranes
Source: Chemistry. 2020 Oct 19;26(66):15190–9. doi: 10.1002/chem.202002296 (PMC7756230; doi:10.1002/chem.202002296)
Supplement: Supplementary file 1 — Supplementary [file CHEM-26-15190-s001.pdf]

Chemistry–A European Journal

Supporting Information

Isolable Diaminophosphide Boranes

Markus Blum,[a] Tobias Dunaj,[a] Julius A. Knöller,[a] Christoph M. Feil,[a] Martin Nieger,[b] and Dietrich Gudat*[a]

Table of contents

Crystallography

Table S1 Crystallographic data for Na(THF)[**1b**], K[**1b**], **6e**, **7**, **8**·0.5 H_2O,

Figure S1 Graphical representation of the packing in crystalline K[**1b**].

Figure S2 Graphical representation of the second molecule in crystalline **6e**.

Figure S3 Graphical representation of the second, centrosymmetric molecule in crystalline **7**.

Figure S4 Graphical representation of the asymmetric unit of crystalline **8**.

NMR Spectra

Figure S5 – S65 Graphical representation of NMR spectra.

Table S1 Crystallographic data for Na(THF)[**1b**], K[**1b**], **6e**, **7**, **8**·0.5 H_2O

	Na(THF)[**1b**]	K[**1b**]	**6e**
CCDC	2002662	2002663	2002666
Empirical formula	$C_8H_{23}BN_2NaOP$	$C_8H_{30}B_2K_2N_4P_2$	$C_{30}H_{53}BClN_2PSi_2$
Formula weight/g mol^{-1}	228.05	344.12	575.15
T/K	130(2)	130(2)	130(2)
Wavelength/Å	0.71073	0.71073	0.71073
Crystal system	Triclinic	Monoclinic	Monoclinic
Space group	$P\bar{1}$	$P2_1/c$	$P2_1/c$
a/Å	6.5164(3)	13.6145(14)	15.2996(16)
b/Å	9.6363(5)	6.5681(9)	20.752(2)
c/Å	10.7572(6)	21.682(3)	22.267(2)
α/°	84.200(3)	90	90
β/°	81.077(3)	91.773(6)	100.832(5)
γ/°	83.606(2)	90	90
V/Å^{-3}	660.74(6)	1937.9(4)	6943.8(12)
Z	2	4	8
ρ_{calcd}/Mg m^{-3}	1.146	1.179	1.100
Absorption coeff. /mm^{-1}	0.215	0.644	0.246
F(000)	248	736	2496
Crystal size/mm^3	0.798 x 0.172 x 0.168	0.234 x 0.152 x 0.048	0.588 x 0.274 x 0.269
Θ-range for data collection/°	1.923 to 28.269°	1.879 to 28.458	1.673 to 28.458
Index ranges	-7 ≤ h ≤ 8, -12 ≤ k ≤ 12, -14 ≤ l ≤ 13	-18 ≤ h ≤ 12, -8 ≤ k ≤ 8, -29 ≤ l ≤ 25	-20 ≤ h ≤ 20, -27 ≤ k ≤ 27, -10 ≤ l ≤ 29
Refl. collected	11273	18035	128431
Independent refl.	3149 [R(int) = 0.0203]	4830 [R(int) = 0.0948]	17406 [R(int) = 0.0580]
Completeness to θ = 25.242°	98.0 %	99.8 %	100.0 %
Abs. correction	Semi-empirical from equivalents	Semi-empirical from equivalents	Semi-empirical from equivalents
Max. and min. transmission	0.7457 and 0.7081	0.7457 and 0.6896	0.7350 and 0.5991
Refinement method	Full-matrix least-squares on F^2	Full-matrix least-squares on F^2	Full-matrix least-squares on F^2
Data / restraints / parameters	3149 / 0 / 148	4830 / 0 / 213	17406 / 49 / 673
G.o.f. on F^2	1.037	0.951	1.024
Final R indices [I > 2σ(*I*)]	R1 = 0.0316 wR2 = 0.0719	R1 = 0.0499 wR2 = 0.0740	R1 = 0.0928 wR2 = 0.2893
R indices (all data)	R1 = 0.0445 wR2 = 0.0782	R1 = 0.1244 wR2 = 0.0899	R1 = 0.1040 wR2 = 0.3215
Extinction coeff.	n/a	n/a	n/a
Largest diff. peak and hole/e Å^{-3}	0.394 and -0.265	0.383 and -0.370	1.000 and -0.547
Abs. structure parameter	n/a	n/a	n/a

Table S1, continued

	7	8
CCDC	2002665	2002664
Empirical formula	$C_{20}H_{58}B_2N_4P_2Si_2$	$C_{18}H_{48}B_2K_2N_4OP_2S_4$
Formula weight/g mol^{-1}	494.44	626.60
T/K	130(2)	130(2)
Wavelength/Å	0.71073	0.71073
Crystal system	Monoclinic	Monoclinic
Space group	*C*2/c	*C*2/c
a/Å	30.485(2)	15.2110(15)
b/Å	14.8390(8)	6.9623(7)
c/Å	24.1873(16)	31.889(3)
α/°	90	90
β/°	120.514(2)	96.189(5)
γ/°	90	90
V/Å^{-3}	9426.2(10)	3357.5(6)
Z	12	4
ρ_{calcd}/Mg m^{-3}	1.045	1.240
Absorption coeff. /mm^{-1}	0.229	0.644
F(000)	3288	1336
Crystal size/mm^3	0.395 x 0.114 x 0.099	0.302 x 0.301 x 0.180
Θ-range for data collection/°	1.551 to 26.422	2.570 to 28.404
Index ranges	-38<=h<=38, -18<=k<=17, -30<=l<=30	-20<=h<=20, -9<=k<=9, -42<=l<=20
Refl. collected	38170	14809
Independent refl.	9670 [R(int) = 0.0440]	4176 [R(int) = 0.0507]
Completeness to θ = 25.242°	100.0 %	99.8 %
Abs. correction	Semi-empirical from equivalents	Semi-empirical from equivalents
Max. and min. transmission	0.7454 and 0.7047	0.7457 and 0.5959
Refinement method	Full-matrix least-squares on F^2	Full-matrix least-squares on F^2
Data / restraints / parameters	9670 / 432 / 423	4176 / 47 / 155
G.o.f. on F^2	1.015	1.170
Final R indices [I > 2σ(*I*)]	R1 = 0.0488<br>wR2 = 0.1140	R1 = 0.0778<br>wR2 = 0.1442
R indices (all data)	R1 = 0.0853<br>wR2 = 0.1326	R1 = 0.0988<br>wR2 = 0.1508
Extinction coeff.	n/a	n/a
Largest diff. peak and hole/e Å^{-3}	0.916 and -0.428	0.484 and -0.662
Abs. structure parameter	n/a	n/a

Figure S1. Graphical representation of the packing in crystalline K[**1b**]. For clarity, Hydrogen atoms except those of BH_3 groups were omitted, and the carbon atoms were drawn using a wire model. Thermal ellipsoids are drawn at the 50% probability level.

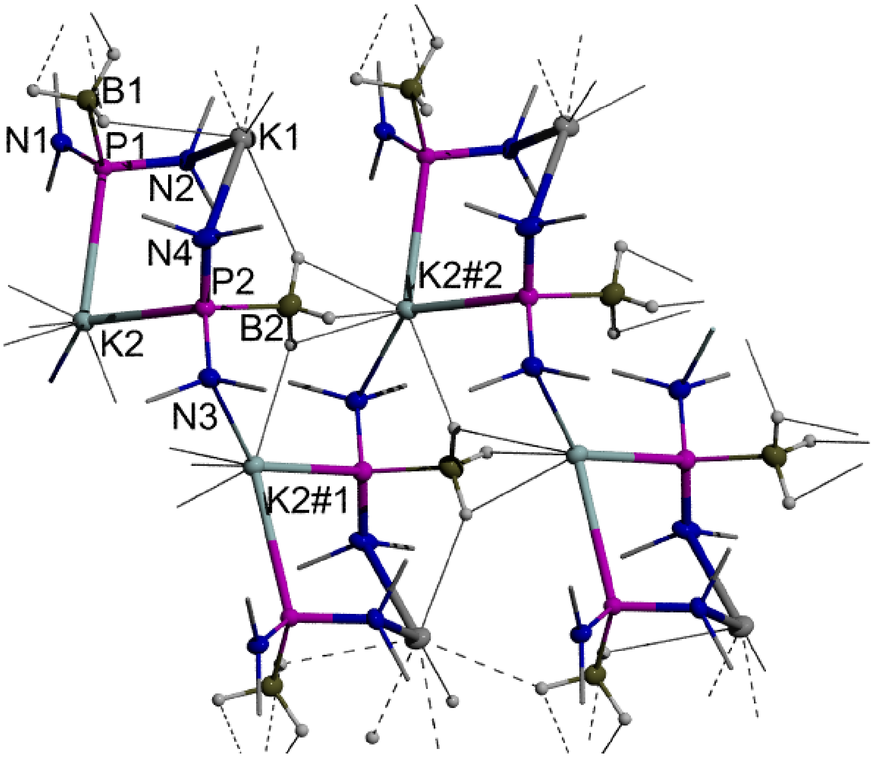

Figure S2. Graphical representation of the second molecule in crystalline **6e**. For clarity, hydrogen atoms except those of BH_3 groups were omitted and carbon atoms in the N-Dipp substituents represented using a wire model. The molecules shown illustrate the disorder of the $SiMe_2Cl$ group over two positions (occupancy factors 0.35 : 0.65). Thermal ellipsoids of heavy atoms were drawn at the 50% probability level. Selected distances (in Å) and torsional angles (in °): P1-N2 1.677(7), P1-N1 1.701(6), P1-B1 1.941(9), P1-Si1 2.289(3), Si1-Si2 2.356(3), Si2-Cl1A 2.055(4), Si2-Cl1B 2.069(6), P1-Si1-Si2-Cl1A -157.3(2), P1-Si1-Si2-Cl1B 96.9(3).

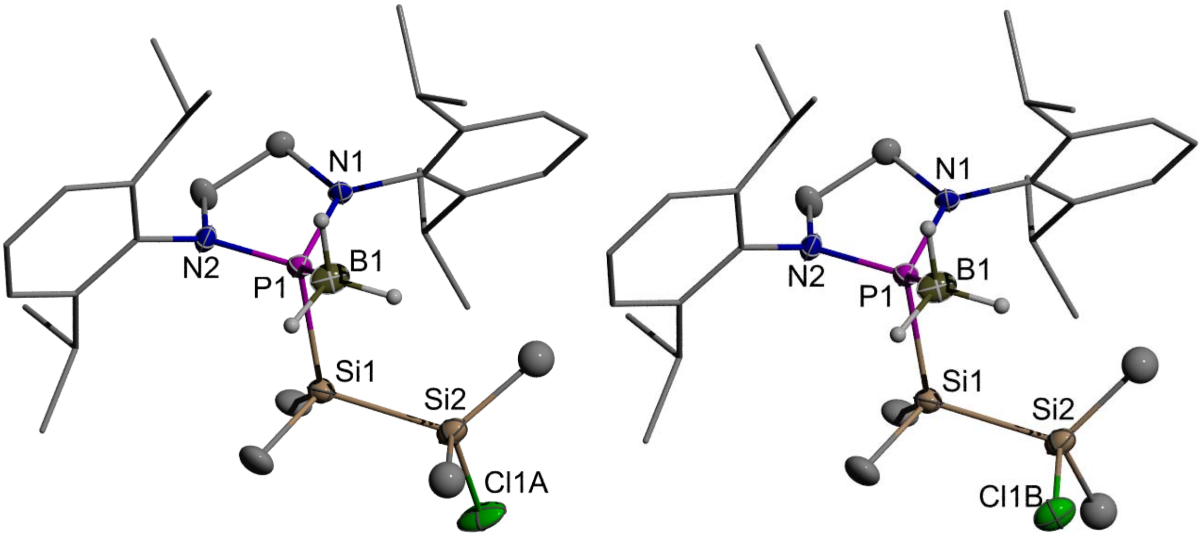

Figure S3. Graphical representation of the second, centrosymmetric molecule in crystalline **7**. For clarity, hydrogen atoms except those of BH_3 groups were omitted, and only one of two disordered orientations of the disordered Et-groups at N5 were drawn. Thermal ellipsoids of heavy atoms were drawn at the 50% probability level. Selected distances (in Å) and torsional angles (in °): P3-N5 1.668(2), P3-N6 1.668(2), P3-B3 1.929(3), P3-Si3 2.2941(10), Si3-Si3#1 2.3657(13), P3-Si3-Si3#1-P3#1 180°.

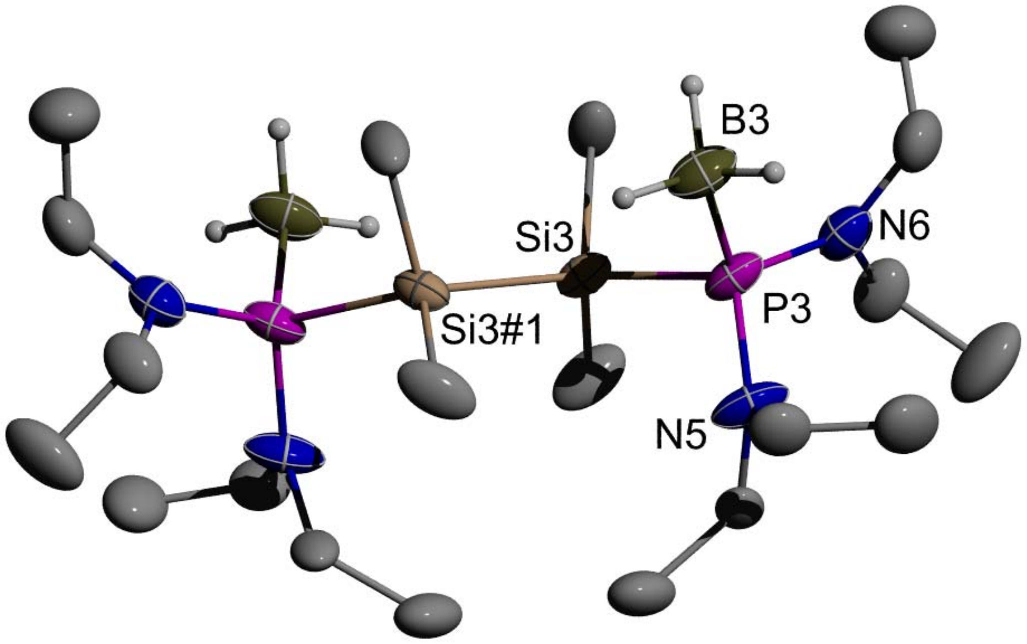

Figure S4. Graphical representation of the asymmetric unit of crystalline **8**. Hydrogen atoms except those of BH_3 groups were omitted, and only one of two orientations of the Et-groups on N2 was drawn. Thermal ellipsoids of heavy atoms were drawn at the 50% probability level.

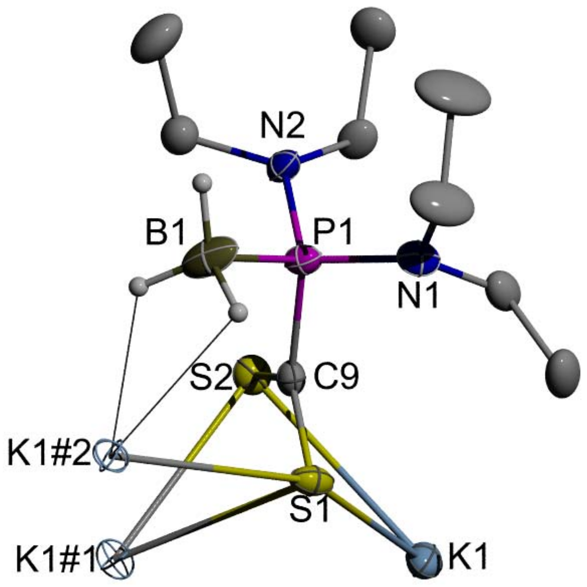

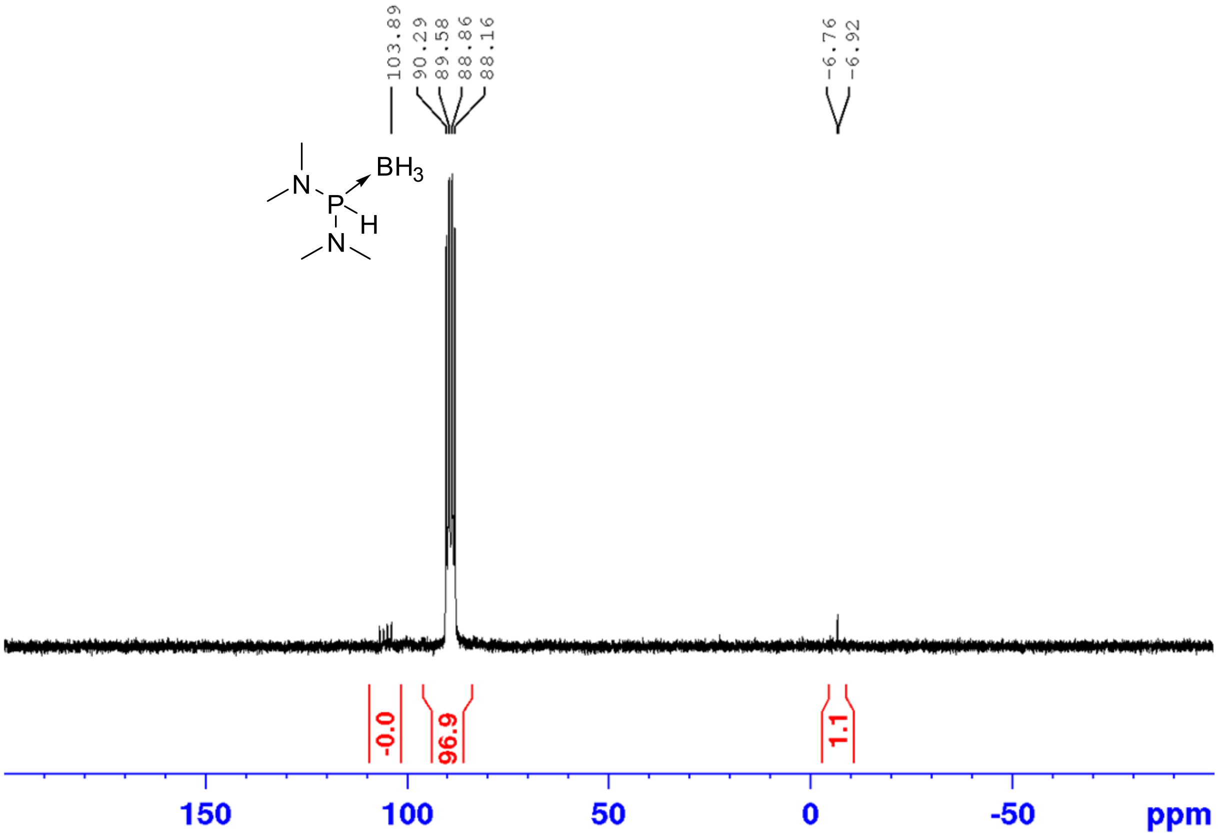

Figure S5: $^{31}P\{^{1}H\}$ NMR spectrum (C_6D_6) of **2b**.

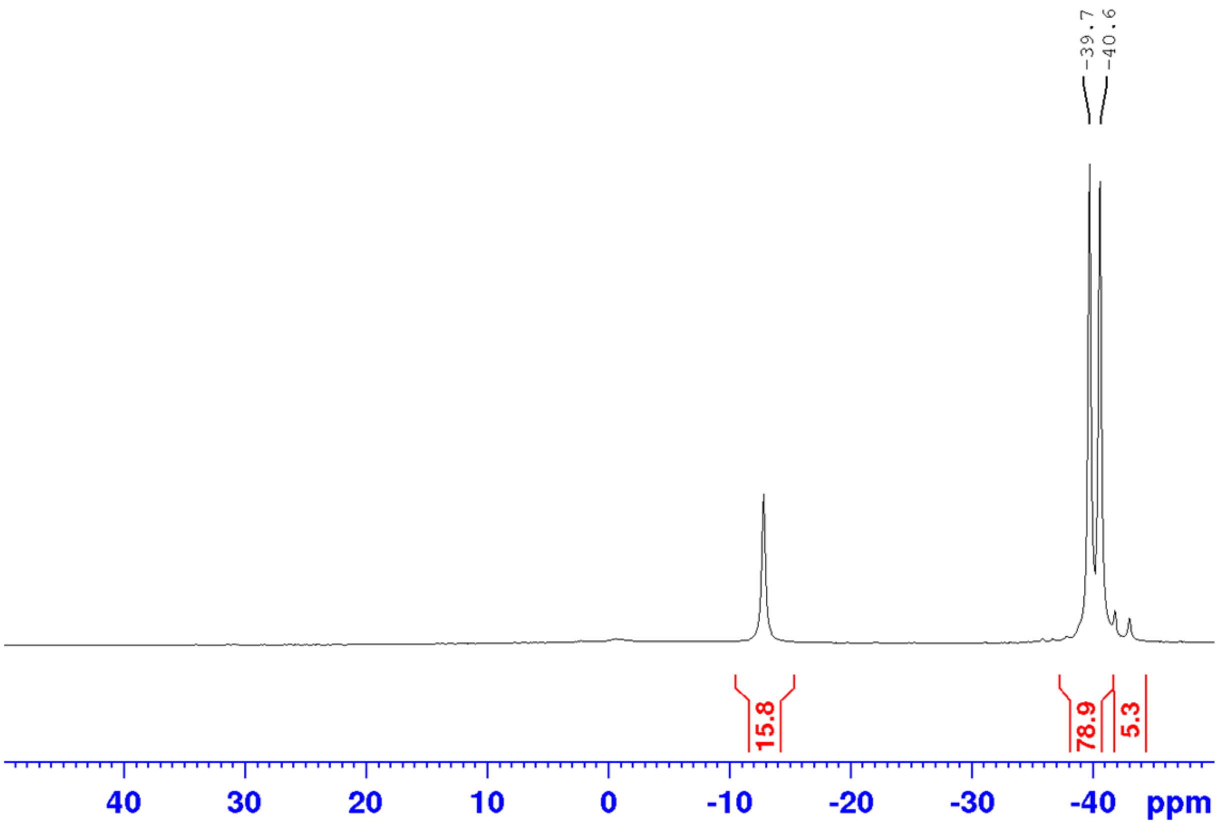

Figure S6: $^{11}B\{^{1}H\}$ NMR spectrum (C_6D_6) of **2b**. The signal at -13 ppm is due to $Me_2NH{\cdot}BH_3$ formed as side product.

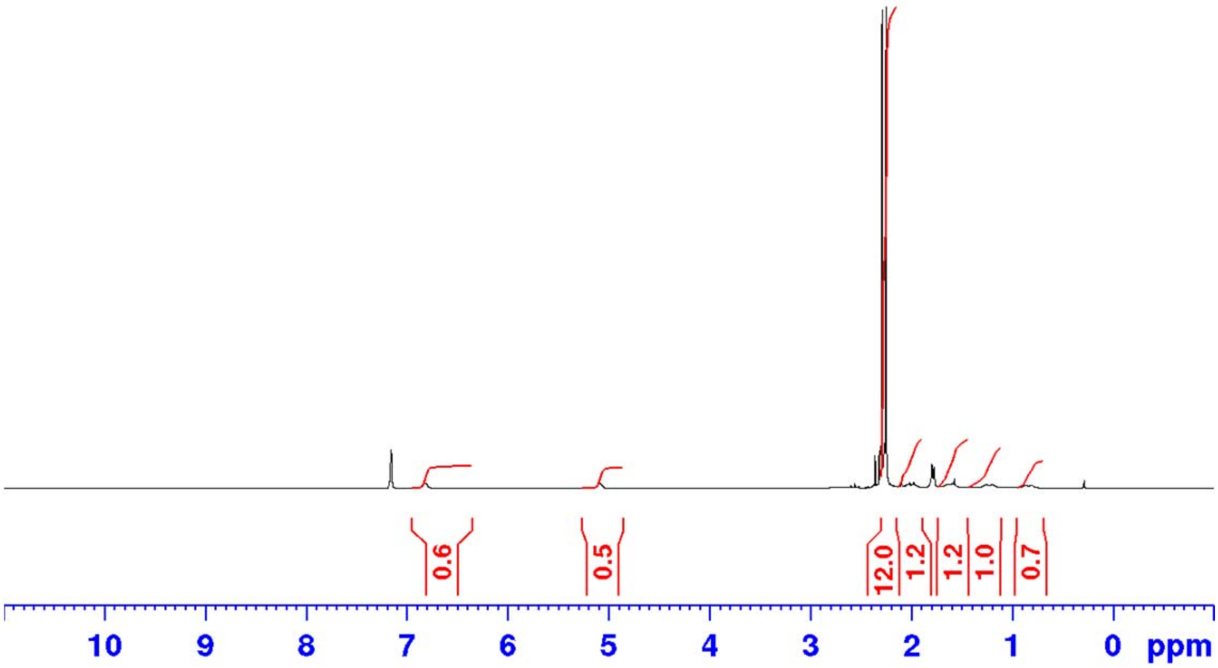

Figure S7: ^{1}H NMR spectrum (C_6D_6) of **2b** (contains 16 mol-% of $Me_2NH{\cdot}BH_3$).

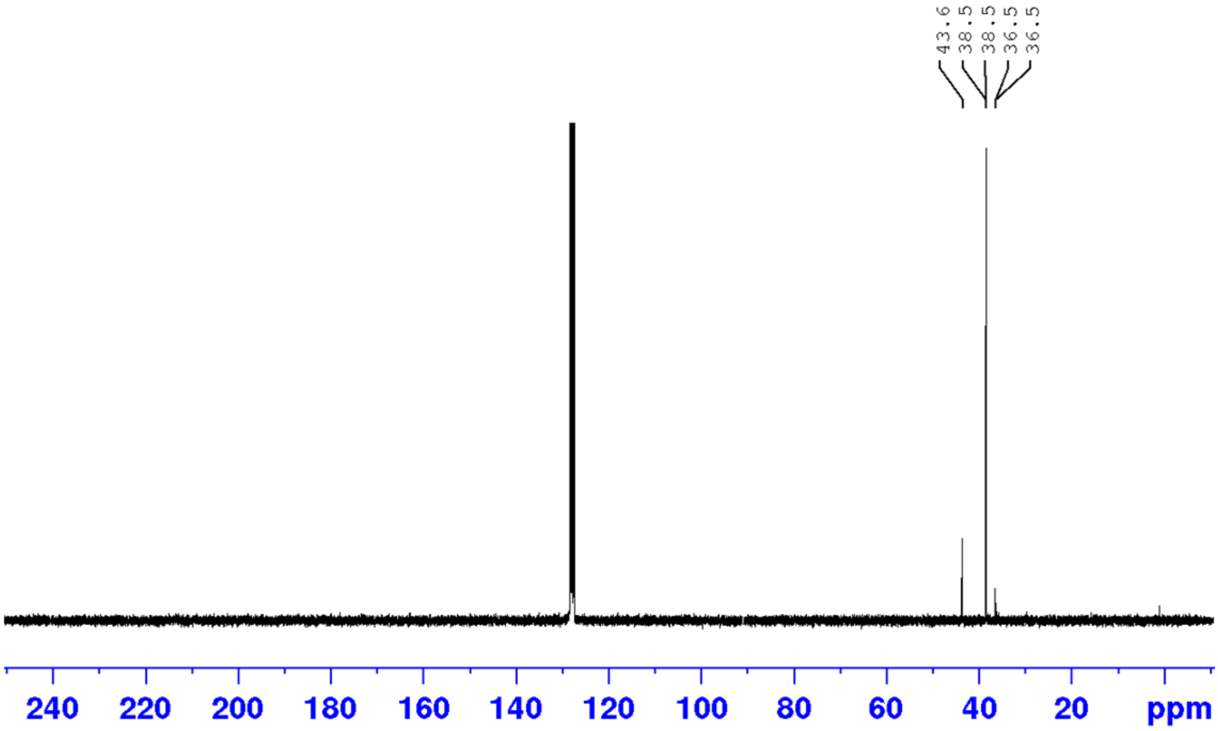

Figure S8: $^{13}C\{^{1}H\}$ NMR spectrum (C_6D_6) of **2b** (contains 16 mol-% of $Me_2NH{\cdot}BH_3$).

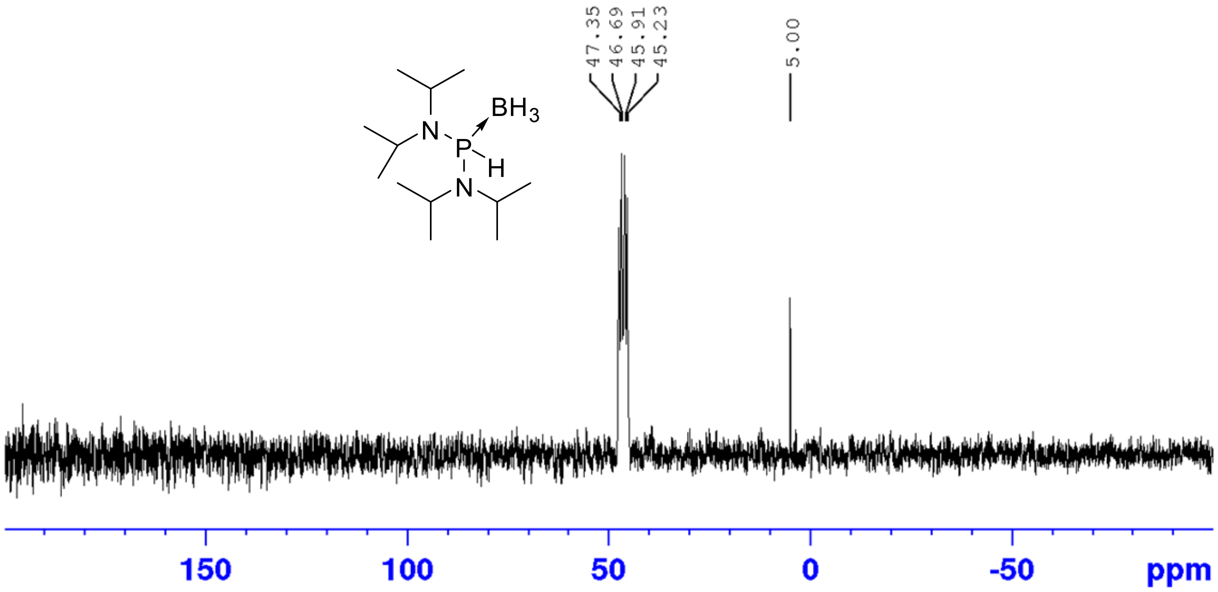

Figure S9: $^{31}P\{^{1}H\}$ NMR spectrum (C_6D_6) of **2c**.

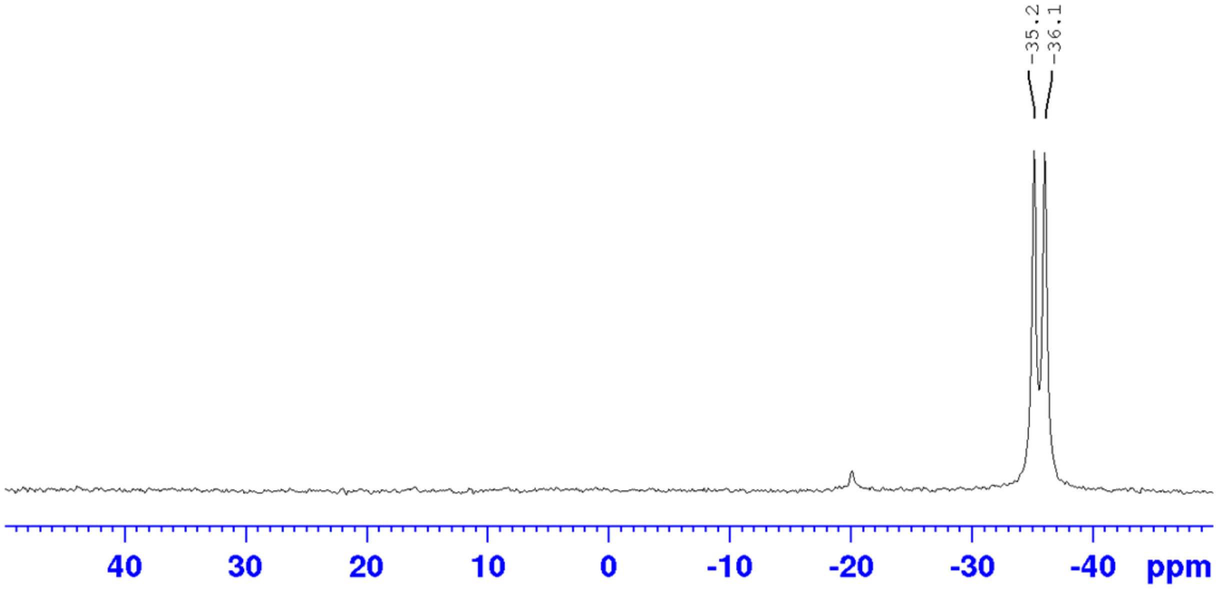

Figure S10: $^{11}B\{^{1}H\}$ NMR spectrum (C_6D_6) of **2c**.

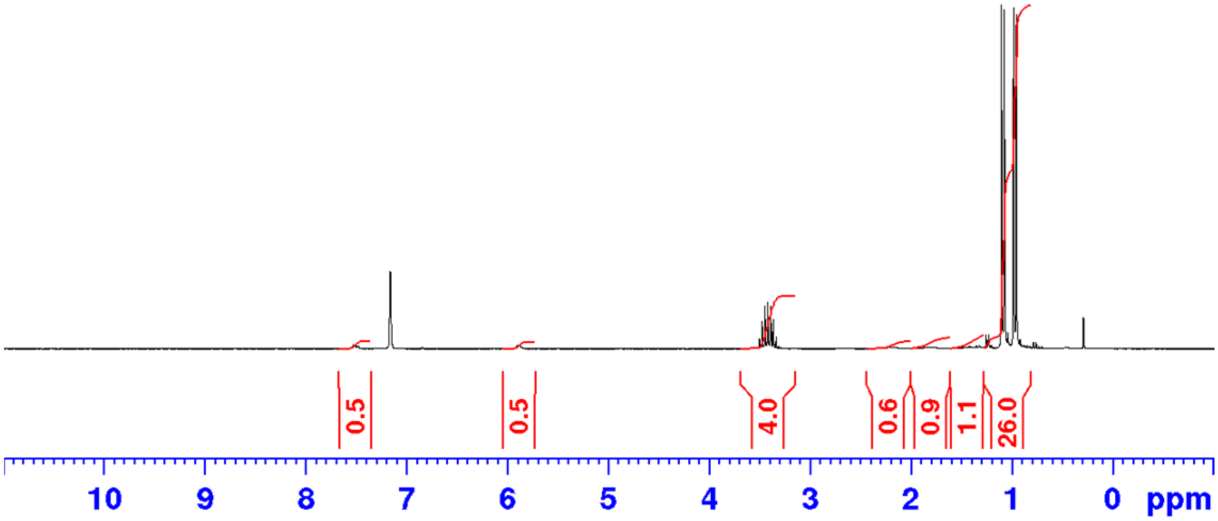

Figure S11: ^{1}H NMR spectrum (C_6D_6) of **2c**.

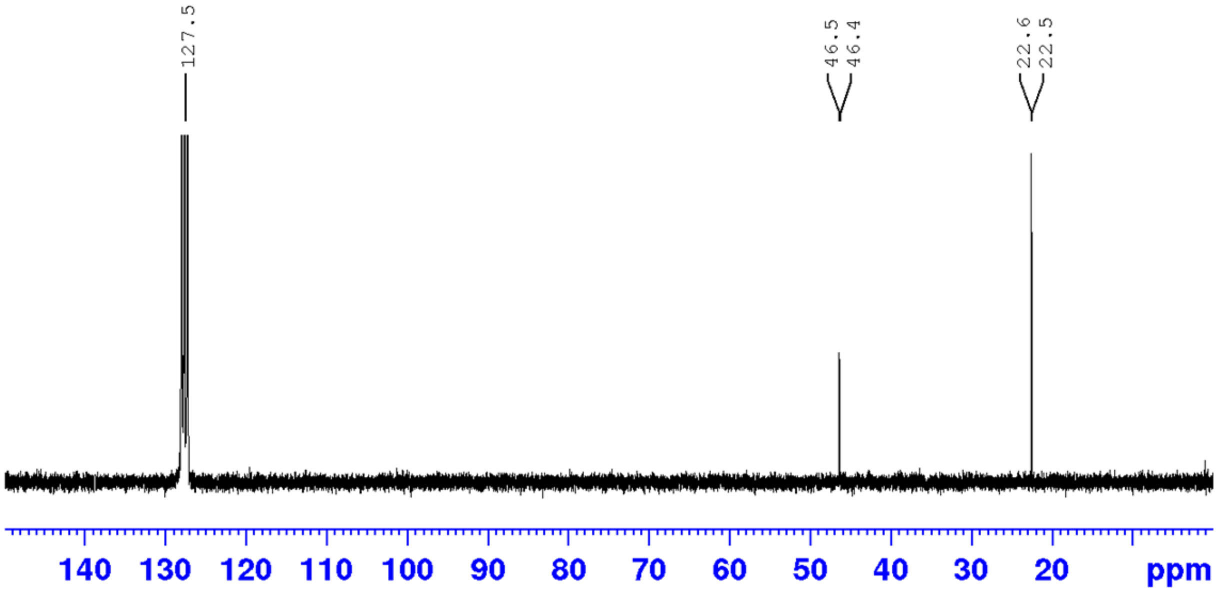

Figure S12: $^{13}C\{^{1}H\}$ NMR spectrum (C_6D_6) of **2c**.

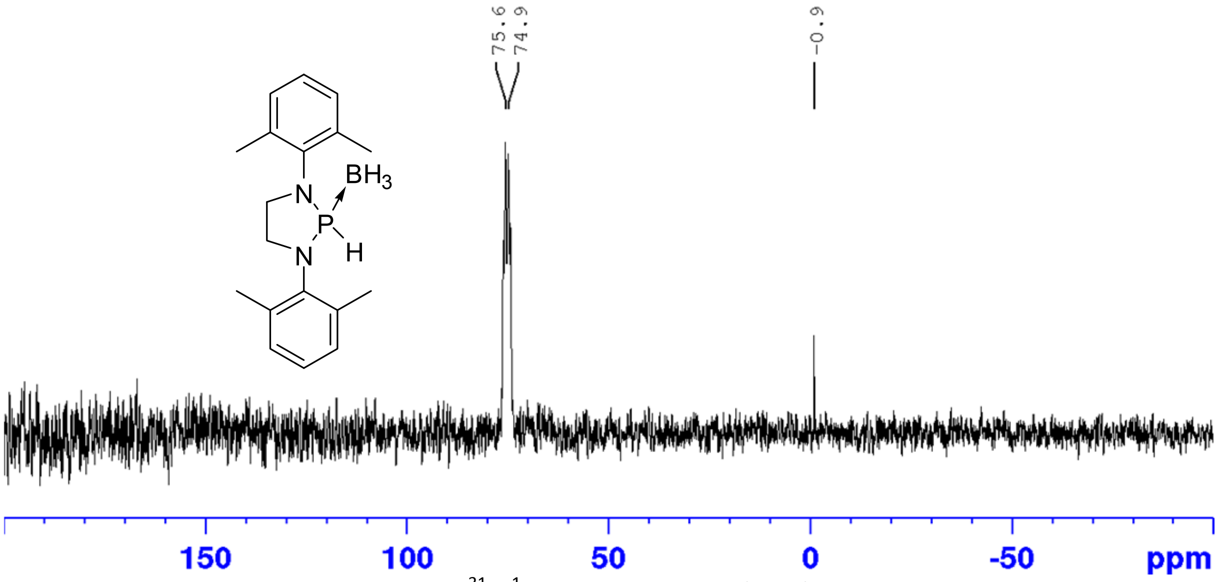

Figure S13: $^{31}P\{^{1}H\}$ NMR spectrum (C_6D_6) of **2d**.

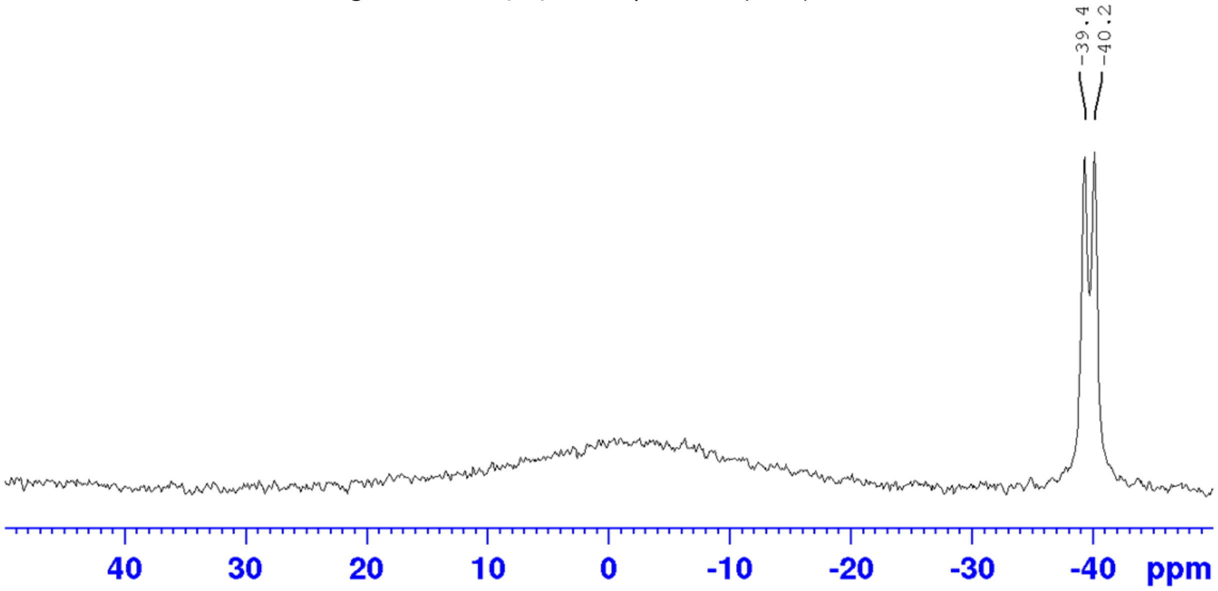

Figure S14: $^{11}B\{^{1}H\}$ NMR spectrum (C_6D_6) of **2d** (the bump around -2 ppm is part of the probe background).

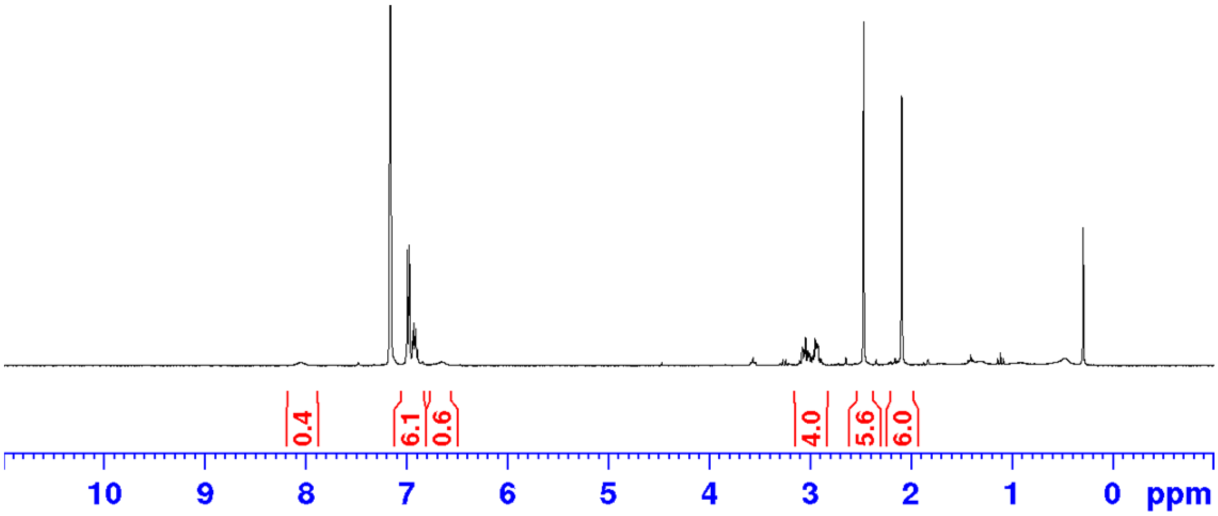

Figure S15: ^{1}H NMR spectrum (C_6D_6) of **2d**.

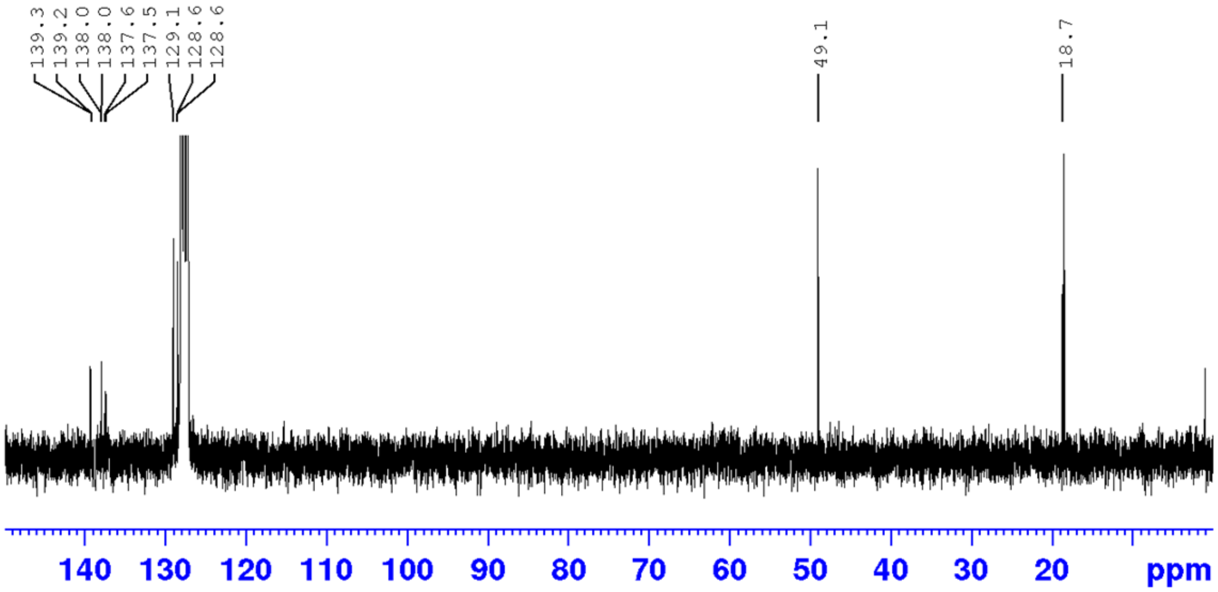

Figure S16: $^{13}C\{^{1}H\}$ NMR spectrum (C_6D_6) of **2d**.

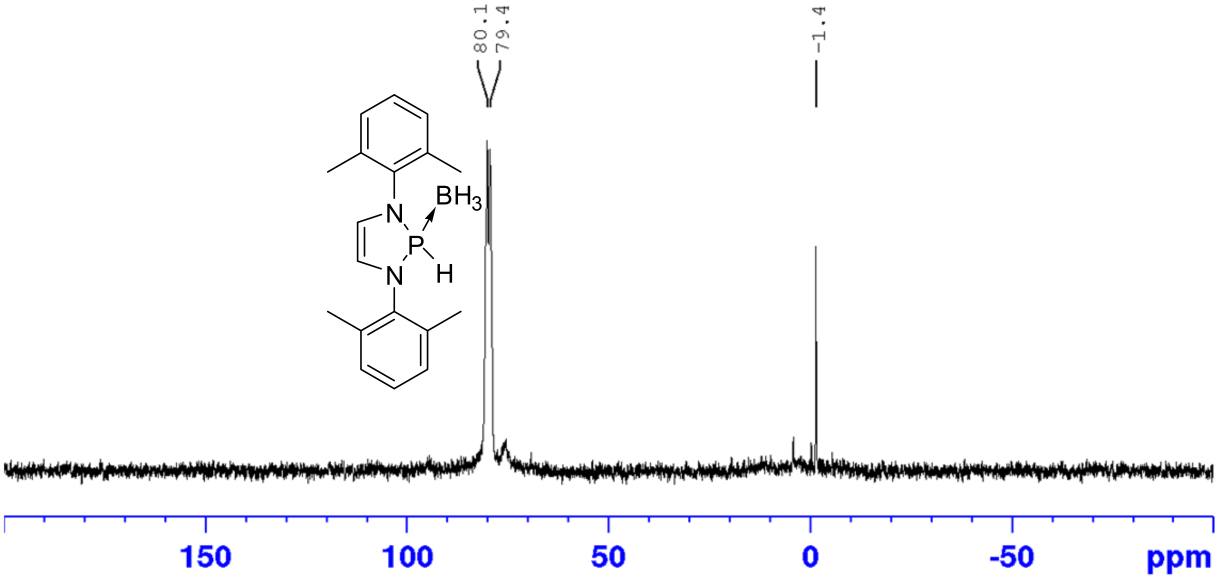

Figure S17: $^{31}P\{^{1}H\}$ NMR spectrum (C_6D_6) of **2f**.

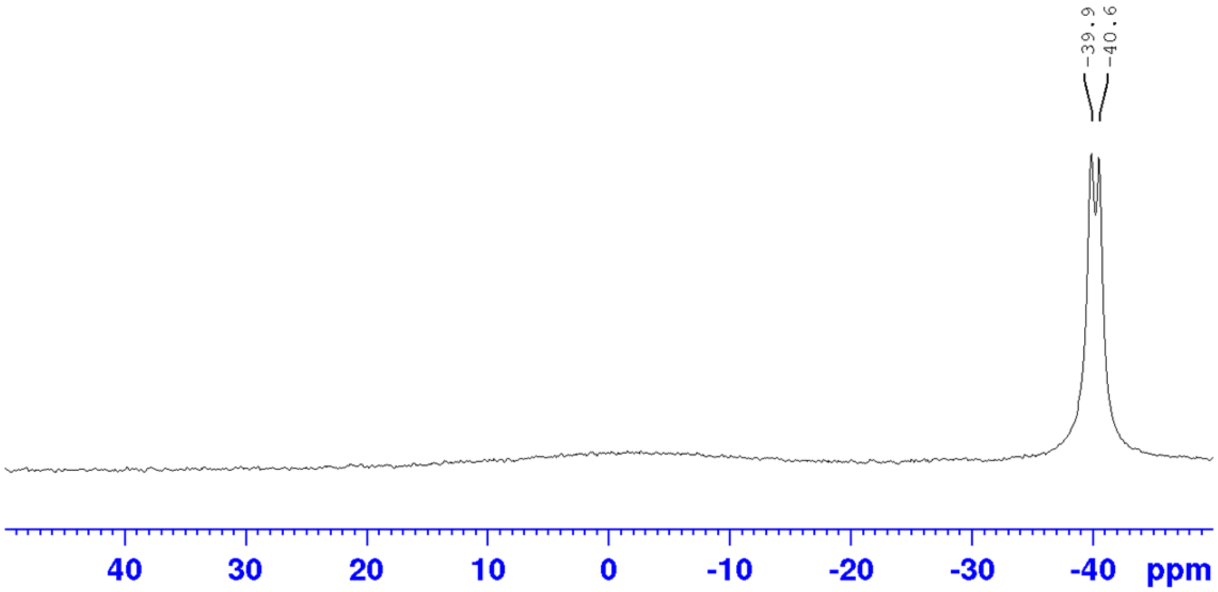

Figure S18: $^{11}B\{^1H\}$ NMR spectrum (C_6D_6) of **2f** (the broad bump is part of the probe background).

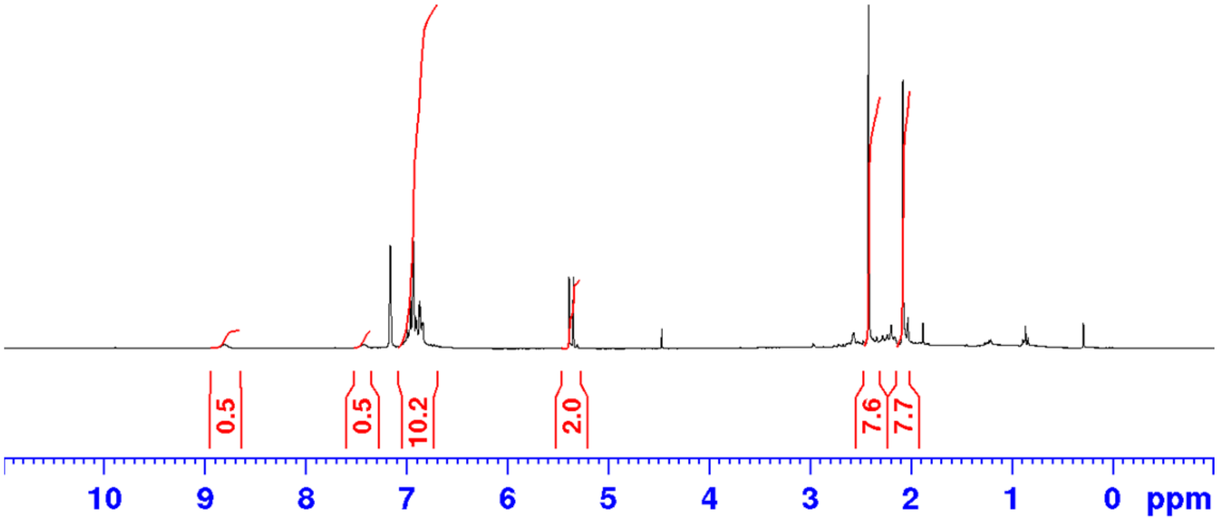

Figure S19: ^1H NMR spectrum (C_6D_6) of **2f**.

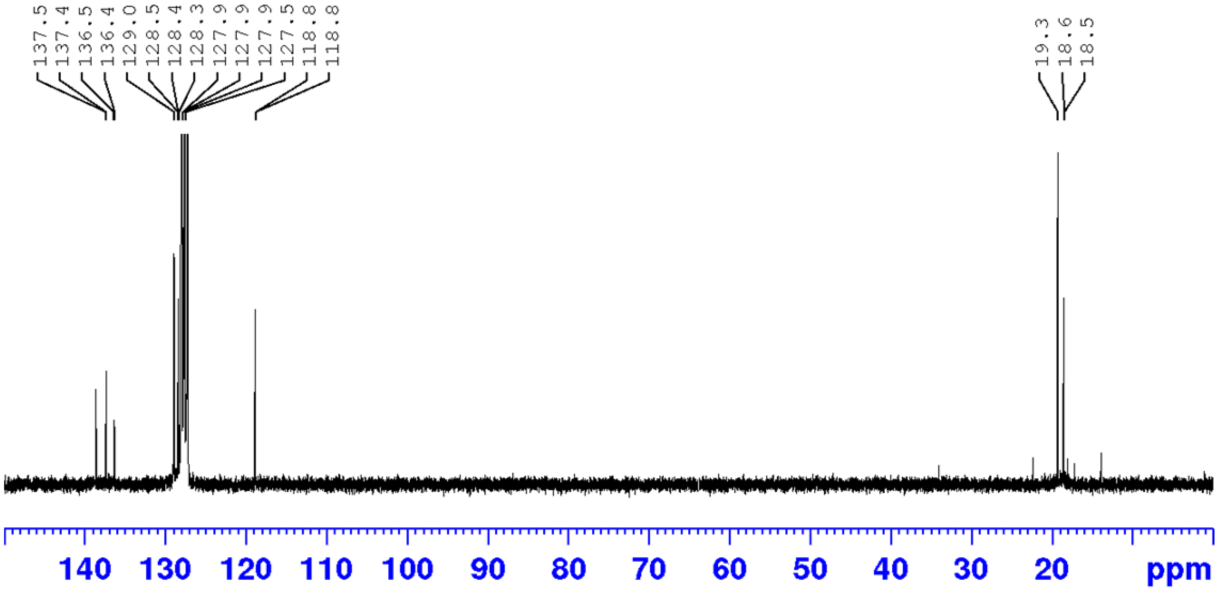

Figure S20: $^{13}C\{^1H\}$ NMR spectrum (C_6D_6) of **2f**.

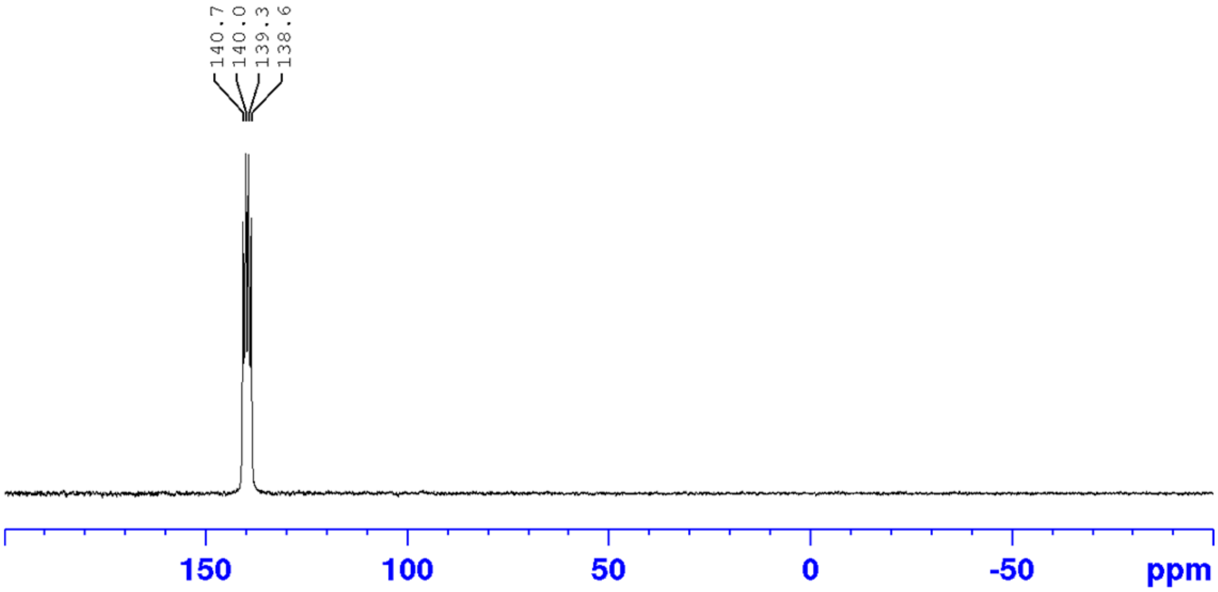

Figure S21: $^{31}P\{^{1}H\}$ NMR spectrum (C_6D_6) of **3b**

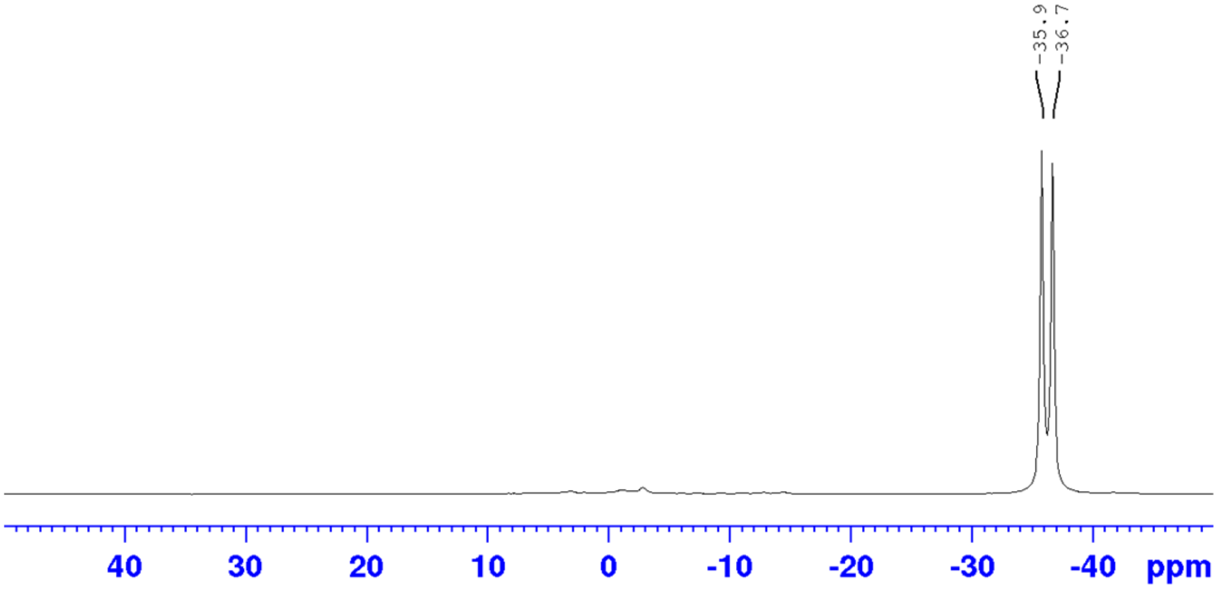

Figure S22: $^{11}B\{^{1}H\}$ NMR spectrum (C_6D_6) of **3b**.

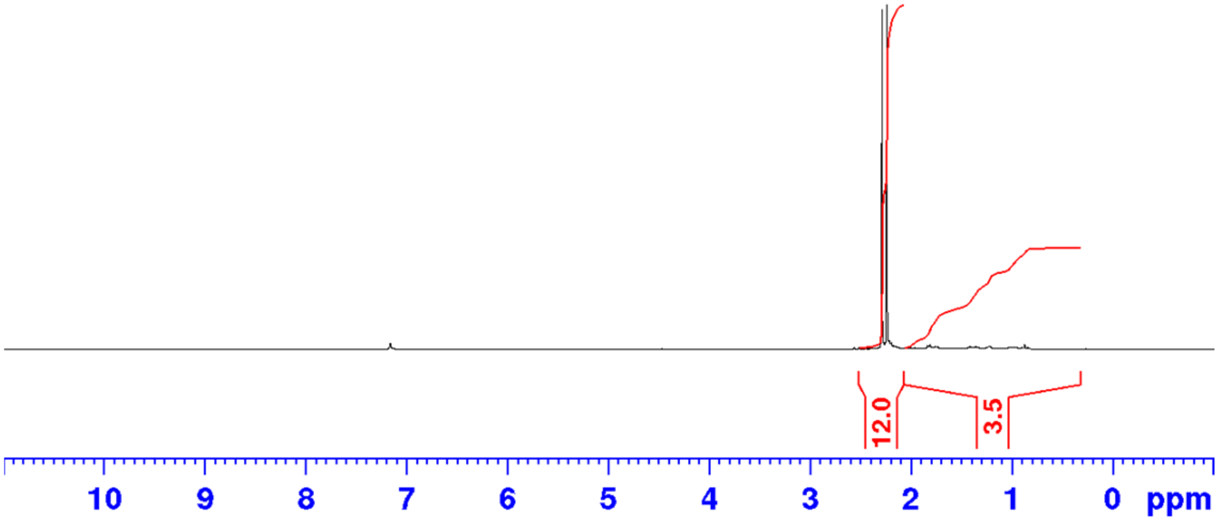

Figure S23: ^{1}H NMR spectrum (C_6D_6) of **3b**.

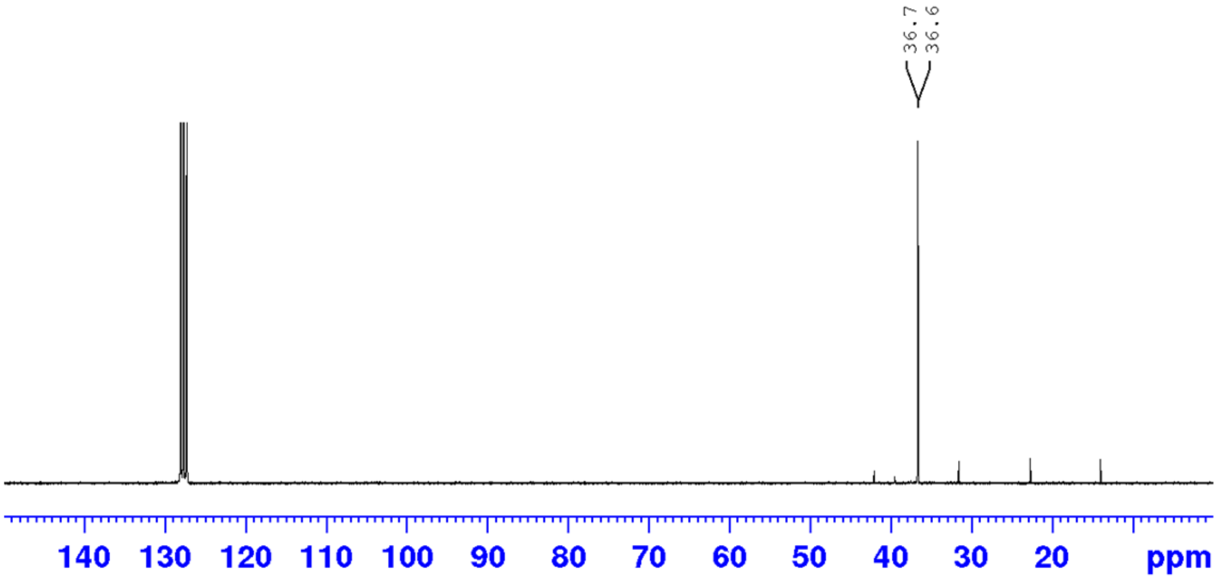

Figure S24: $^{13}C\{^{1}H\}$ NMR spectrum (C_6D_6) of **3b**.

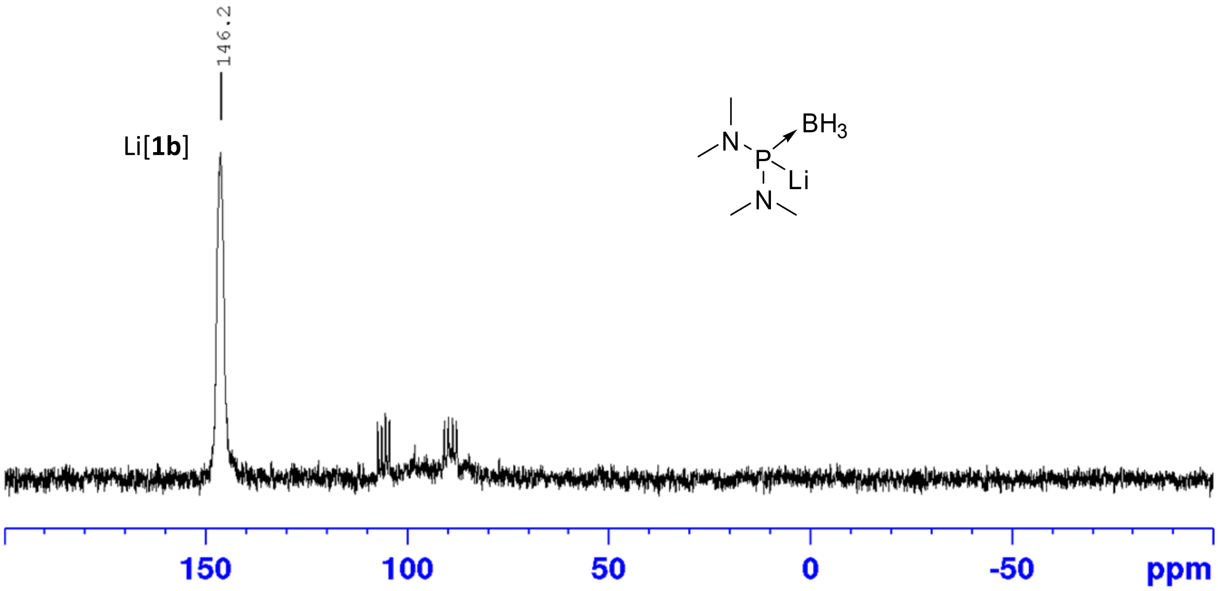

Figure S25: $^{31}P\{^{1}H\}$ NMR spectrum (THF-d_8) of the reaction of **2b** with LiHMDS.

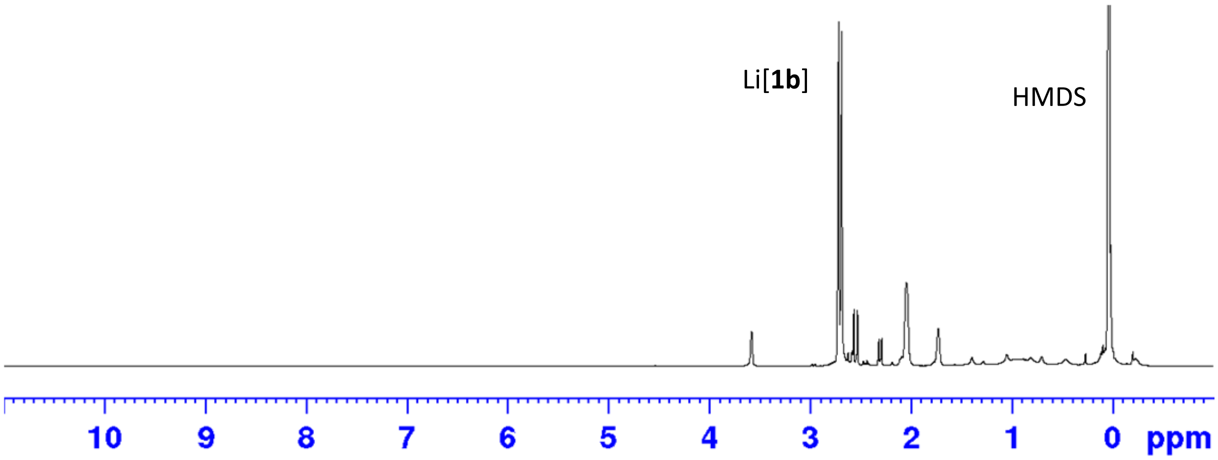

Figure S26: ^{1}H NMR spectrum (THF-d_8) of the reaction of **2b** with LiHMDS.

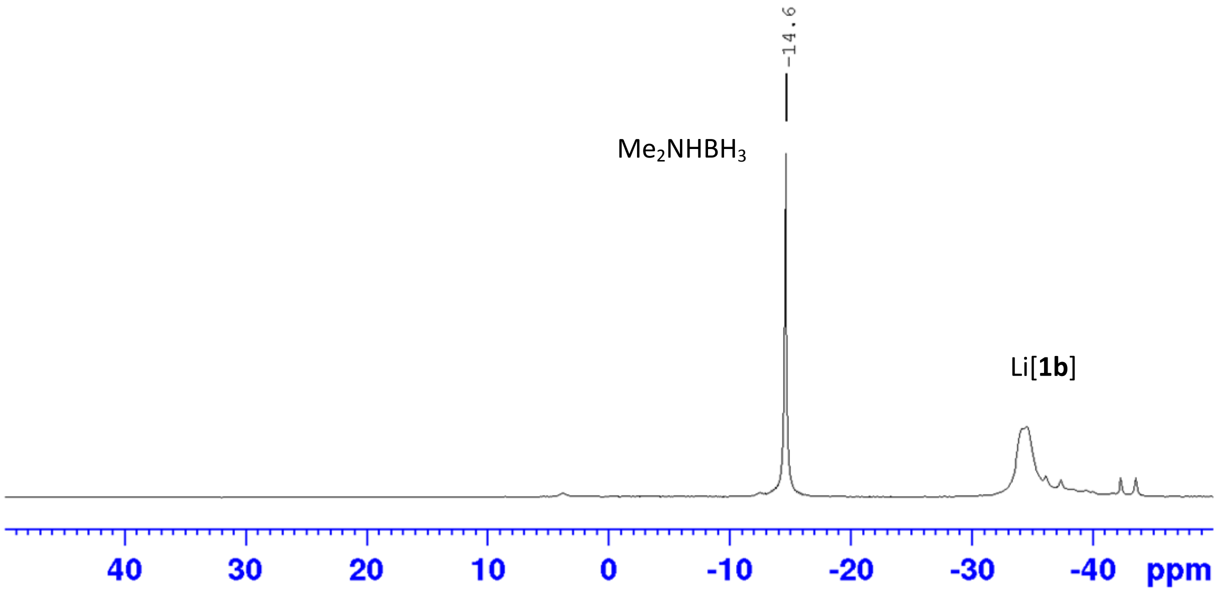

Figure S27: ^{11}B{^{1}H} NMR spectrum (THF-d_8) of the reaction of **2b** with LiHMDS.

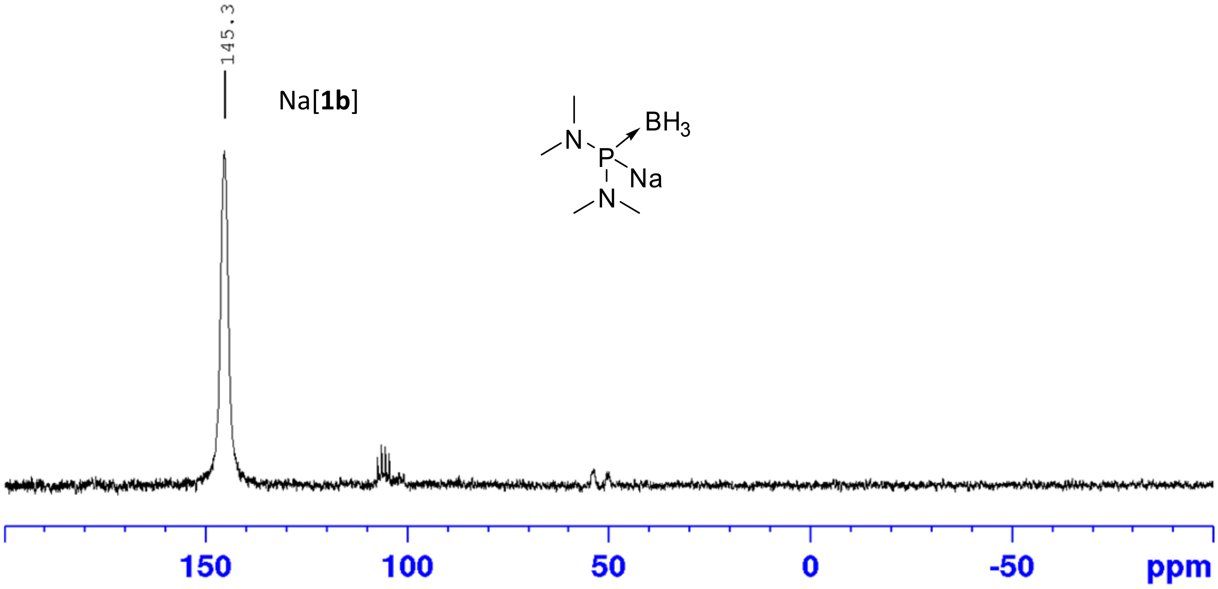

Figure S28: ^{31}P{^{1}H} NMR spectrum (THF-d_8) of the reaction of **2b** with NaHMDS.

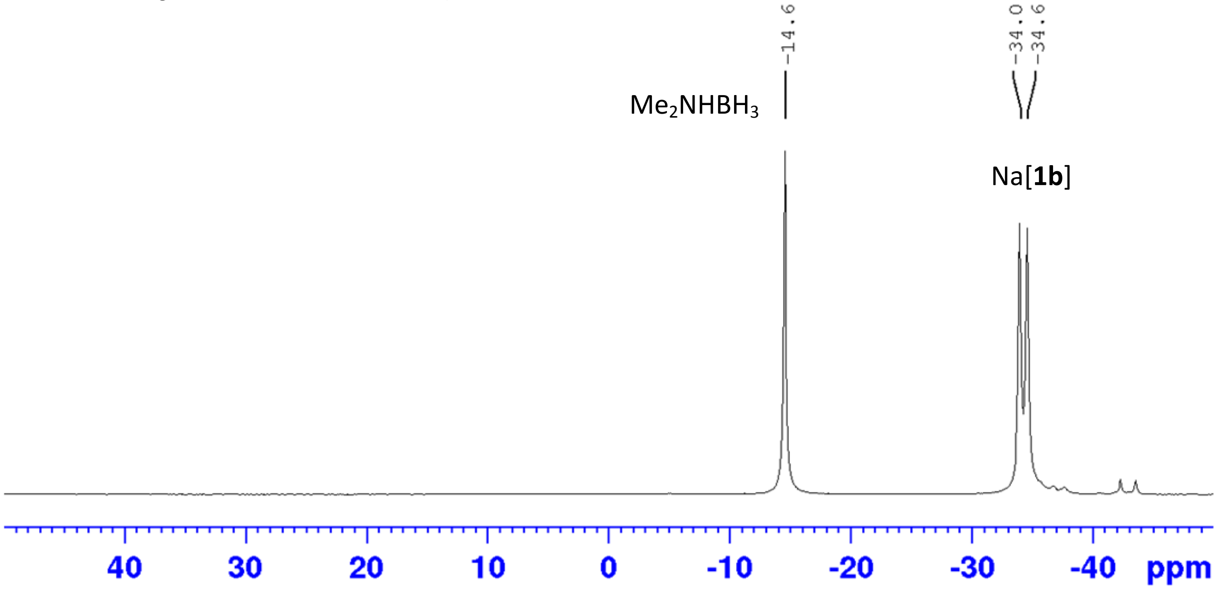

Figure S29: ^{11}B{^{1}H} NMR spectrum (THF-d_8) of the reaction of **2b** with NaHMDS.

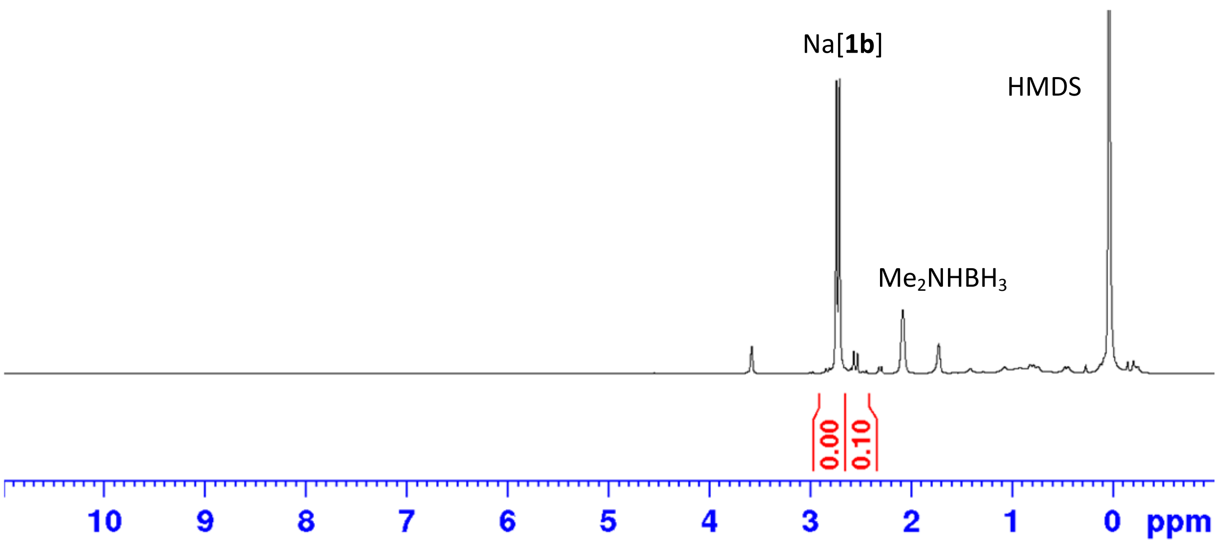

Figure S30: ^{1}H NMR spectrum (THF-d$_8$) of the reaction of **2b** with NaHMDS.

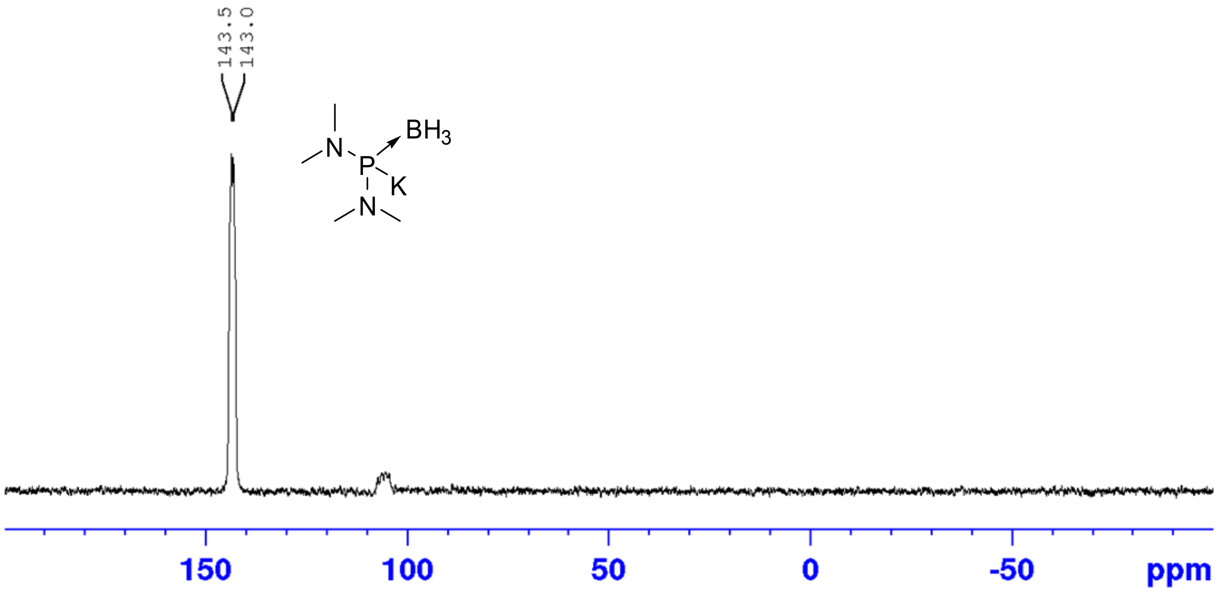

Figure S31: ^{31}P{^{1}H} NMR spectrum (THF-d$_8$) of the reaction of **2b** with KHMDS.

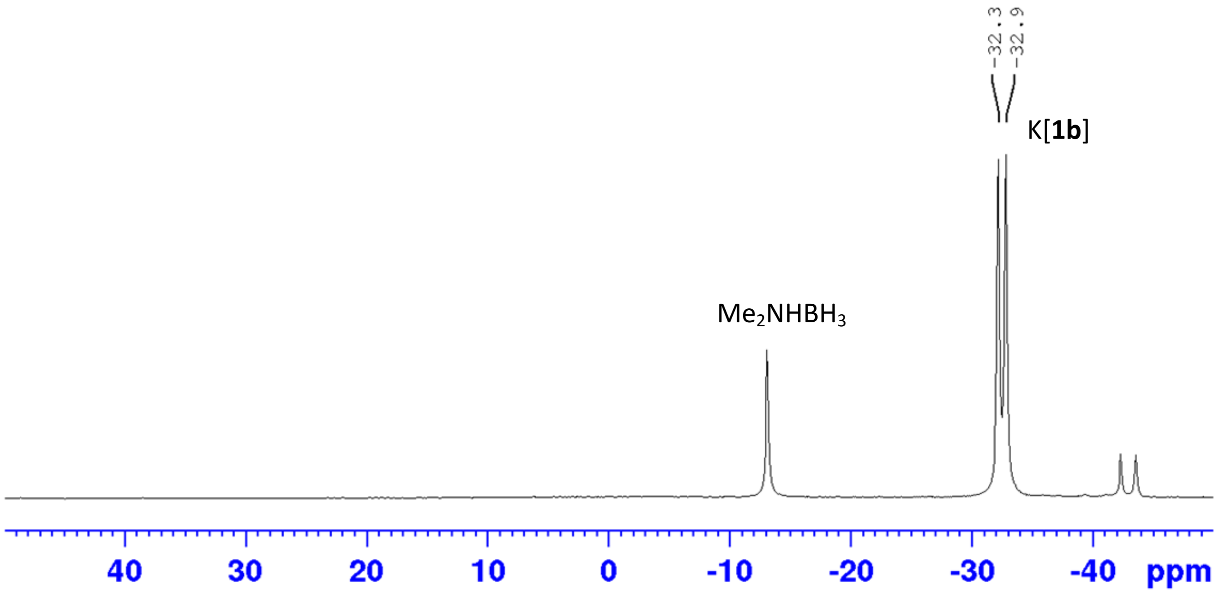

Figure S31: ^{11}B{^{1}H} NMR spectrum (THF-d$_8$) of the reaction of **2b** with KHMDS.

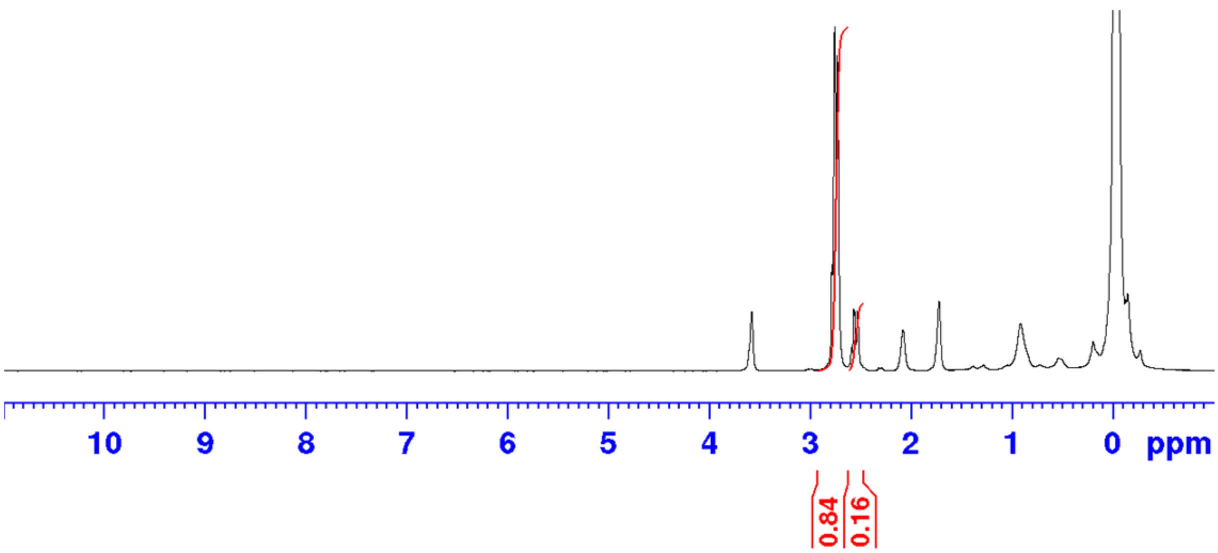

Figure S32: ^{1}H NMR spectrum (THF-d_8) of the reaction of **2b** with KHMDS.

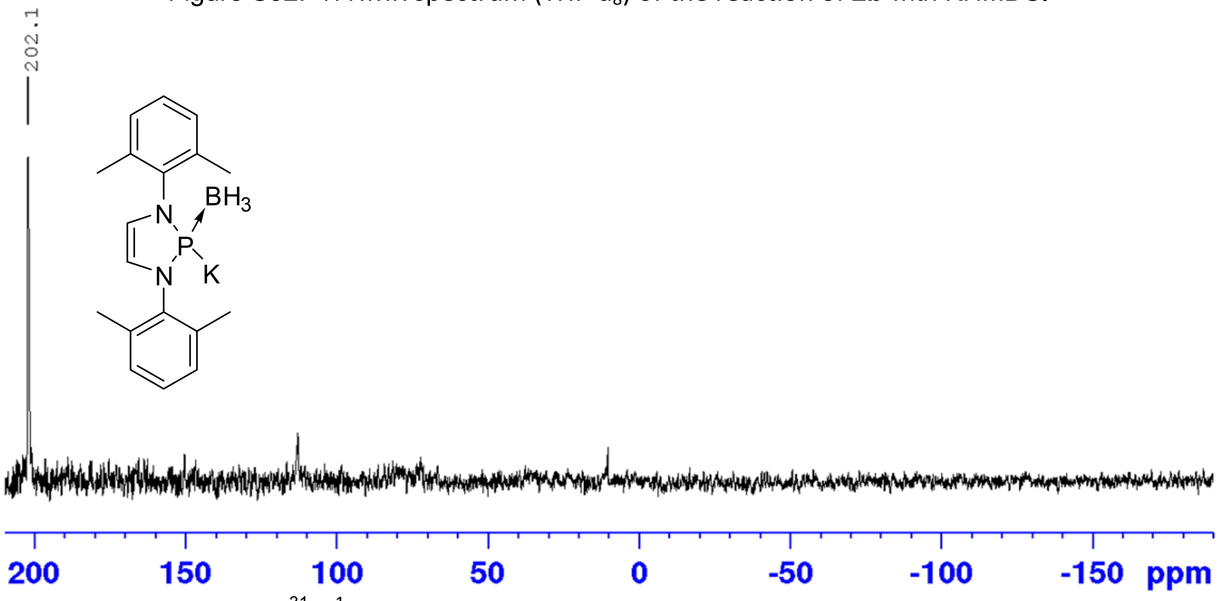

Figure S33: $^{31}P\{^{1}H\}$ NMR spectrum (THF-d_8) of the reaction of **2f** with KHMDS.

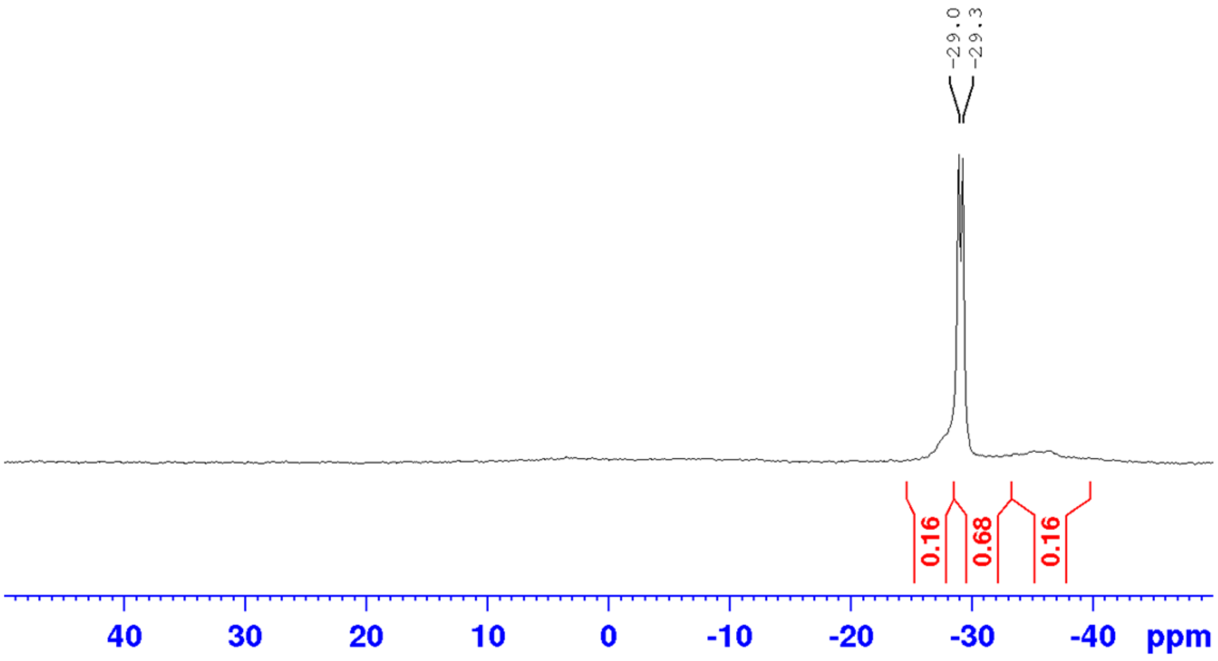

Figure S33: $^{31}P\{^{1}H\}$ NMR spectrum (THF-d_8) of the reaction of **2f** with KHMDS.

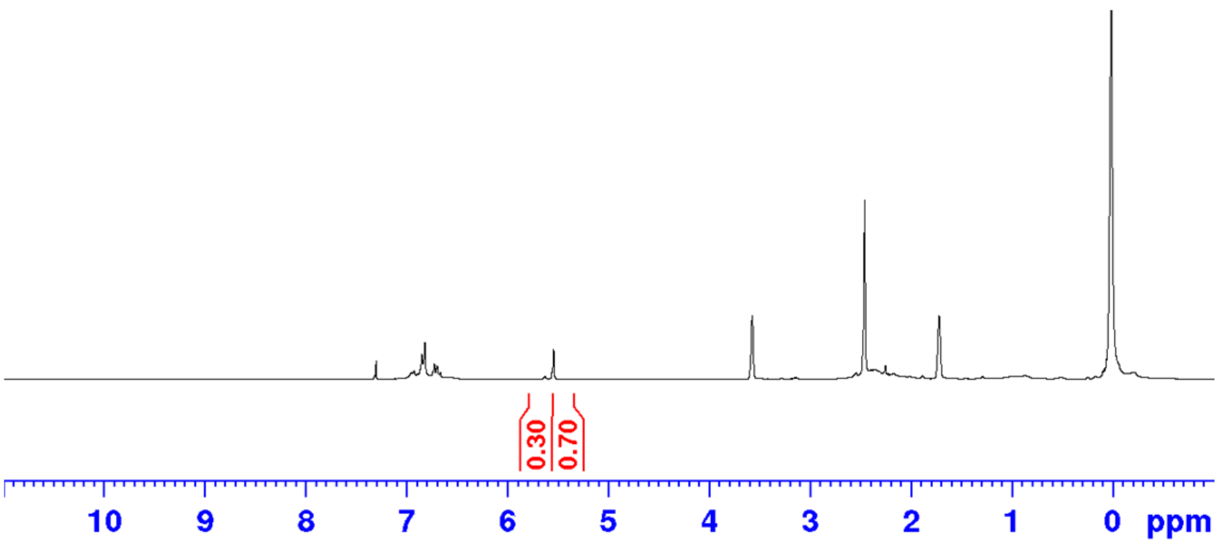

Figure S34: ^{1}H NMR spectrum (THF-d_8) of the reaction of **2f** with KHMDS.

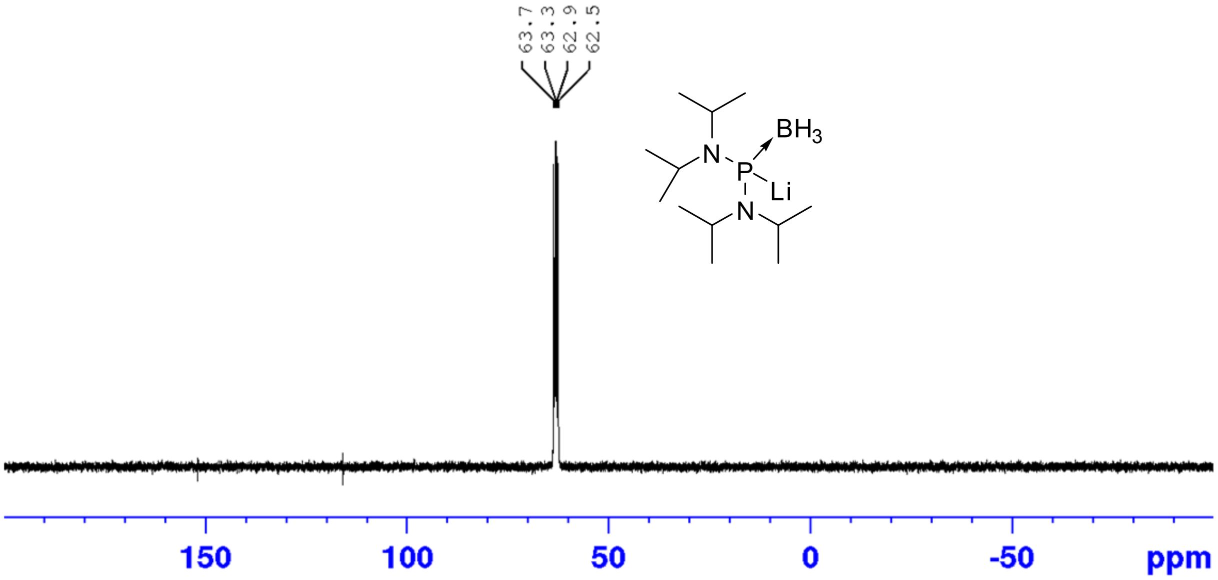

Figure S35: $^{31}P\{^{1}H\}$ NMR spectrum (THF-d_8) of the reaction of **2c** with n-BuLi.

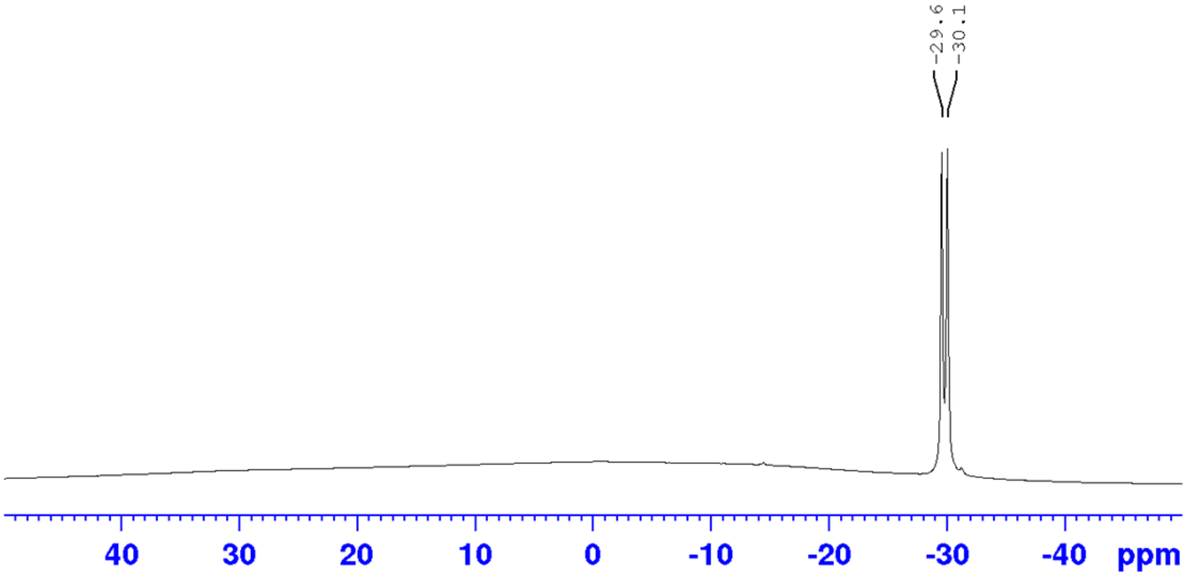

Figure S36: $^{11}B\{^{1}H\}$ NMR spectrum (THF-d_8) of the reaction of **2c** with n-BuLi in THF-d_8. The broad bump is part of the background signal from the probe.

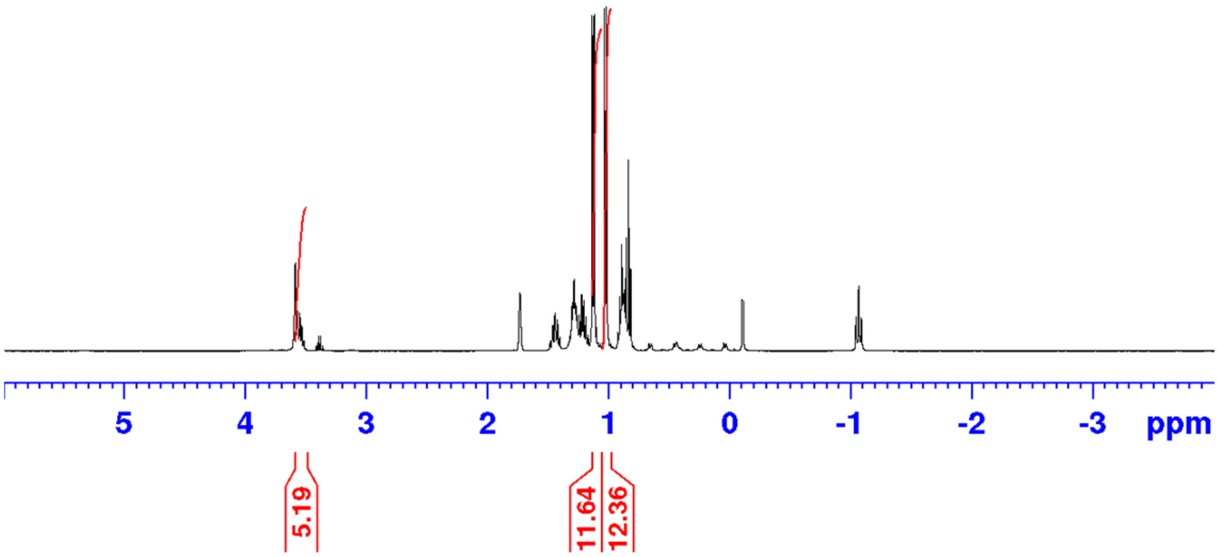

Figure S37: ^{1}H NMR spectrum (THF-d_8) of the reaction of **2c** with n-BuLi.

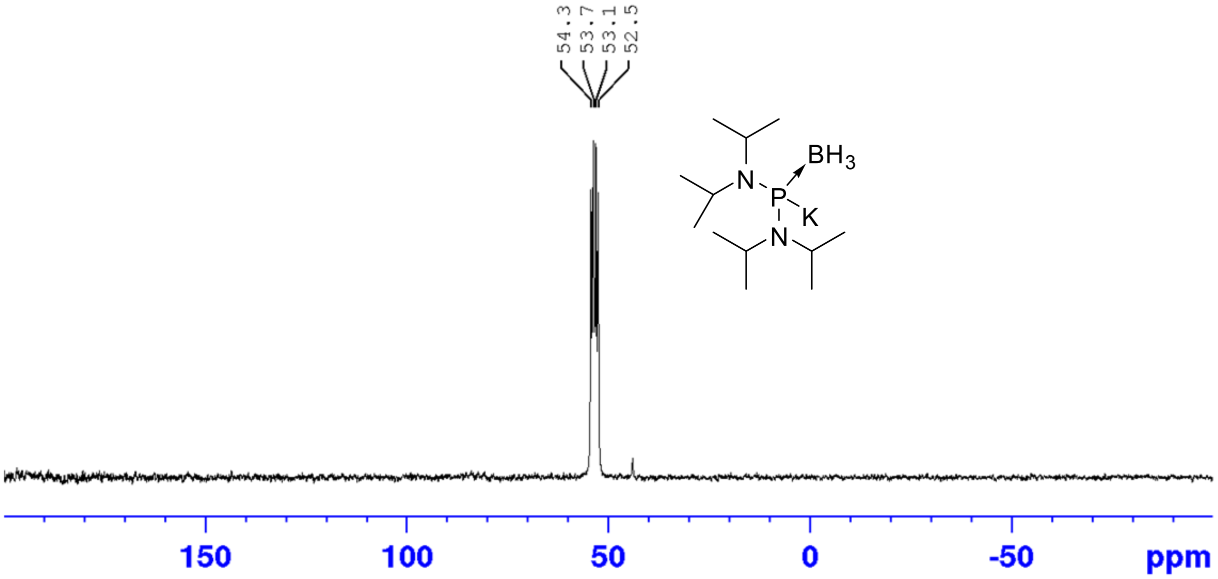

Figure S38: ^{31}P{^{1}H} NMR spectrum (C_6D_6) of the reaction of **2c** with KHMDS.

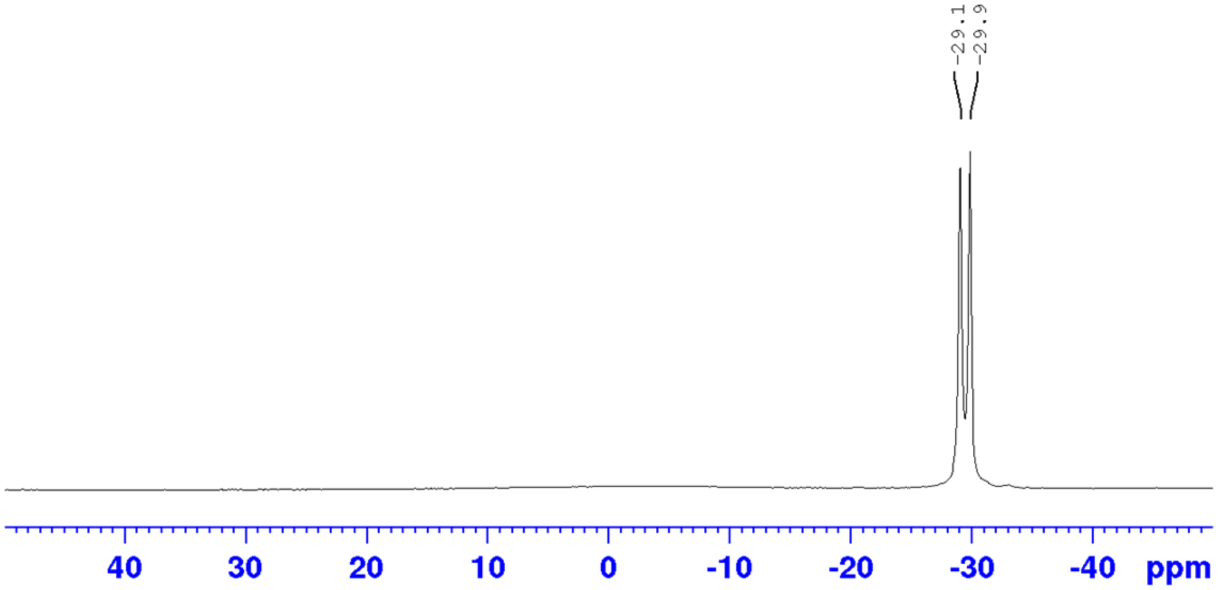

Figure S39: ^{11}B{^{1}H} NMR spectrum (C_6D_6) of the reaction of **2c** with KHMDS.

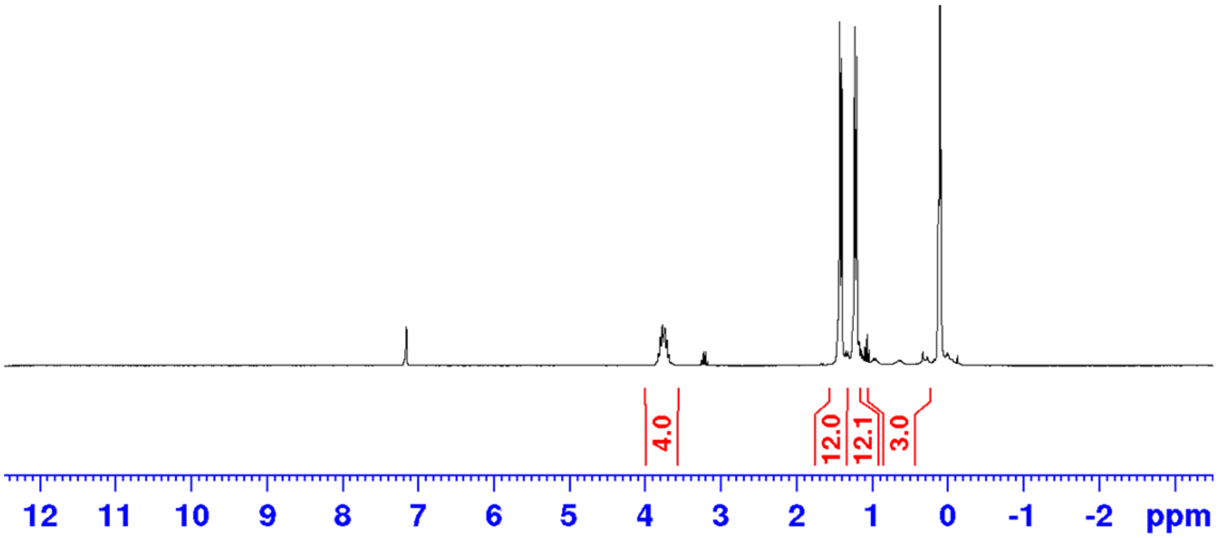

Figure S40: ^{1}H NMR spectrum (C_6D_6) of the reaction of **2c** with KHMDS.

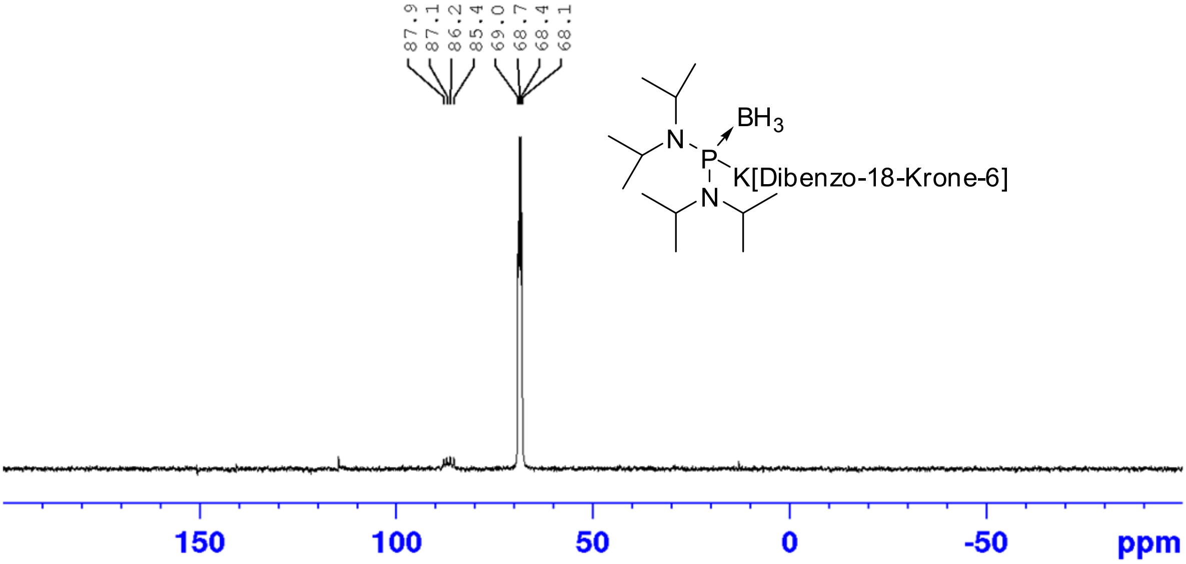

Figure S41: $^{31}P\{^{1}H\}$ NMR spectrum (THF-d_8) of the reaction of **2c** with KHMDS/dibenzo-18-cr-6.

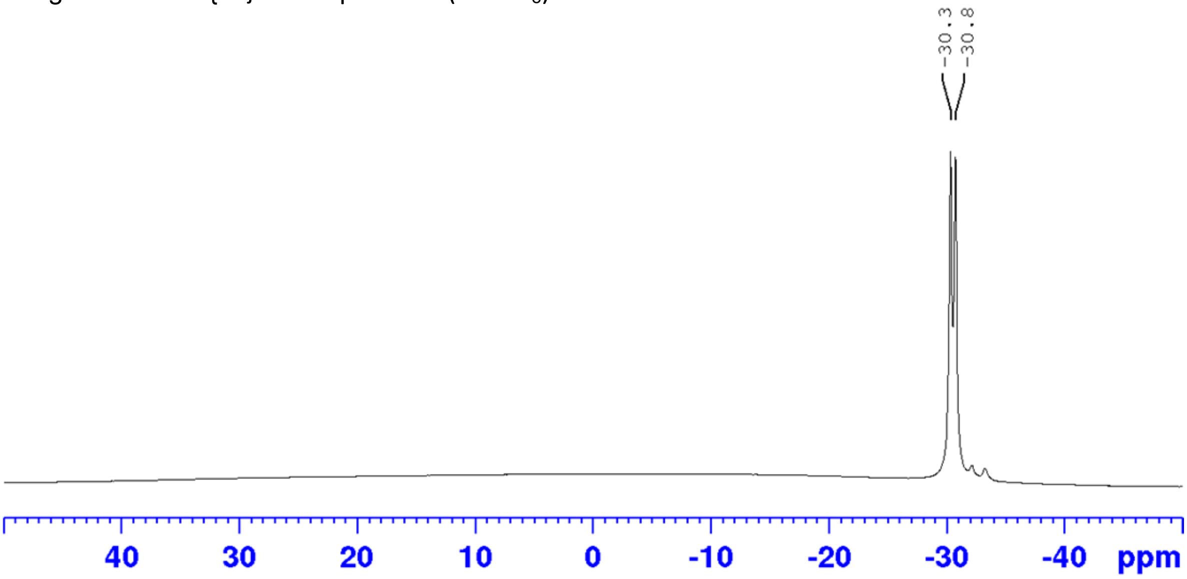

Figure S42: $^{11}B\{^{1}H\}$ NMR spectrum (THF-d_8) of the reaction of **2c** with KHMDS/dibenzo-18-cr-6.

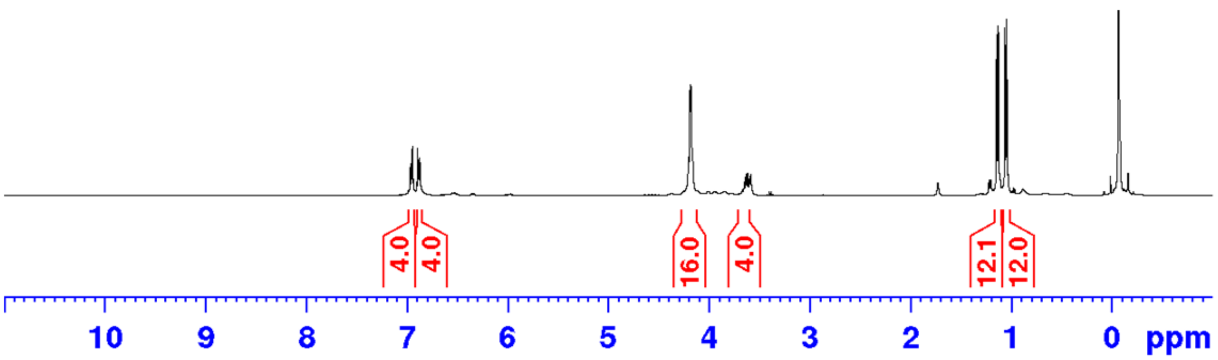

Figure S43: ^{1}H NMR spectrum (THF-d_8) of the reaction of **2c** with KHMDS/dibenzo-18-cr-6.

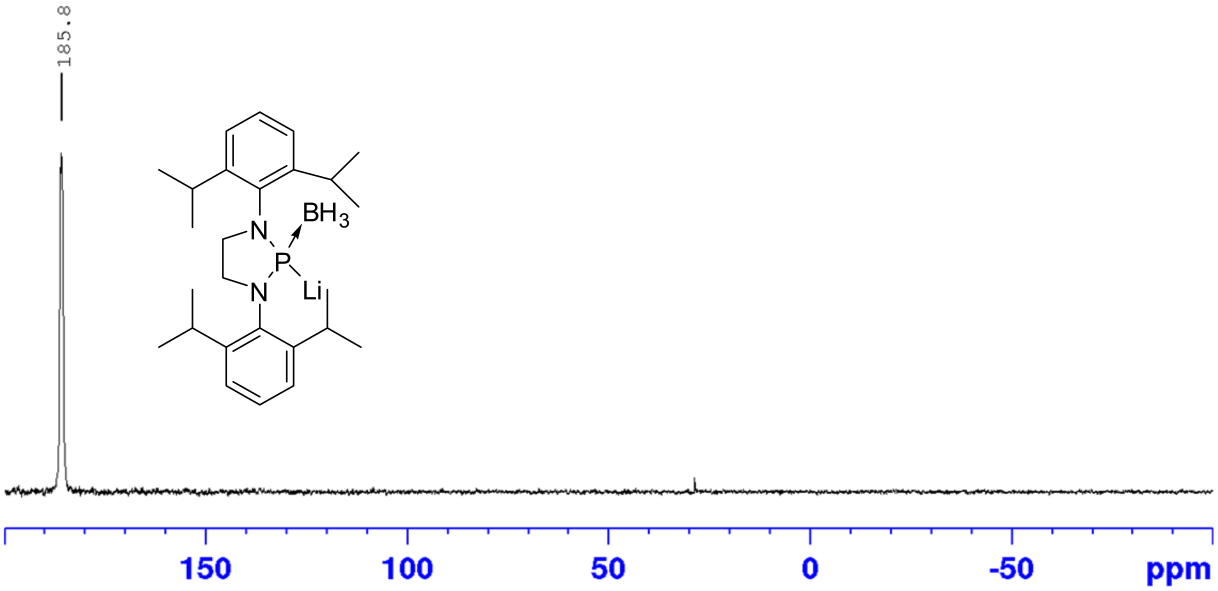

Figure S44: ^{31}P{^{1}H} NMR spectrum (THF-d_8) of the reaction of **2e** with LDA.

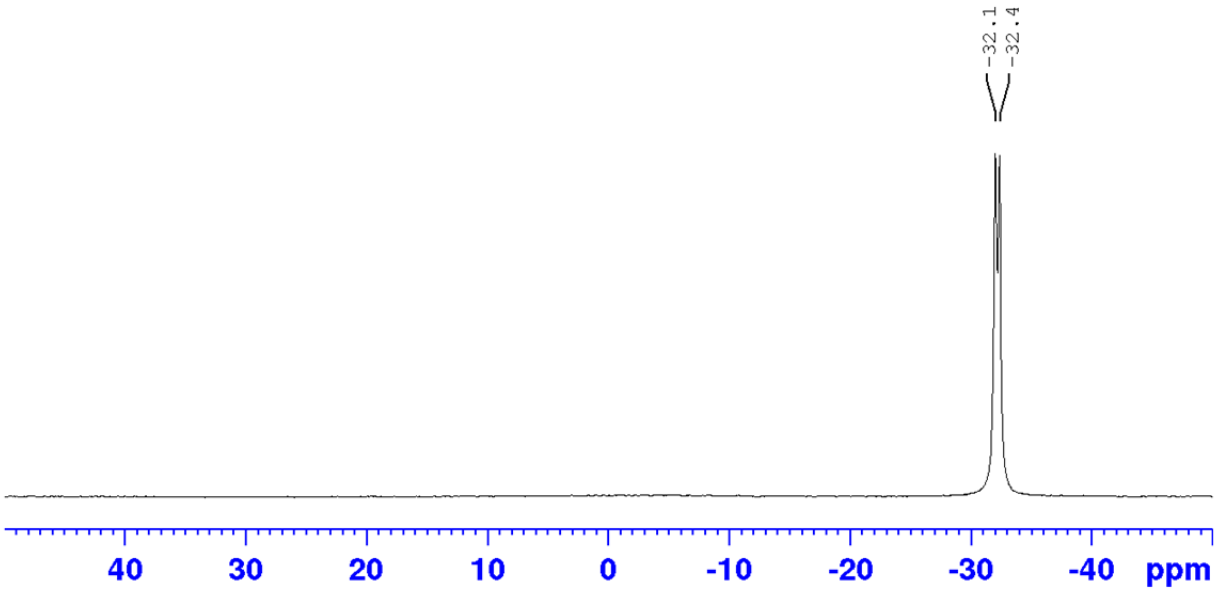

Figure S45: ^{11}B{^{1}H} NMR spectrum (THF-d_8) of the reaction of **2e** with LDA.

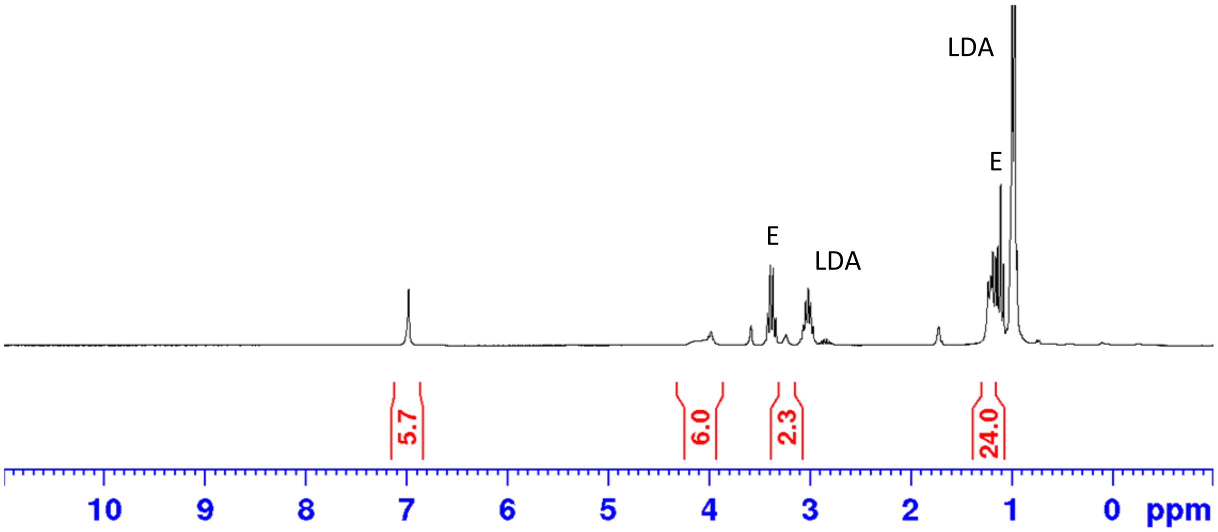

Figure S46: ^{1}H NMR spectrum (THF-d_8) of the reaction of **2e** with LDA (signals of residual Et_2O and LDA labelled).

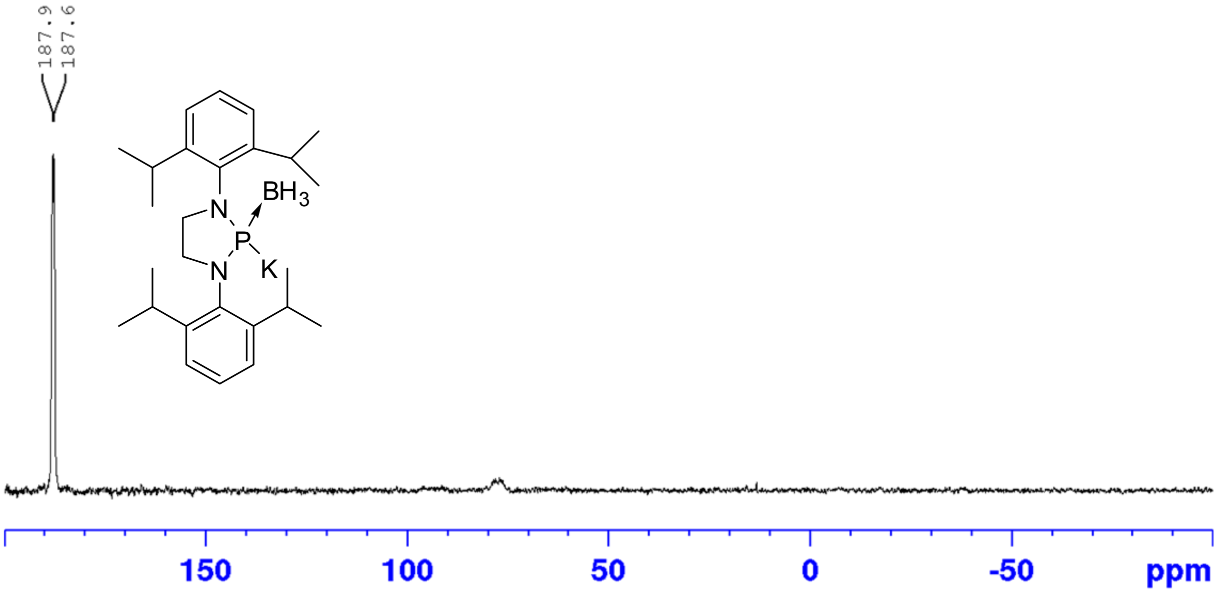

Figure S47: $^{31}P\{^{1}H\}$ NMR spectrum (THF-d_8) of the reaction of **2e** with KHMDS.

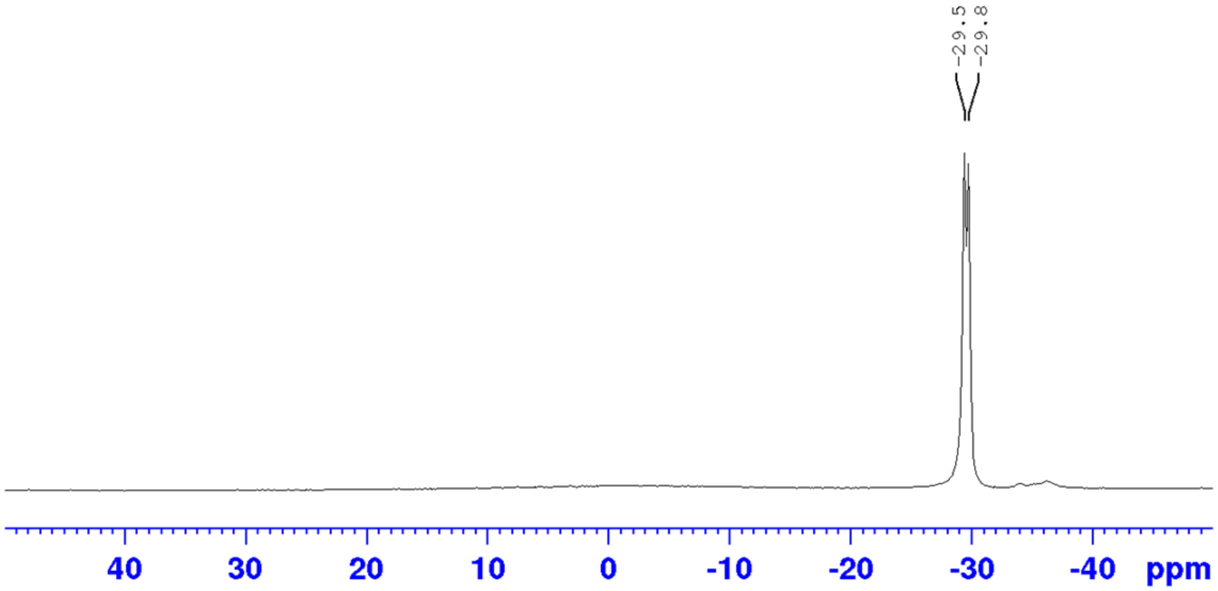

Figure S48: $^{11}B\{^{1}H\}$ NMR spectrum (THF-d_8) of the reaction of **2e** with KHMDS.

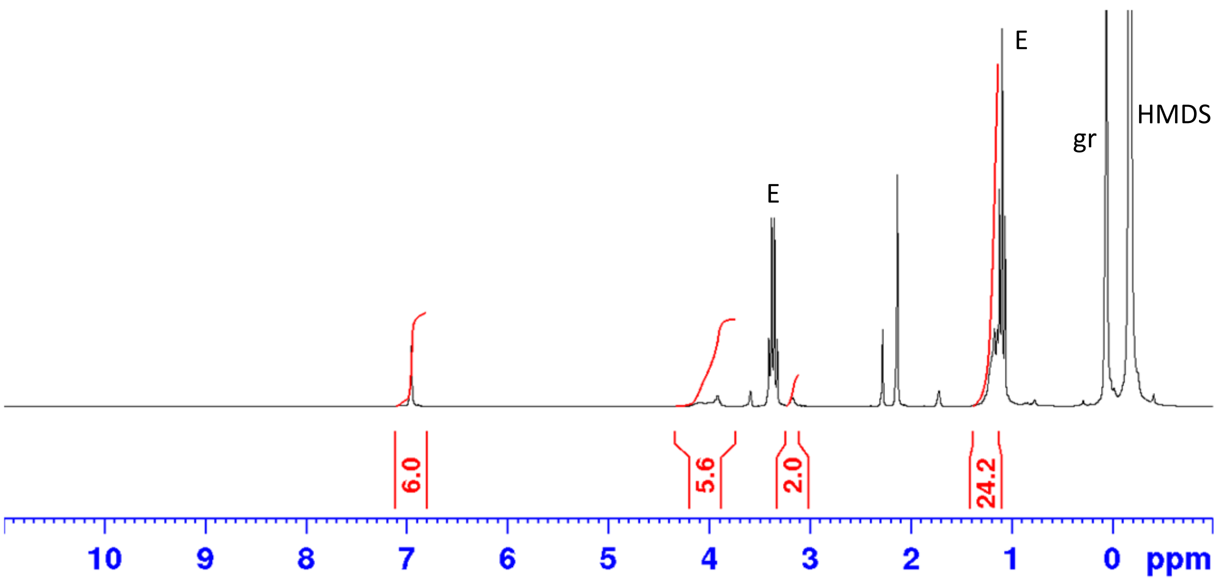

Figure S49: ^{1}H NMR spectrum (THF-d_8) of the reaction of **2e** with KHMDS (signals of residual Et_2O, silicon grease and HMDS labelled).

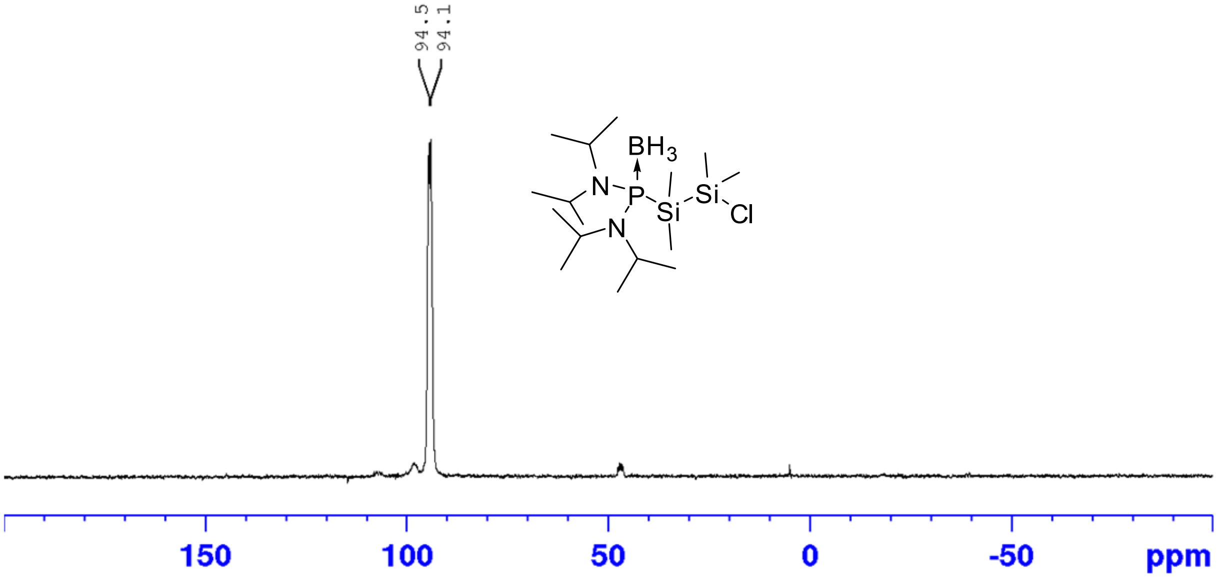

Figure S50: $^{31}P\{^{1}H\}$ NMR spectrum (C_6D_6) of **6c**.

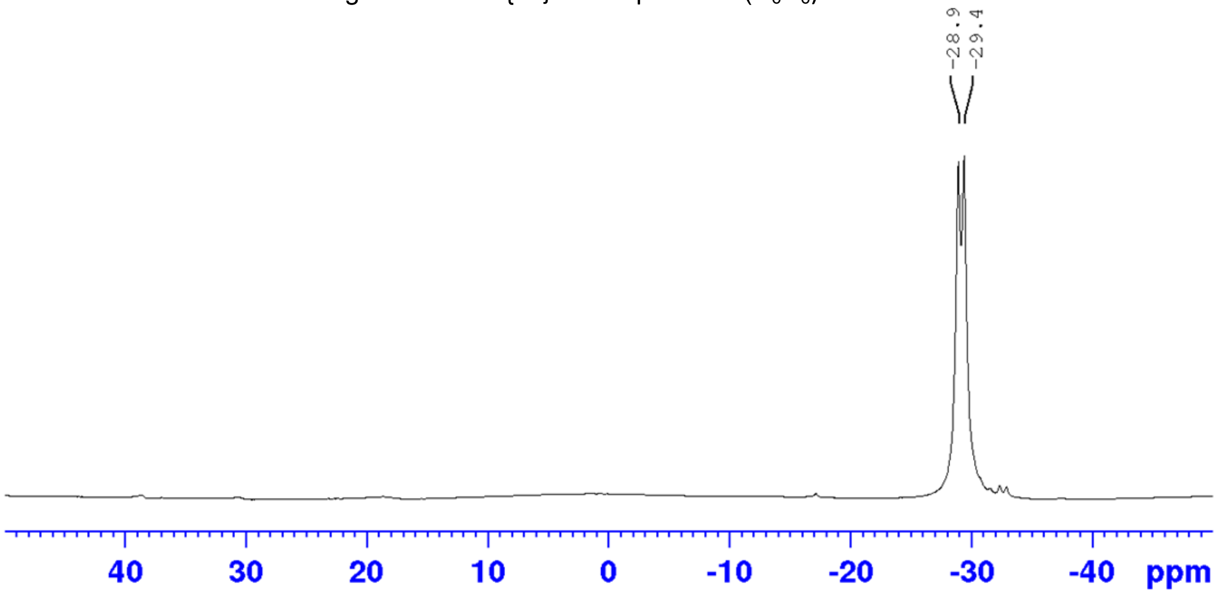

Figure S51: $^{11}B\{^{1}H\}$ NMR spectrum (C_6D_6) of **6c**.

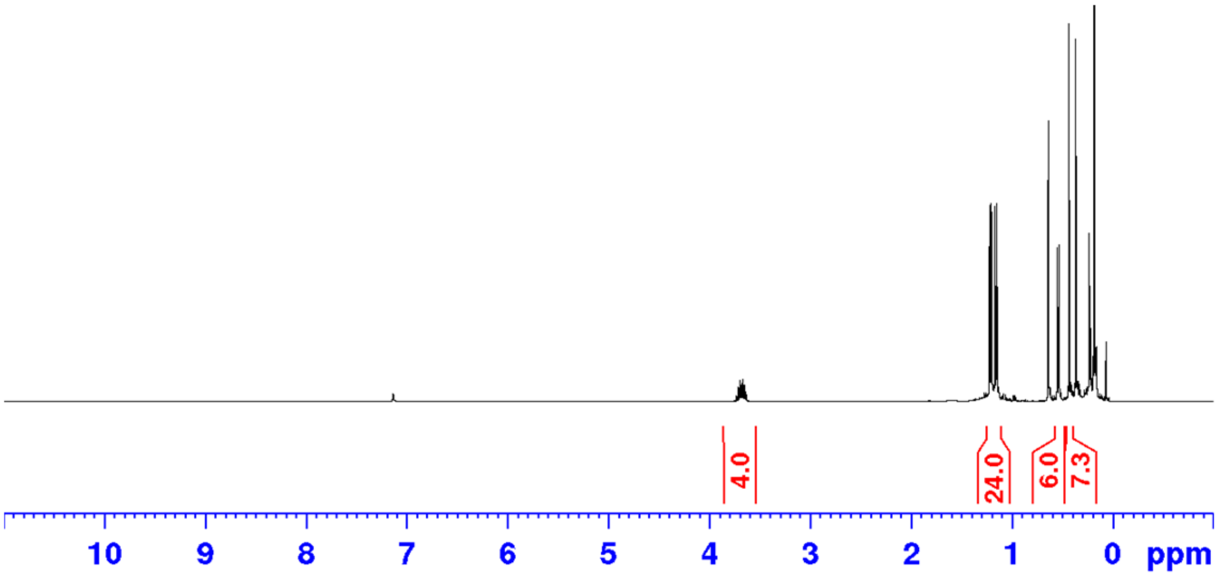

Figure S52: ^{1}H NMR spectrum (C_6D_6) of **6c**.

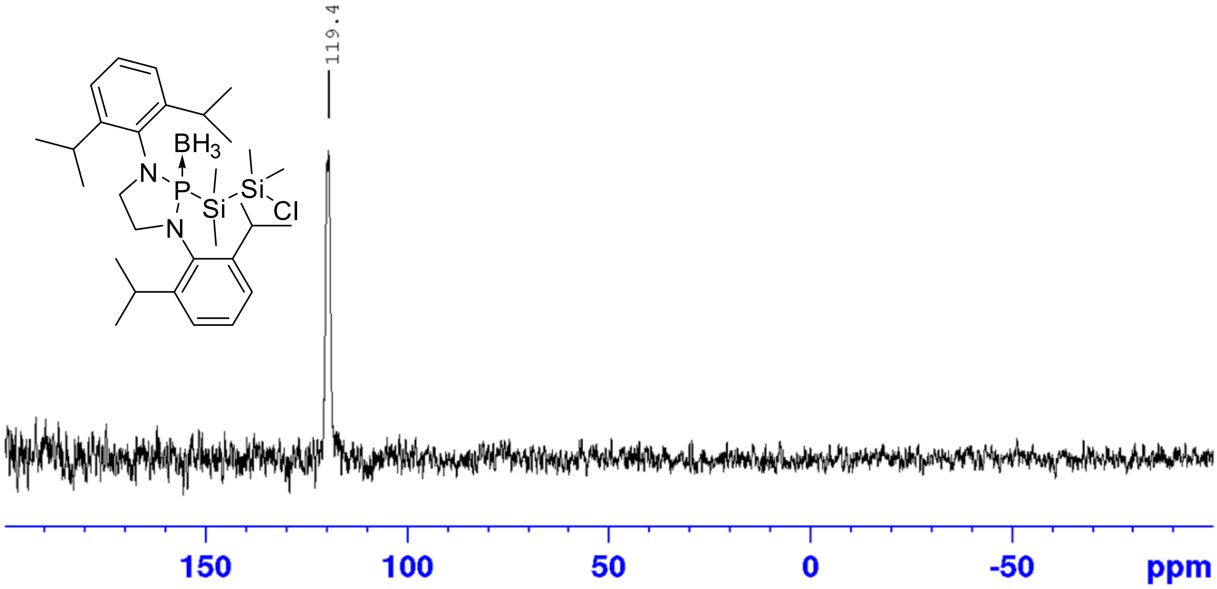

Figure S53: $^{31}P\{^{1}H\}$ NMR spectrum (C_6D_6) of **6e**.

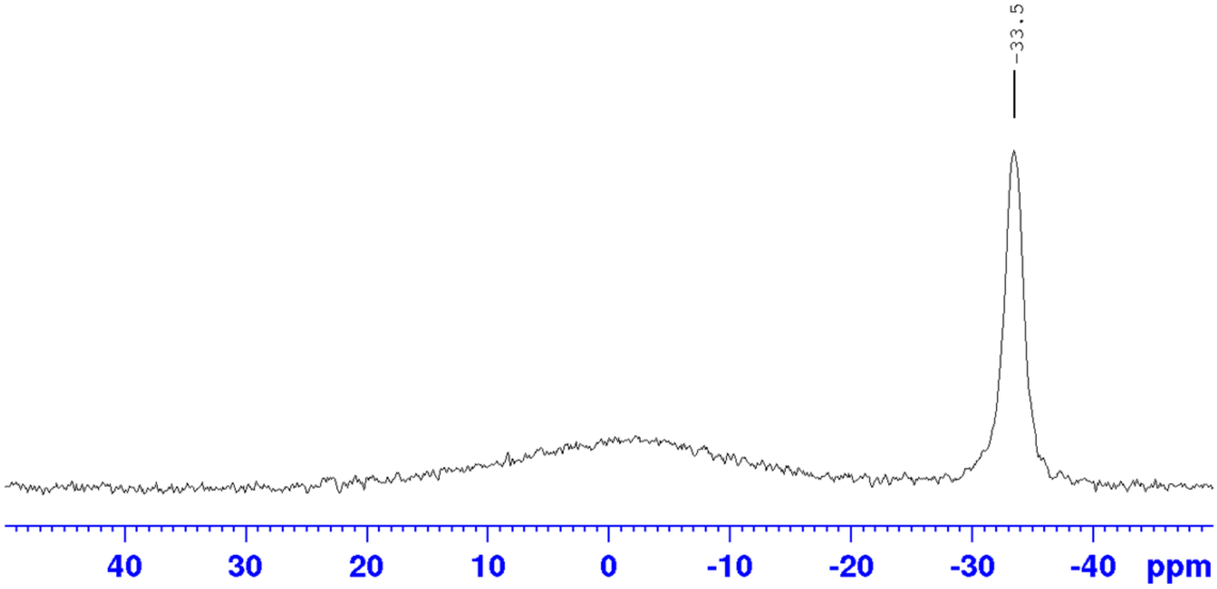

Figure S54: $^{11}B\{^{1}H\}$ NMR spectrum (C_6D_6) of **6e**. The broad bump is part of the probe background.

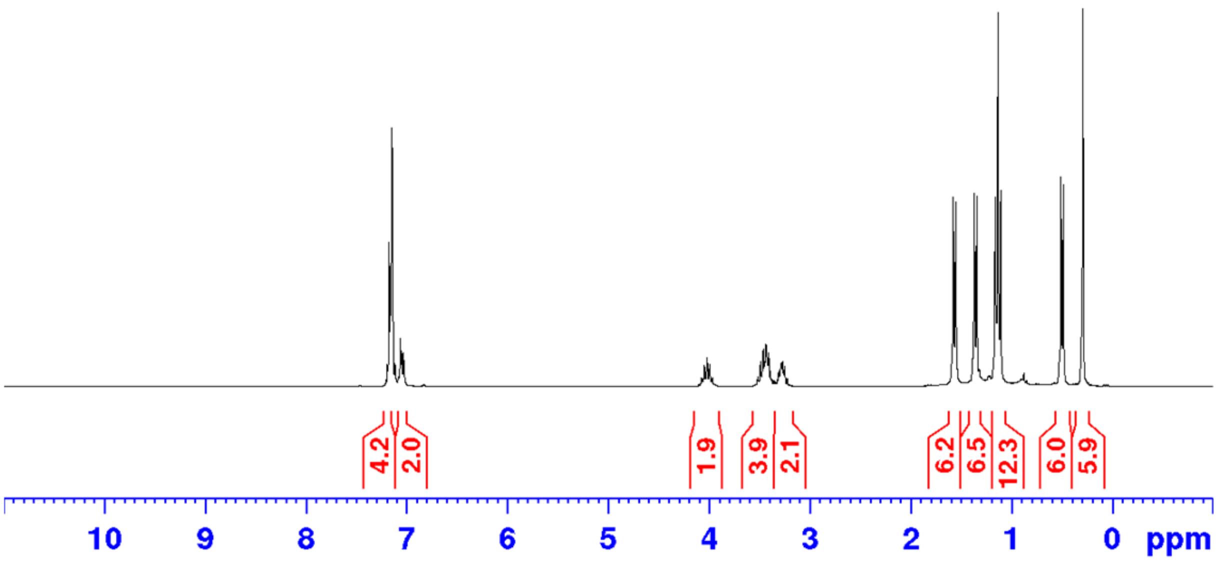

Figure S55: ^{1}H NMR spectrum (C_6D_6) of **6e**.

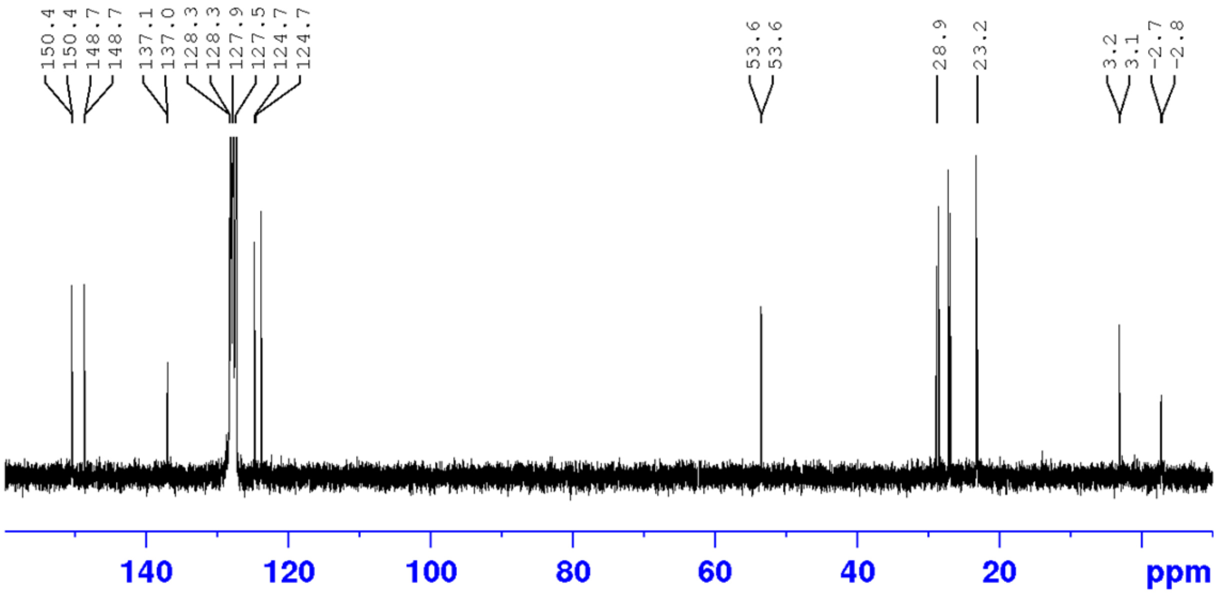

Figure S56: $^{13}C\{^{1}H\}$ NMR spectrum (C_6D_6) of **6e**.

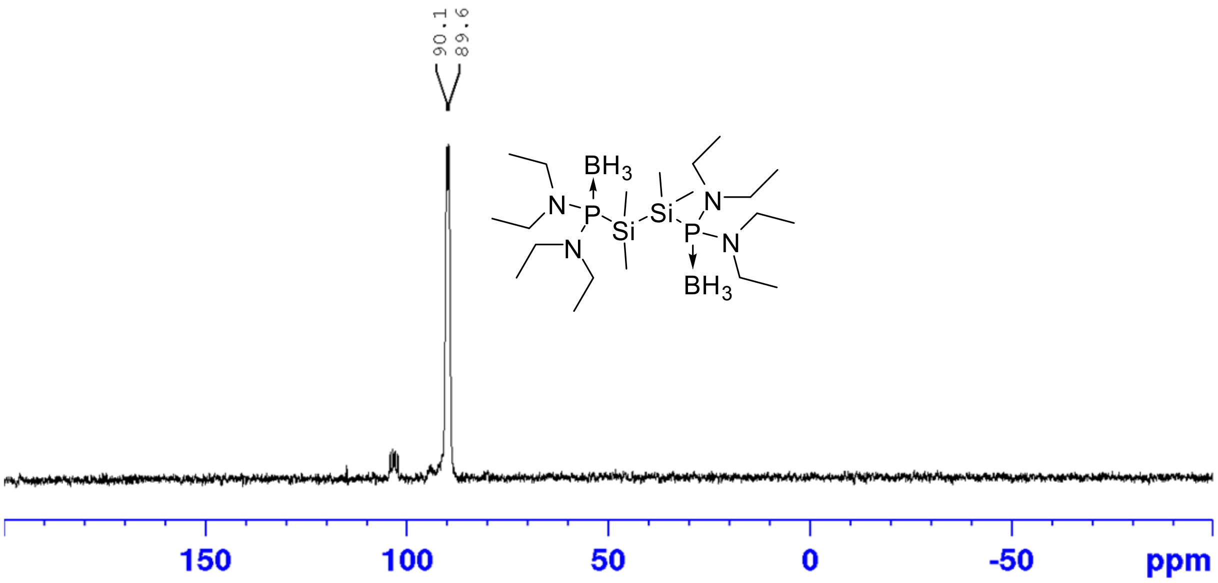

Figure S57: $^{31}P\{^{1}H\}$ NMR spectrum (C_6D_6) of **7**.

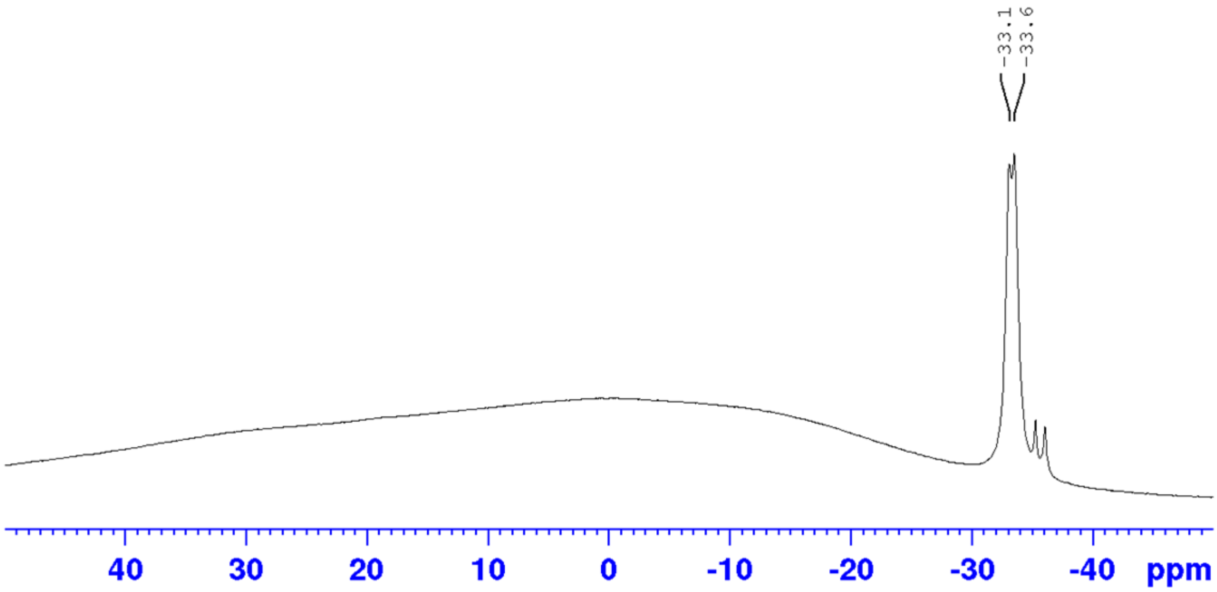

Figure S58: $^{11}B\{^{1}H\}$ NMR spectrum (C_6D_6) of **7**. The broad bump is part of the probe background.

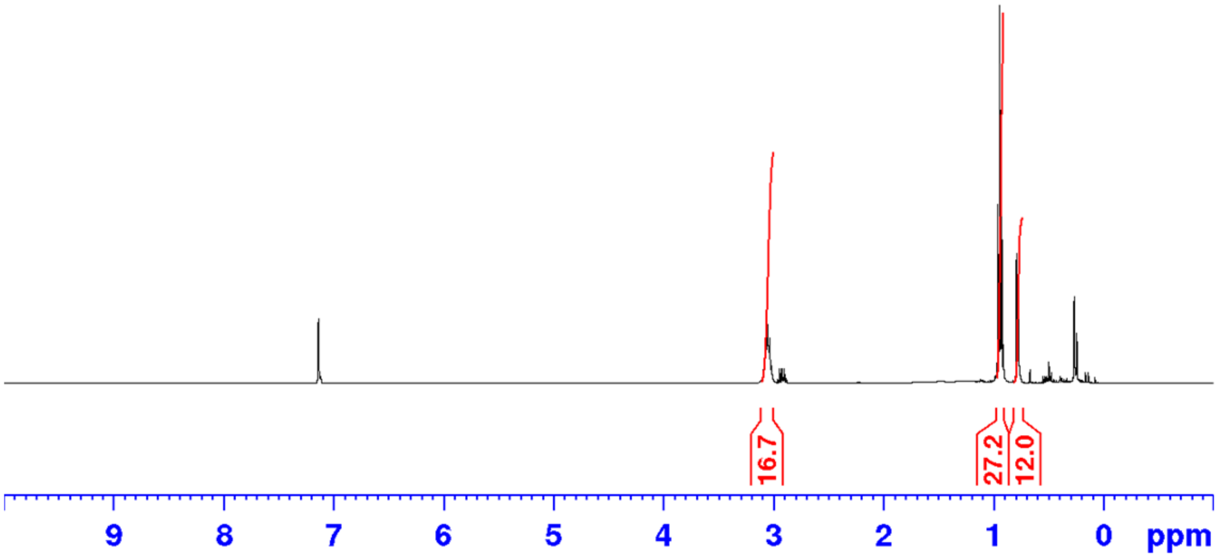

Figure S59: ^{1}H NMR spectrum (C_6D_6) of **7**.

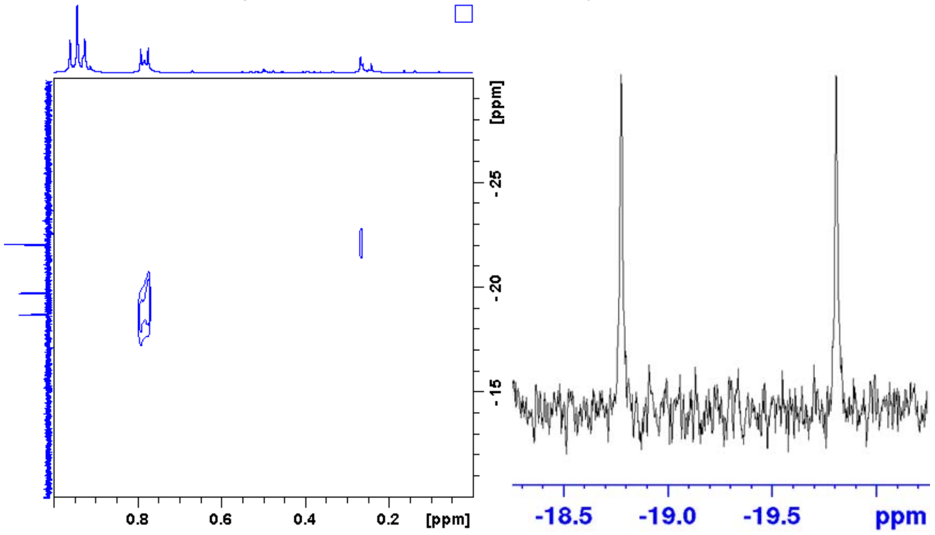

Figure S60: $^{1}H,^{29}Si$ gsHSQC and ^{29}Si-DEPT (vertical projection and expansion) NMR spectra (C_6D_6) of **7**. The correlation signal at 0.27/-22.0 ppm is due to silicon grease.

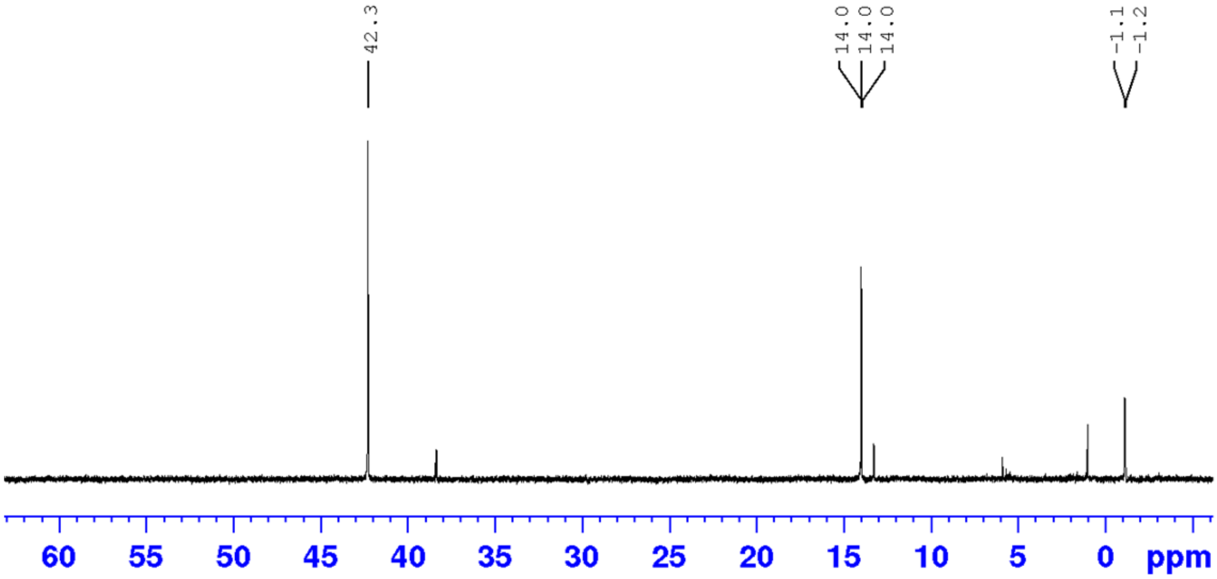

Figure S61: $^{13}C\{^1H\}$ NMR spectrum (C_6D_6) of **7**.

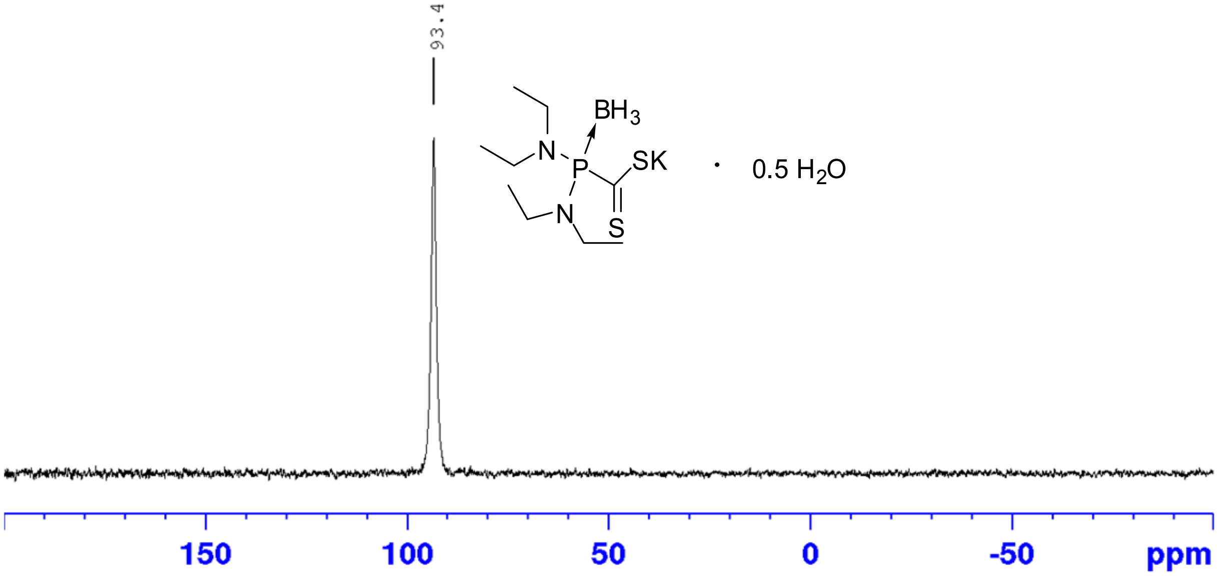

Figure S62: $^{31}P\{^1H\}$ NMR spectrum (C_6D_6) of **8**.

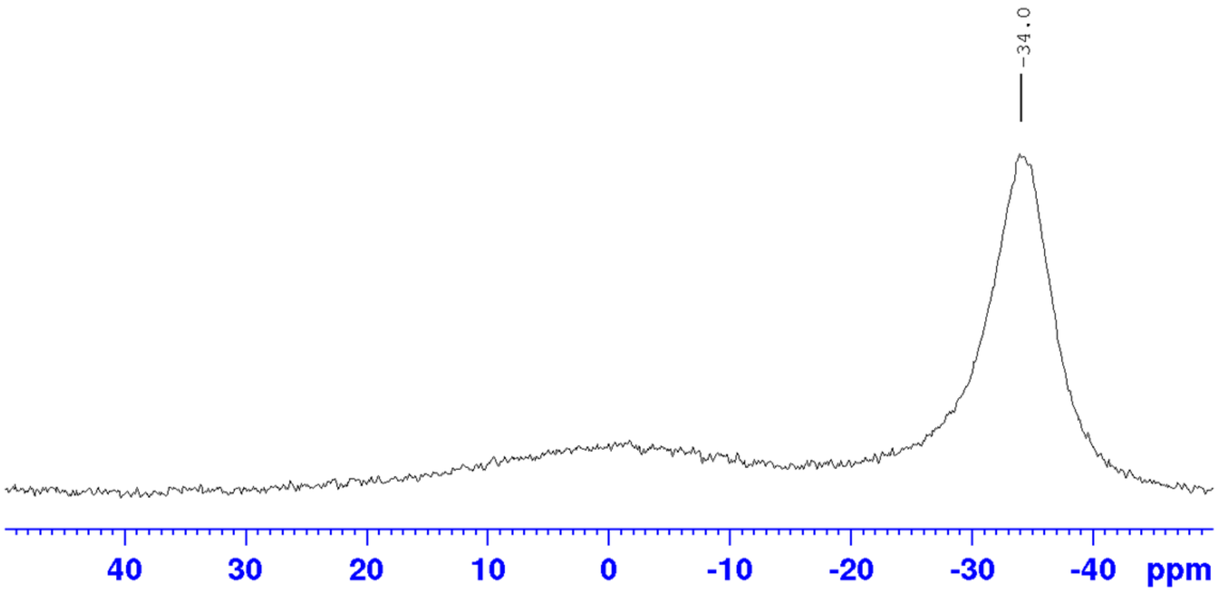

Figure S63: $^{11}B\{^1H\}$ NMR spectrum (C_6D_6) of **8**. The bump is part of the probe background signal.

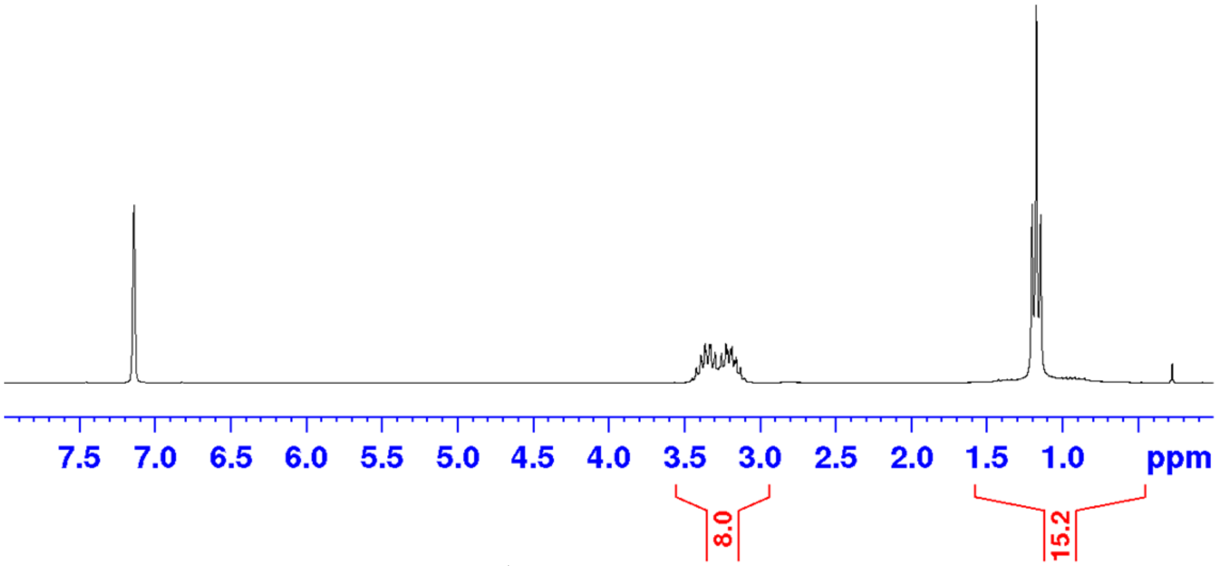

Figure S64: ^{1}H NMR spectrum (C_6D_6) of **8**.

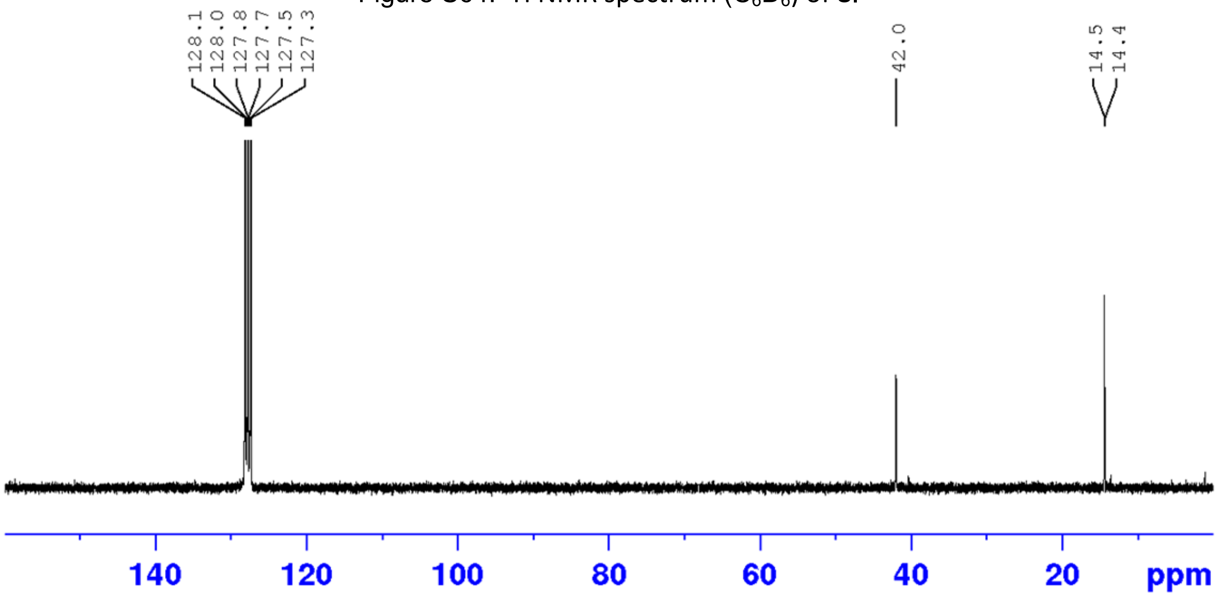

Figure S65: $^{13}C\{^{1}H\}$ NMR spectrum (C_6D_6) of **8**.
